# Cyanide Binding to [FeFe]‐Hydrogenase Stabilizes the Alternative Configuration of the Proton Transfer Pathway

**DOI:** 10.1002/anie.202216903

**Published:** 2023-01-10

**Authors:** Jifu Duan, Anja Hemschemeier, David J. Burr, Sven T. Stripp, Eckhard Hofmann, Thomas Happe

**Affiliations:** ^1^ Department of Plant Biochemistry Faculty of Biology and Biotechnology Photobiotechnology Ruhr University Bochum Universitätsstrasse 150 44801 Bochum Germany; ^2^ Department of Physics Experimental Biophysics and Space Sciences Freie Universität Berlin Arnimallee 14 14195 Berlin Germany; ^3^ Department of Biophysics Experimental Molecular Biophysics Freie Universität Berlin Arnimallee 14 14195 Berlin Germany; ^4^ Department of Biophysics Faculty of Biology and Biotechnology Protein Crystallography Ruhr University Bochum Universitätsstrasse 150 44801 Bochum Germany

**Keywords:** Cyanide, Hydrogen Bonds, Hydrogenase, Proton Transfer Pathway, X-Ray Diffraction

## Abstract

Hydrogenases are H_2_ converting enzymes that harbor catalytic cofactors in which iron (Fe) ions are coordinated by biologically unusual carbon monoxide (CO) and cyanide (CN^−^) ligands. Extrinsic CO and CN^−^, however, inhibit hydrogenases. The mechanism by which CN^−^ binds to [FeFe]‐hydrogenases is not known. Here, we obtained crystal structures of the CN^−^‐treated [FeFe]‐hydrogenase CpI from *Clostridium pasteurianum*. The high resolution of 1.39 Å allowed us to distinguish intrinsic CN^−^ and CO ligands and to show that extrinsic CN^−^ binds to the open coordination site of the cofactor where CO is known to bind. In contrast to other inhibitors, CN^−^ treated crystals show conformational changes of conserved residues within the proton transfer pathway which could allow a direct proton transfer between E279 and S319. This configuration has been proposed to be vital for efficient proton transfer, but has never been observed structurally.

## Graphical abstract

[FeFe]‐hydrogenases are highly efficient H_2_ biocatalysts that employ intrinsic CN^−^ ligands. Here, extrinsic CN^−^ is shown to bind at the open coordination site of the catalytic center. Additionally, new conformations within the proton transfer pathway that are complementary to those in the oxidized state H_ox_ are observed, providing a structural basis for understanding the molecular mechanisms of rapid proton shuttling within enzymes.

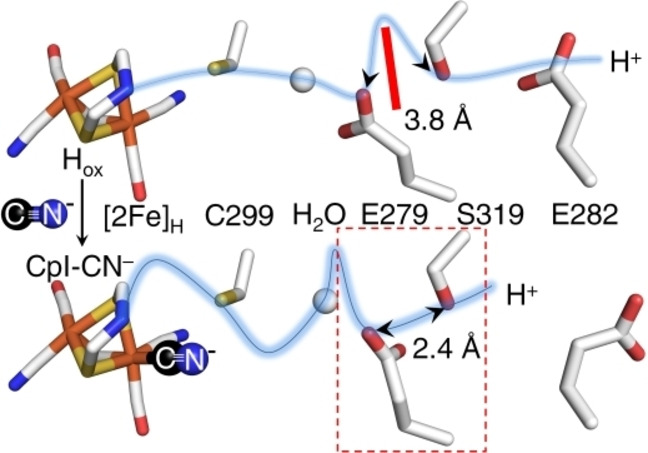

Hydrogenases are metalloenzymes that catalyze the interconversion between protons (H^+^), electrons (e^−^) and hydrogen (H_2_). Depending on the metal composition and configuration of the catalytic cofactor, they are classified as [Fe]‐, [NiFe]‐, and [FeFe]‐hydrogenases. Among them, [FeFe]‐hydrogenases exhibit the highest activity for both H_2_ production and oxidation.^[1]^ Their catalytic cofactor, termed H‐cluster, consists of a [4Fe4S] sub‐cluster ([4Fe]_H_) and a diiron moiety ([2Fe]_H_) bridged through a cysteine residue (Figure 1A).[^2^, ^3^] The diiron atoms of [2Fe]_H_ proximal and distal to [4Fe]_H_ (Fe_p_ and Fe_d_) are bridged by an aza‐dithiolate whose bridgehead amine and Fe_d_ constitute a frustrated Lewis pair for H_2_ activation.[^4^, ^5^, ^6^] The amine base is connected to the protein surface through a conserved proton transfer pathway (PTP) for rapid H^+^ shuttling.[^7^, ^8^, ^9^, ^10^] Fe_p_ and Fe_d_ are each coordinated by a terminal carbon monoxide (tCO) and a terminal cyanide (tCN^−^), and they share one additional CO in the bridging position (μCO). This configuration leaves an open coordination site (OCS) at Fe_d_ for substrate binding (Figure 1A), as revealed by the structure of the oxidized enzyme (H_ox_),^[11]^ also referred to as the active ready state.

**Figure 1:**
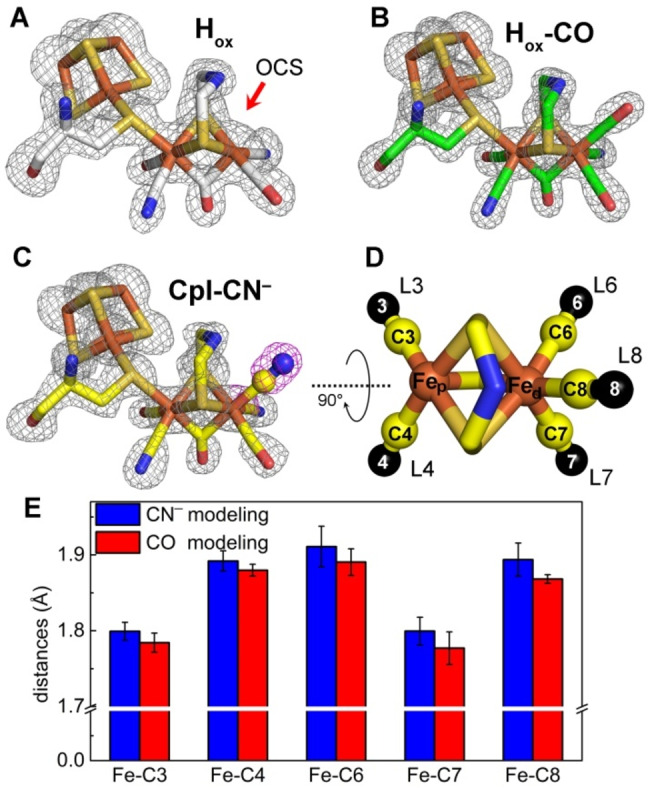
Structures of the CpI H‐cluster and structural assignment of terminal CO and CN^−^ ligands. A to C show H‐clusters from chains B of CpI in the H_ox_ state (PDB: 4XDC)^[11]^ (A), H_ox_ crystals treated with CO (H_ox_‐CO‐1; PDB: 8ALN) (B) and CN^−^ treated CpI H_ox_ crystals (CpI‐CN^−^‐1; PDB: 8AP2) (C). Simulated annealing omit maps were contoured at 3, 4 and 3.5 σ for A, B and C, respectively. H‐clusters of additional chains and structures are presented in Figure S2. D) depicts the [2Fe]_H_ moiety of one CN^−^ treated crystal (PDB: 8AP2) to illustrate the labeling employed in (E). Terminal Fe−CO/CN^−^ ligands are abbreviated as L3, L4, L6, L7 and L8, and the respective C atoms are numbered accordingly (C3, C4, C6, C7 and C8). E) shows the refined distances between each Fe and the C atoms of its terminal ligands when modeling them as all CN^−^ (blue bars) or all CO (red bars).

The molecules CO and CN^−^ are well‐known to bind to metalloenzymes. Intriguingly, at the same time, hydrogenases employ them as natural Fe ligands to ensure the low spin and low oxidation states of the active site metals.^[12]^ The inhibitory effect of extrinsic CO on [FeFe]‐hydrogenases has been well characterized applying different methods, including X‐ray crystallography,[^13^, ^14^] however, little is known about the interaction between CN^−^ and [FeFe]‐hydrogenases.^[15]^

In this study, we performed an X‐ray crystallography study of the CN^−^‐bound form of the [FeFe]‐hydrogenase CpI from *Clostridium pasteurianum*. Two crystals of 1.39 Å and 1.5 Å, termed CpI‐CN^−^‐1 and CpI‐CN^−^‐2 in the following, were analyzed in depth. They were compared to two structures of CO‐treated CpI for which we obtained higher resolutions (1.34 Å and 1.52 Å; termed H_ox_‐CO‐1 and H_ox_‐CO‐2 hereafter) than previously reported.[^13^, ^14^] This allowed a more direct comparison among these four structures as well as with a previously published structure of untreated CpI in the H_ox_ state (PDB: 4XDC).[^11^, ^16^] All crystals were grown according to our previous protocols,[^8^, ^11^] and the CO‐ and CN^−^‐bound states were obtained by introducing these molecules into the crystallization reservoirs (see details in the materials and methods section). The CO/CN^−^ treatment did not significantly change the crystal parameters (Table S1). The protein molecules were packed in the same space group of P1 21 1, and each asymmetric unit contained two chains, A and B. The overall structures of CO/CN^−^‐treated CpI are very similar to each other and to that of CpI in the H_ox_ state (with root mean square deviations for Cα atoms of 0.372–0.736 Å; Figure S1; Tables S1 and S2).

The four newly obtained structures feature strong electron densities at the OCS at Fe_d_ that are not present in the structure of untreated CpI (Figure 1; Figure S2). The refined occupancies indicate that the OCS is fully occupied by an extrinsic ligand in CN^−^‐treated CpI, whereas only 71 to 93 % of the OCS of CO‐treated crystals hold an extrinsic ligand (Table S3). The latter is possibly due to an insufficient partial pressure of CO in the crystallization wells. To assign specific molecules to these electron densities we followed different modeling approaches. Modeling with a H_2_O/OH^−^ ligand, which was assigned in earlier CpI structures,[^2^, ^17^] resulted in considerable unexplained electron densities (Figure S3), suggesting that the densities observed in CO‐ and CN^−^‐treated crystals do not represent H_2_O/OH^−^ molecules. Instead, we obtained much better fitting by modeling with CN^−^ or CO diatomic ligands (Figure S3).

CO binds to the OCS of the H‐cluster,[^13^, ^14^] and CO and CN^−^ are isoelectronic, making it challenging to distinguish them by electron density analysis. However, high resolution structures of hydrogenases and the chemical structure of the [2Fe]_H_ mimic show that the distances between active‐site Fe ions and the intrinsic terminal CO and CN^−^ ligands differ and amount to about 1.75 Å and 1.9 Å, respectively.[^17^, ^18^, ^19^] To assign CO and CN^−^ ligands in the structure of CN^−^‐treated CpI, we thus performed unbiased structure refinements and modeled the five terminal ligands (L3, L4, L6, L7 and L8; Figure 1D) as either all CO or all CN^−^ ligands (the details of the proceeding are explained in the materials and methods section). Figure 1E presents the refined distances between each Fe ion of the [2Fe]_H_ moiety and the carbon atoms of their direct ligands (denoted as C3, C4, C6, C7 and C8) within the 1.39 Å data set of CN^−^‐treated crystals (CpI‐CN^−^‐1). For each ligand, CN^−^ and CO modeling obtained very similar bond lengths, although the latter resulted in slightly shorter distances. Both modeling approaches resulted in Fe−C3 and Fe−C7 distances of about 1.78 Å on average, while those between the Fe ions and C4, C6 and C8 were averaged to amount to 1.9 Å. These values thus recapitulate Fe−CO and Fe−CN^−^ distances observed in previous high‐resolution structures of hydrogenases as well as the [2Fe]_H_ chemical mimic.[^17^, ^18^, ^19^] Therefore, we are confident to assign the intrinsic ligands as CO‐ (L3 and L7) or CN^−^ ligands (L4, L6) and conclude that the molecule bound at the OCS (L8) is indeed a CN^−^ ligand. Except this extra CN^−^ molecule at position L8, the four natural terminal ligands are in the same configuration as in CpI in the H_ox_ state.[^17^, ^20^] Note that we applied the same unbiased refinement strategy to calculate bond‐lengths in the 1.34 Å data‐set of CO‐treated crystals (H_ox_‐CO‐1). Despite the high resolution, we could not obtain conclusive results. We assume that this is due to lower occupancies of the [2Fe]_H_ moiety and the extrinsic ligand (Table S3).

Fe−CO/CN^−^ ligands exhibit infrared (IR) absorbance, and their absorption frequencies are sensitive to the electronic configuration of the Fe atoms as well as to isotopic labeling.[^1^, ^21^] We therefore performed an IR spectroscopic investigation of the CpI crystals (Figure S4A). The untreated crystals showed a typical H_ox_ state^[16]^ while the crystals subjected to CN^−^ treatment exhibited a new pattern with a third Fe−CN^−^ band at 2112 cm^−1^. When ^13^CN^−^ was used instead of ^12^CN^−^, this signal underwent a selective shift to lower energies by 49 cm^−1^, validating the assignment of this band. These data confirm that extrinsic CN^−^ binds to the H‐cluster. Notably, compared to the H_ox_ state, the absorbance of all ligands was shifted to higher energies, indicating a further oxidation of the [2Fe]_H_ site. Very similar results were obtained for the [FeFe]‐hydrogenase HydA1 from *Chlamydomonas reinhardtii* (Figure S4B), implying extrinsic CN^−^ may bind to the H‐cluster of all [FeFe]‐hydrogenases. We additionally tested whether CN^−^ inhibits the activity of CpI. Indeed, in the presence of 20 mM KCN, H_2_ production activity decreased by over 64 % (Figure S5). In view of the structural and IR data, this suggests that CN^−^ inhibits catalysis by binding to the OCS.

We then inspected CN^−^ bound structures with a focus on catalytically important residues. Amino acid side chains directly adjacent to the H‐cluster show the same conformations as in the structures of H_ox_ and H_ox_‐CO. For example, the abundant H‐bond interactions of the intrinsic CO and CN^−^ ligands with the protein backbone^[20]^ are almost identical in CpI‐CN^−^‐1 and H_ox_ (Figure S6). However, we observed noticeable deviations in the PTP (Figure 2, Figures S7 and S8). This highly conserved pathway for catalytic H^+^ transport is formed by C299, E279, S319 and E282, reaching from the amine group of [2Fe]_H_ to the protein surface, and it additionally contains two water molecules (Wat1 and Wat2), between, and H‐bonding with, C299 and E279 (Figure 2).[^7^, ^8^] In the two structures CpI‐CN^−^‐1 and CpI‐CN^−^‐2, the distances between C299 and the amine group of the [2Fe]_H_ subcluster are larger than those in H_ox_ in both chains A and B (Figure 2B; Figures S7 and S8). The side chain of E279 is slightly twisted and consequently closer to S319, whereas the distance between E279 and Wat1 increases in several cases (Figure 2B; Figures S7 and S8). Wat1 and Wat2 are also shifted; in all cases, Wat1 lies about 0.6 to 1 Å closer to the surface. Consequently, the H‐bond length between C299 and Wat1 is longer than in the H_ox_ state (Figure 2B; Figures S7 and S8). The electron densities of E282 revealed varying conformations. In chain B of CpI‐CN^−^‐2, E282 is twisted towards the surface and out of H‐bond length to S319. In chain B of CpI‐CN^−^‐1 and chain A of CpI‐CN^−^‐2, this residue was modeled in two conformations (Figures S7 and S8).

**Figure 2:**
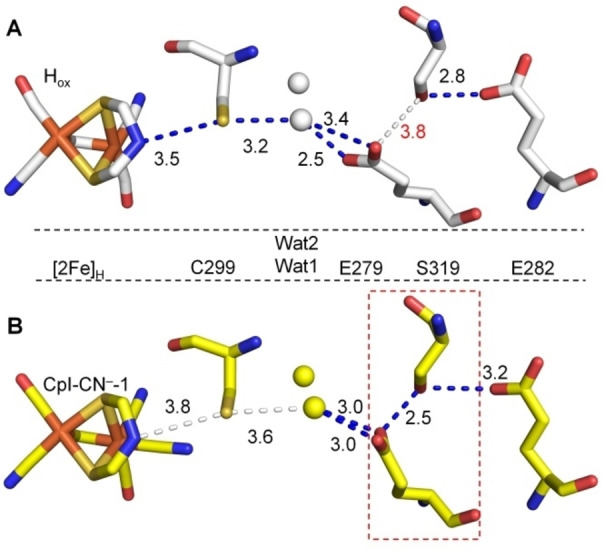
Comparison of H‐bond patterns within the H^+^ transfer pathway in structures of CpI in the H_ox_ (A) and the cyanide‐bound state (B). Carbon atoms are colored white and yellow for CpI‐H_ox_ (PDB: 4XDC, chain A)^[11]^ (A) and CpI‐CN^−^‐1 (PDB: 8AP2, chain A) (B), respectively. Water molecules are presented as spheres and colored as the C atoms. Numbers between adjacent atoms represent distances in Å. Blue dashed lines indicate distances suitable for strong to medium H‐bond interactions (up to 3.5 Å), white dashed lines indicate weak H‐bonds (distances of 3.6 Å and longer). The short distance between E279 and S319 observable in CN^−^‐treated crystals is highlighted by the red dashed box.

The structural changes in PTP elements observed in our CN^−^‐treated crystals have been proposed to be necessary for efficient catalytic H^+^ transport, but they have never been observed structurally in wild type [FeFe]‐hydrogenases. In the H_ox_ state, the PTP has been described as being discontinuous, and in the H_ox_ crystal structure, the distance between E279 and S319 (3.6–3.8 Å) indeed indicates a barrier for efficient H^+^ transport (Figure 2B; Figure S8).[^9^, ^11^, ^22^] Theoretical studies proposed a conformational flexibility of side chains within the pathway by which the enzyme overcomes the potential bottleneck between E279 and S319.[^23^, ^24^] Previously, we analyzed H^+^ transport within [FeFe]‐hydrogenase HydA1 by IR difference spectroscopy. The data suggested that upon reduction of the H‐cluster from the H_ox_ to the H_red_ state, HydA1 E141, which corresponds to CpI E279, forms a stronger interaction with HydA1 S189 (corresponding to CpI S319) at the expense of the interaction with Wat1.^[9]^ Here, rather unexpectedly, the CN^−^ bound structures show configurations that support these predicted conformational changes (Figure 2B; Figures S7 and S8).

To the best of our knowledge, conformational changes of PTP residues have never been observed in structures of untreated CpI wild type proteins. They are also absent from three independently prepared structures obtained by CO treatment^[13]^ (Figures S9 and S10) as well as from the sulfide (SH^−^) ‐bound *Desulfovibrio desulfuricans* [FeFe]‐hydrogenase.^[25]^ In the context of H^+^ transport, however, it is noteworthy that the new H‐bond pattern in the CN^−^‐treated crystals shows similarities to a conformation observed in our previously characterized CpI variant E279D (PDB: 6YF4) (Figure S8).[^8^, ^10^] This enzyme variant has a lower pH optimum for H_2_ production, but retains significant activity in both directions of H_2_ oxidation and H^+^ reduction compared to the wild type protein.[^8^, ^10^] The shorter side chain of the introduced D279 residue is in H‐bond length to both S319 and Wat1, but significantly closer to S319. Slight shifts of C299 and Wat1 lead to a 4.0 Å gap between C299 and Wat1 (Figure S8). Comparing the discussed structural data from previous studies and this work, it appears that they represent snapshots of two discontinuous PTPs that are likely to alternate during active catalysis in order to provide the necessary continuous pathway for rapid H^+^ shuttling.

For the time being, we do not have a robust explanation for why the binding of CN^−^ to CpI crystals in the H_ox_ results in the observed conformational changes. It seems possible that the chemistry of CN^−^ (which should be mostly in the protonated HCN state under the conditions applied for crystal treatment), influences the H^+^ equilibrium in a way that stabilizes the new conformations. Alternatively, CN^−^ might directly or indirectly influence the (redox) state(s) of the H‐cluster. Our IR data indeed hint at a further oxidation of the diiron site. In the case of the active site cofactor of [NiFe]‐hydrogenases, CN^−^ (or HCN) promotes the formation of the “unready” Ni−B state over the Ni‐SI_r_ state, which involves the oxidation of Ni^II^ to Ni^III^ and the formation of a negatively charged bridging hydroxide ligand between the Ni and Fe ions, shifting the equilibrium towards inactive states.^[26]^

Characterizing the interaction between [FeFe]‐hydrogenases and cyanide therefore promises to provide deeper mechanistic insights into the catalytic mechanism of H_2_ turnover and, more specifically, of the rapid H^+^ transfer that enables [FeFe]‐hydrogenases to reach such high efficiencies. The high‐resolution structural data presented here provide a tangible basis for interpreting data obtained by other methods.

## Conflict of interest

The authors declare no conflict of interest.

## Supporting information

As a service to our authors and readers, this journal provides supporting information supplied by the authors. Such materials are peer reviewed and may be re‐organized for online delivery, but are not copy‐edited or typeset. Technical support issues arising from supporting information (other than missing files) should be addressed to the authors.

Supporting Information

Supporting Information

Supporting Information

Supporting Information

Supporting Information

## Data Availability

The data that support the findings of this study are available from the corresponding author upon reasonable request.

## References

[1] W. Lubitz , H. Ogata , O. Rudiger , E. Reijerse , Chem. Rev. 2014, 114, 4081–4148. 24655035 10.1021/cr4005814

[2] J. W. Peters , W. N. Lanzilotta , B. J. Lemon , L. C. Seefeldt , Science 1998, 282, 1853–1858. 9836629 10.1126/science.282.5395.1853

[3] Y. Nicolet , C. Piras , P. Legrand , C. E. Hatchikian , J. C. Fontecilla-Camps , Structure 1999, 7, 13–23. 10368269 10.1016/s0969-2126(99)80005-7

[4] D. W. Stephan , G. Erker , Angew. Chem. Int. Ed. 2010, 49, 46–76; 10.1002/anie.200903708 20025001

[5] G. Berggren , A. Adamska , C. Lambertz , T. R. Simmons , J. Esselborn , M. Atta , S. Gambarelli , J. M. Mouesca , E. Reijerse , W. Lubitz , T. Happe , V. Artero , M. Fontecave , Nature 2013, 499, 66–69. 23803769 10.1038/nature12239 PMC3793303

[6] J. Esselborn , C. Lambertz , A. Adamska-Venkates , T. Simmons , G. Berggren , J. Noth , J. Siebel , A. Hemschemeier , V. Artero , E. Reijerse , M. Fontecave , W. Lubitz , T. Happe , Nat. Chem. Biol. 2013, 9, 607–609. 23934246 10.1038/nchembio.1311 PMC3795299

[7] A. J. Cornish , K. Gartner , H. Yang , J. W. Peters , E. L. Hegg , J. Biol. Chem. 2011, 286, 38341–38347. 21900241 10.1074/jbc.M111.254664 PMC3207428

[8] J. Duan , M. Senger , J. Esselborn , V. Engelbrecht , F. Wittkamp , U. P. Apfel , E. Hofmann , S. T. Stripp , T. Happe , M. Winkler , Nat. Commun. 2018, 9, 4726. 30413719 10.1038/s41467-018-07140-x PMC6226526

[9] M. Senger , V. Eichmann , K. Laun , J. Duan , F. Wittkamp , G. Knor , U. P. Apfel , T. Happe , M. Winkler , J. Heberle , S. T. Stripp , J. Am. Chem. Soc. 2019, 141, 17394–17403. 31580662 10.1021/jacs.9b09225 PMC6823627

[10] O. Lampret , J. Duan , E. Hofmann , M. Winkler , F. Armstrong , T. Happe , Proc. Natl. Acad. Sci. USA 2020, 117, 20520–20529. 32796105 10.1073/pnas.2007090117 PMC7456106

[11] J. Esselborn , N. Muraki , K. Klein , V. Engelbrecht , N. Metzler-Nolte , U. P. Apfel , E. Hofmann , G. Kurisu , T. Happe , Chem. Sci. 2016, 7, 959–968. 29896366 10.1039/c5sc03397g PMC5954619

[12] A. Silakov , E. J. Reijerse , S. P. Albracht , E. C. Hatchikian , W. Lubitz , J. Am. Chem. Soc. 2007, 129, 11447–11458. 17722921 10.1021/ja072592s

[13] B. J. Lemon , J. W. Peters , Biochemistry 1999, 38, 12969–12973. 10529166 10.1021/bi9913193

[14] B. J. Lemon , J. W. Peters , J. Am. Chem. Soc. 2000, 122, 3793–3794.

[15] M. W. W. Adams , L. E. Mortenson , J. S. Chen , Biochim. Biophys. Acta Rev. Bioenerg. 1980, 594, 105–176. 10.1016/0304-4173(80)90007-5 6786341

[16] S. Morra , J. Duan , M. Winkler , P. A. Ash , T. Happe , K. A. Vincent , Dalton Trans. 2021, 50, 12655–12663. 34545877 10.1039/d1dt02219a PMC8453692

[17] A. S. Pandey , T. V. Harris , L. J. Giles , J. W. Peters , R. K. Szilagyi , J. Am. Chem. Soc. 2008, 130, 4533–4540. 18324814 10.1021/ja711187e

[18] H. Li , T. B. Rauchfuss , J. Am. Chem. Soc. 2002, 124, 726–727. 11817928 10.1021/ja016964n

[19] H. Ogata , K. Nishikawa , W. Lubitz , Nature 2015, 520, 571–574. 25624102 10.1038/nature14110

[20] O. Lampret , A. Adamska-Venkatesh , H. Konegger , F. Wittkamp , U. P. Apfel , E. J. Reijerse , W. Lubitz , O. Rudiger , T. Happe , M. Winkler , J. Am. Chem. Soc. 2017, 139, 18222–18230. 29179539 10.1021/jacs.7b08735

[21] M. Senger , S. Mebs , J. Duan , F. Wittkamp , U. P. Apfel , J. Heberle , M. Haumann , S. T. Stripp , Proc. Natl. Acad. Sci. USA 2016, 113, 8454–8459. 27432985 10.1073/pnas.1606178113 PMC4968730

[22] J. H. Artz , O. A. Zadvornyy , D. W. Mulder , S. M. Keable , A. E. Cohen , M. W. Ratzloff , S. G. Williams , B. Ginovska , N. Kumar , J. Song , S. E. McPhillips , C. M. Davidson , A. Y. Lyubimov , N. Pence , G. J. Schut , A. K. Jones , S. M. Soltis , M. W. W. Adams , S. Raugei , P. W. King , J. W. Peters , J. Am. Chem. Soc. 2020, 142, 1227–1235. 31816235 10.1021/jacs.9b08756 PMC8653774

[23] H. Long , P. W. King , C. H. Chang , J. Phys. Chem. B 2014, 118, 890–900. 24405487 10.1021/jp408621r

[24] R. C. Puthenkalathil , B. Ensing , J. Phys. Chem. B 2022, 126, 403–411. 35007078 10.1021/acs.jpcb.1c08124 PMC8785182

[25] P. Rodríguez-Maciá , L. M. Galle , R. Bjornsson , C. Lorent , I. Zebger , Y. Yoda , S. P. Cramer , S. DeBeer , I. Span , J. A. Birrell , Angew. Chem. Int. Ed. 2020, 59, 16786–16794; 10.1002/anie.202005208 PMC7540559 32488975

[26] S. V. Hexter , M. W. Chung , K. A. Vincent , F. A. Armstrong , J. Am. Chem. Soc. 2014, 136, 10470–10477. 25003708 10.1021/ja504942h

