# Supplementary material for: Cyanide Binding to [FeFe]‐Hydrogenase Stabilizes the Alternative Configuration of the Proton Transfer Pathway
Source: Angew Chem Int Ed Engl. 2023 Jan 10;62(7):e202216903. doi: 10.1002/anie.202216903 (PMC10107461; doi:10.1002/anie.202216903)
Supplement: Supplementary file 1 — Supporting Information [file ANIE-62-0-s004.pdf]

Angewandte Chemie
Eine Zeitschrift der Gesellschaft Deutscher Chemiker

Supporting Information

Cyanide Binding to [FeFe]-Hydrogenase Stabilizes the Alternative Configuration of the Proton Transfer Pathway

J. Duan, A. Hemschemeier, D. J. Burr, S. T. Stripp, E. Hofmann, T. Happe**

Table of Contents

Materials and Methods. (Page 2)
Figure S1. Superimposition of structures of CpI in various states. (Page 4)
Figure S2. H-cluster structures of H_{ox}-CO-1, H_{ox}-CO-2, CpI-CN^--1 and CpI-CN^--2. (Page 4)
Figure S3. Comparison of modeling water molecules or CN^-/CO at the open coordination site (OCS) of the H-clusters. (Page 5)
Figure S4. Infrared spectra of CpI single crystals and HydA1 protein film upon $^{12}CN^-$/$^{13}CN^-$ treatment. (Page 6)
Figure S5. H_2 production activity assay of CpI in the presence of KCN. (Page 6)
Figure S6. Polar interactions between the CN^-/CO ligands and the protein environment. (Page 7)
Figure S7. Structural comparison of elements of the proton transfer pathway (PTP) in CpI in the H_{ox}- and the CpI-CN^- states. (Page 8)
Figure S8. Distances between H-bond partners of the proton transfer pathway (PTP) within structures H_{ox}, CpI-CN^--1, CpI-CN^--2 and CpI-E279D. (Page 9)
Figure S9. Proton transfer pathway (PTP) structures of CpI in the H_{ox}- and the H_{ox}-CO states. (Page 10)
Figure S10. Comparison of proton transfer pathway (PTP) structures of CpI in the H_{ox}- and the H_{ox}-CO states. (Page 10)

Table S1. Statistics for crystallographic data collection and refinement. (Page 12)
Table S2. Calculated RMSD (root mean square deviations in Å) of the C-α atoms when superimposing the structures with 4XDC. (Page 13)
Table S3. Group occupancies of the $[2Fe]_H$ moiety and extrinsic CO/CN^- ligands. (Page 13)
Table S4. Refined distances of Fe-CO/CN^- modeled with different values of the Fe-C-O/N angle. (Page 13)

References (Page 14)

Materials and Methods

Protein preparation

CpI/HydA1 lacking the $[2Fe]_H$ subcluster, termed apo-CpI/HydA1, were heterologously produced in *Escherichia coli* BL21(DE3) ΔiscR according to previously established protocols without co-expression of the maturase-encoding genes.[1] Apo-CpI/HydA1 were purified by Strep-tag II affinity chromatography (IBA Lifesciences, www.iba-lifesciences.com/) under strictly anoxic conditions in a glove box (Coy; https://coylab.com/) and subsequently maturated to the holo-form *in vitro* with the artificially synthesized $[2Fe]_H$ complex ($Fe_2[\mu\text{-}(SCH_2)_2NH](CN)_2(CO)_4[Et_4N]_2$)[2] as described before.[3] Excess of free $[2Fe]_H$ complex was removed by size exclusion chromatography using NAP-10 columns (GE healthcare; www.gehealthcare.de/). For protein crystallization, the CpI holo-protein was concentrated to 15 mg × ml^{-1} in 0.1 M Tris-HCl (pH 8.0) buffer supplemented with 2 mM sodium dithionite.

Protein crystallization and carbon monoxide (CO) or cyanide (CN^-) treatment of crystals

Crystallization was done according to our previously described hanging drop method.[4,5] Briefly, 2 µl of holo-CpI (15 mg × ml^{-1}) were mixed with 2 µl of crystallization solution on the glass cover. The glass cover was then put on the reservoir filled with 500 µl of crystallization solution (0.1 M MES (2-(N-morpholino) ethanesulfonic acid)-NaOH (pH 6.0), 0.4 M $MgCl_2$, 19-22 % PEG 4000 (polyethylene glycol of a molecular weight of ca. 4000 Da) and 18-21 % glycerol) and incubated under anoxic conditions at 4°C. After approximately two weeks, full sized CpI crystals (0.15 to 0.3 mm in the longest dimension) were obtained. Untreated control crystals were directly flash-frozen in liquid nitrogen employing cryo-loops.
For CO treatment, the glass cover holding CpI crystals was opened, 1 ml of 100 % CO gas was injected directly into the reservoir solution using a gas-tight syringe and the glass cover was immediately sealed. CN^- was introduced in a similar manner. KCN powder was rapidly dissolved in the crystallization solution to reach a final concentration of 100 mM and quickly pipetted into the reservoir of already grown crystals. The glass cover was immediately sealed. It was expected that HCN, formed from KCN in the solution, would diffuse into the protein crystal in the hanging drop. According to the chemical equilibrium and the volume of the reservoir, the final concentration of HCN (including CN^-) in the sealed crystallization environment was approximately 12.5 mM. The crystals were incubated in the dark for three days or three to four hours after CO or CN^- addition, respectively, before flash freezing the crystals as described above. All crystals were prepared independently (protein preparation, crystallization and diffraction).

X-ray diffraction and structure determination

X-ray diffraction data were collected at 100 K at different beamlines (Table S1). Diffraction data were processed using XDS.[6] Phenix[7] and Coot[8] were employed for molecular replacement using PDB 4XDC as the input model,[4] refinement and manual inspection and correction. Group occupancy refinement (with more than five iterative cycles) was done to determine the occupancies of the $[2Fe]_H$ moiety and extrinsic CO or CN^- ligands (Table S3). Anisotropic B factors for the protein molecule or ligands (but not water molecules) were introduced for refinement of the structures of H_{ox}-CO-1 (PDB: 8ALN) and CpI-CN^--1 (PDB: 8AP2) due to their higher resolutions (1.34 Å and 1.39 Å, respectively). The crystallographic statistics are summarized in Table S1. The corresponding structure factors and coordinates were deposited in the PDB under the following accession codes: H_{ox}-CO-1 (8ALN), H_{ox}-CO-2 (8AIO), CpI-CN^--1 (8AP2) and CpI-CN^--2 (8AJ6).

The bond lengths between the Fe ions of the $[2Fe]_H$ subcluster and the C atoms of their terminal CO or CN^- ligands were used as fingerprints to distinguish CO- from CN^- ligands.[2,9,10] To accurately refine the lengths of Fe-CO or Fe-CN^- bonds, the following refinement strategy was applied. A well-refined model (very close to the final model) was selected and the four intrinsic terminal CO/CN^- ligands as well as the (fifth) extrinsic ligand of $[2Fe]_H$ were modified into either all CO or all CN^- ligands for systematic comparison. Phenix geometry minimization [7] was used to set all the terminal Fe-CO/CN^- ligands with the following parameters: bond lengths of terminal Fe-CO/CN^- as 1.843 Å, angle of terminal Fe-C-O/N as 177.8º and bond length of C-O/N as 1.16 Å. These values were obtained by averaging distances and angles of terminal Fe-CO and Fe-CN^- ligands obtained from the 0.89 Å resolution structure of [NiFe]-hydrogenase (PDB: 4U9H).[10] These bond lengths and angles correspond well to those in the 1.39 Å resolution structure of CpI (PDB: 3C8Y), in which the CO and CN^- ligands were structurally assigned, [9] as well as those in the synthetic $[2Fe]_H$ mimic.[2] Strict restraints were defined for bond angles of Fe-C-O/N and bond lengths of C-O/N: σ (standard deviation) = 1º and 0.002 Å. The σ levels for bond lengths of Fe-CO/CN^- were then systematically defined for each individual refinement: σ = 0.02, 0.03, 0.04 and 0.05 Å. When a σ value of 0.01 Å was applied, the refined bond lengths (1.832-1.856 Å) were very close to the value in the input model (1.843 Å), indicating that the geometry restraints were too strong. Because of the presence of two copies in the asymmetric unit and the high occupancy of $[2Fe]_H$ and the extrinsic ligand at the open coordination site (OCS) in the CN^--treated crystal CpI-CN^--1 in both chains (1.00 and 0.98 in chain A and B, respectively; Table S3), the Fe-CO/CN^- bond lengths were averaged for each individual terminal Fe-CO/CN^- ligand from the two chains and the four different σ levels (Figure 1E). Each refinement was repeated at least twice with slightly different refinement strategies for other settings, such as turning the optimization of 'X-ray/stereochemistry' on or off or using another set of R-free flags. Because we observed slight deviations of Fe-C-O/N bond angles from those in the 1.39 Å resolution CpI structure PDB: 3C8Y [9], the σ level for Fe-C-O/N bond angles was additionally relaxed to 3º and all refinements were repeated. The refinements applying σ = 1° or 3° showed very consistent results (Table S4).

Infrared spectroscopy

Attenuated total reflection, Fourier transform infrared (ATR-FTIR) and IR absorption spectroscopy were used to collect spectra from protein solutions of HydA1 ([FeFe]-hydrogenase I from *Chlamydomonas reinhardtii*) and and CpI single crystals, respectively. A Bruker Tensor27 spectrometer placed in the anoxic glove box was used for the ATR-FITR experiments. Three aliquots of 1 mM as-isolated and *in vitro* maturated HydA1 protein were prepared. Aliquot 1 (without any additional treatment) was used as a control. The CN^- treatment was performed in the same way as for the CpI crystal diffraction experiment in that approximately 15 µl of the protein solutions were placed in the base of a sitting drop crystallization setup. After pipetting 500 µl of either 100 mM KCN or $K^{13}CN$ buffer into the reservoir, the setup was sealed immediately. The protein solution was then equilibrated with cyanide-containing buffer for 1-2 hours. Subsequently, 4 µl of the protein solution was pipetted onto the ZnSe/Si crystal and dried under N_2 purging for the ATR-FTIR experiment. IR spectra of CpI crystals were obtained in transmission, using a Bruker Hyperion 2000 IR microscope (with a Bruker Vertex 80v FTIR spectrometer providing IR source). The CpI crystals were distributed into an airtight sandwich cell (with CaF_2 windows) in the anoxic glove box. Spectra from both setups were collected at room temperature at a resolution of 2 cm^{-1}.

H_2 production assay

The H_2 production assay was done as previously described[5] but with the following modifications: due to the basic property of cyanide (pK_a of 9.2), the assay was done at pH 9.2 (200mM CHES (N-Cyclohexyl-2-aminoethanesulfonic acid)-NaOH). 100 mM NaDT and 10 mM methyl viologen were used as electron donor and mediator respectively. The amount of produced H_2 was quantified via gas chromatography.

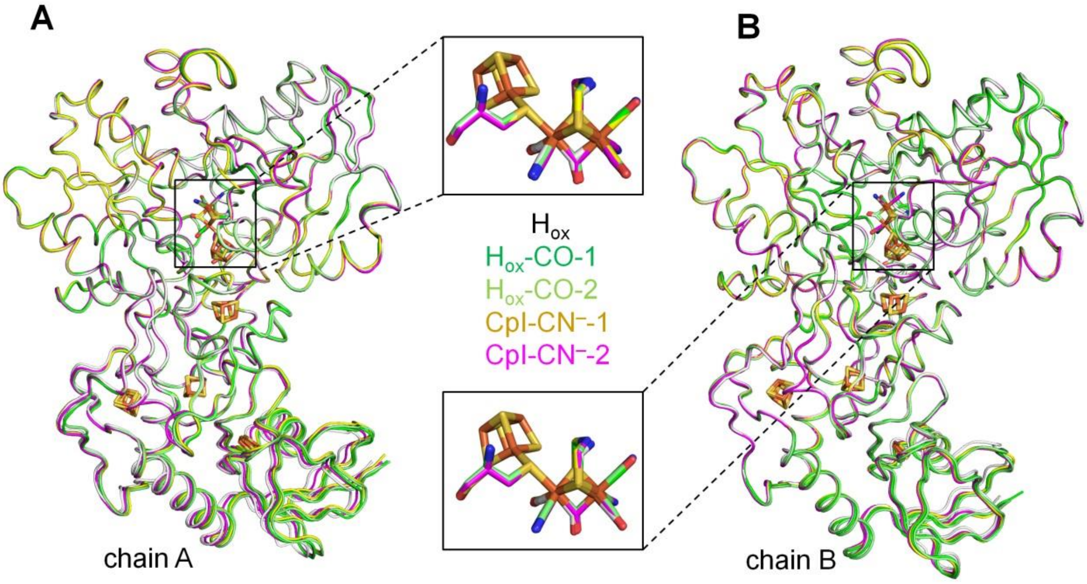

Figure S1. Superimposition of structures of CpI in various states. Structures obtained from treating crystals of CpI in the H_{ox} state with CO- and CN^{-} (H_{ox}-CO-1 (PDB: 8ALN), H_{ox}-CO-2 (PDB: 8AIO), CpI-CN^{-}-1 (PDB: 8AP2) and CpI-CN^{-}-2 (PDB: 8AJ6)) were aligned to CpI in the H_{ox} state (PDB: 4XDC).[4] A and B show superimpositions of all chains A and B, respectively, present in the asymmetric units. The insets show the overlays of the respective H-clusters drawn as sticks. Cartoons and the C atoms of the H-clusters are color-coded according to the crystal structure names shown in the middle.

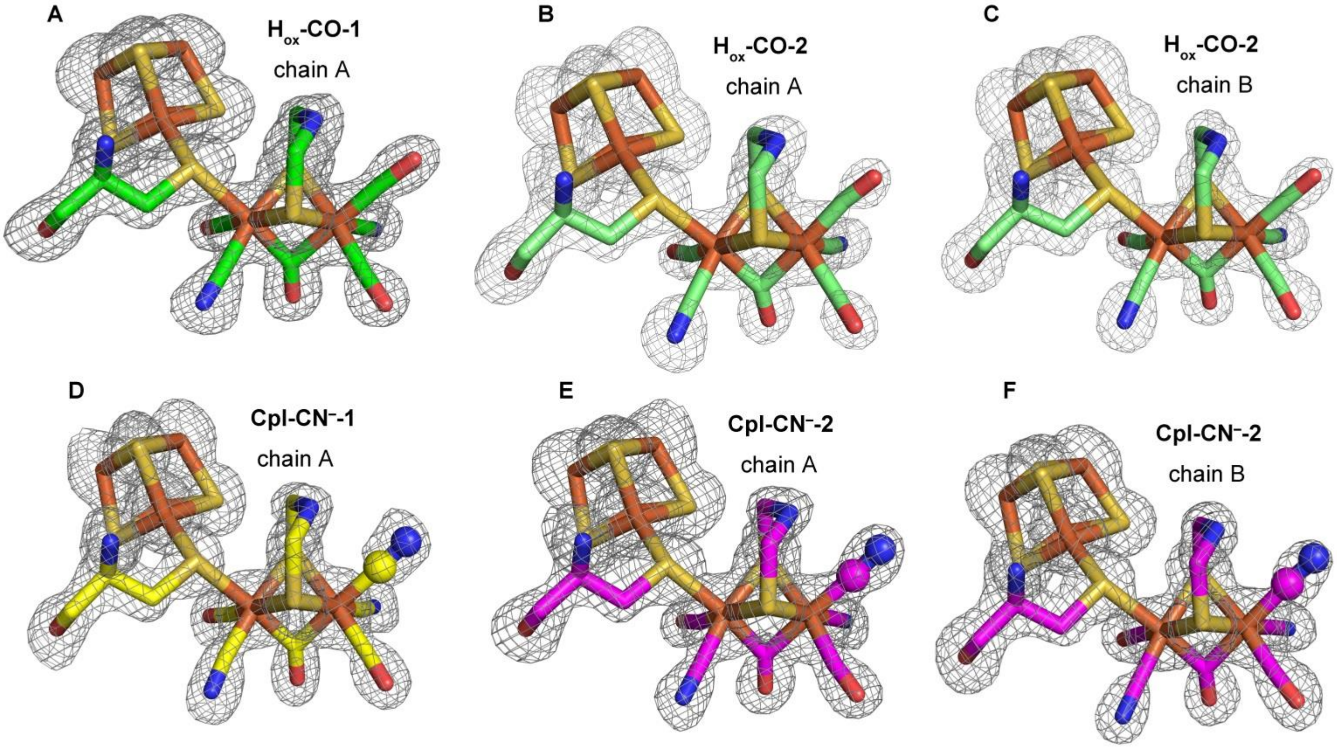

Figure S2. H-cluster structures of H_{ox}-CO-1, H_{ox}-CO-2, CpI-CN^{-}-1 and CpI-CN^{-}-2. The structures are differentiated by the colors of the carbon atoms: green and light green for H_{ox}-CO-1 (PDB: 8ALN) and H_{ox}-CO-2 (PDB: 8AIO), yellow and magenta for CpI-CN^{-}-1 (PDB: 8AP2) and CpI-CN^{-}-2 (PDB: 8AJ6), respectively. Letters A or B next to the names indicate one of the two chains in the asymmetric unit. Simulated annealing Fo-Fc maps were contoured at 3-4σ.

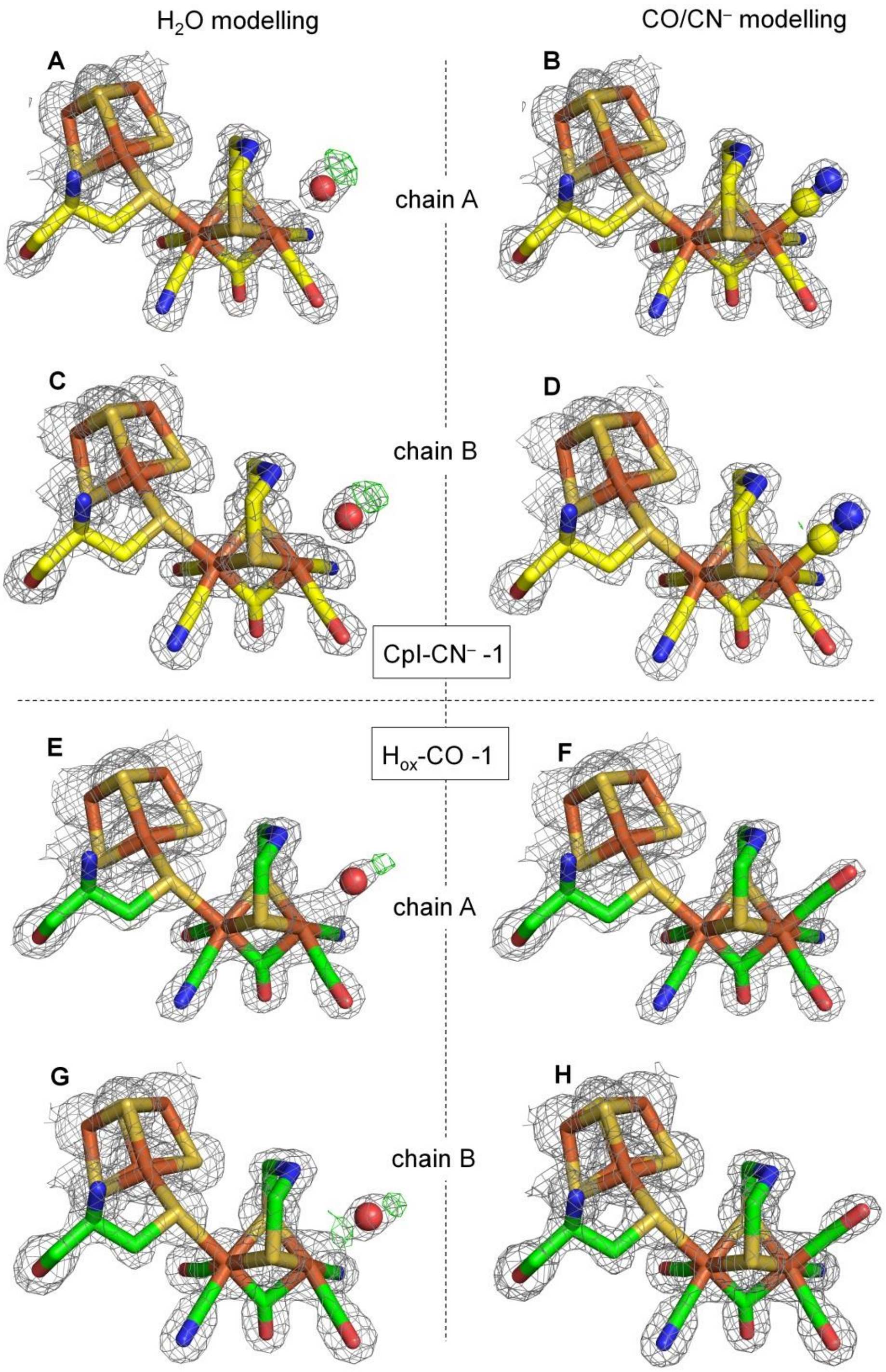

Figure S3. Comparison of modeling water molecules or CN^{-}/CO at the open coordination sites (OCS) of the H-clusters. (*2mFo-mFc*) and positive *mFo-mFc* maps were contoured at 2 σ and 5 to 6 σ, respectively. Carbon atoms of the two structures are colored yellow in CpI-CN^{-}-1 (PDB: 8AP2) and green in H_{ox}-CO-1 (PDB: 8ALN). **A, C, E** and **G** Modeling water molecules at the OCS resulted in strong unexplained positive electron densities (green mesh) in all structures. **B, D, F** and **H** Modeling with diatomic ligands diminished the unexplained electron densities.

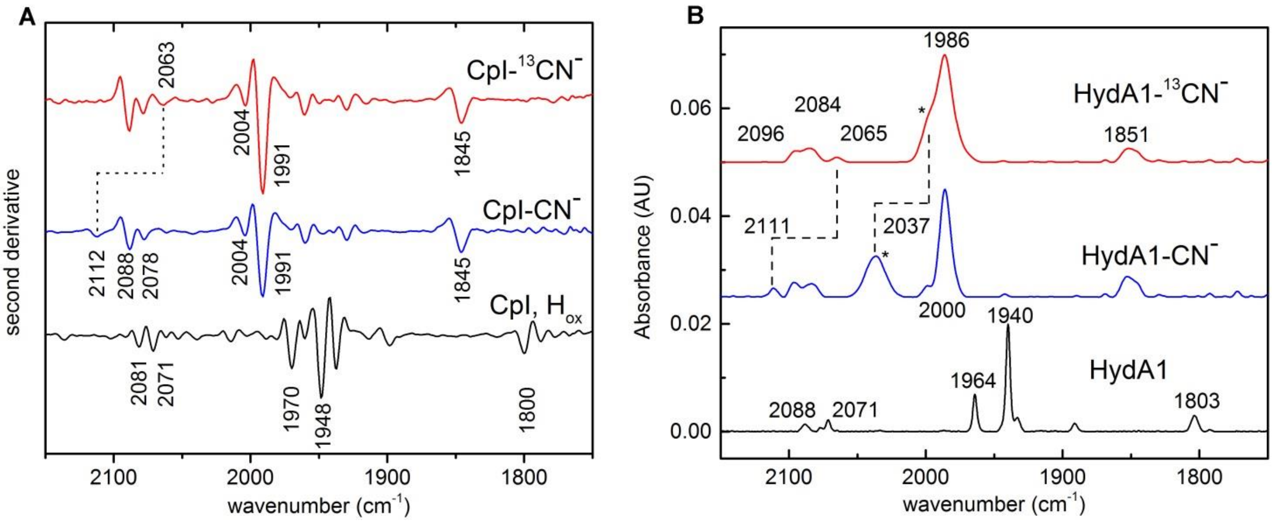

Figure S4. Infrared spectra of CpI single crystals and HydA1 protein films upon $^{12}CN^-$/$^{13}CN^-$ treatment. A IR absorption spectra of CpI single crystals under different conditions: as crystallized (H_{ox}, black line), KCN-treated (blue line) and $K^{13}CN$-treated (red line). Due to relatively low signal/noise ratios, the spectra are shown as second derivatives of the raw data. The cyanide treatment was done in the same way as for the diffraction experiment. Notably, the second terminal Fe-CO ligand at 2004 cm^{-1} has a low intensity. **B** ATR-FTIR spectra of dried HydA1 protein solutions under different conditions: as isolated (black line, without treatment), KCN treated (blue line) and $K^{13}CN$ treated (red line). Note that the broad peak at 2037 cm^{-1} (*) suggests the presence of ferrocyanide ($[Fe^{II}(CN)_6]^{4-}$), indicating some degradation of the H-cluster and a chelation of free Fe by CN^- anions. A significant shoulder at the peak 1986 cm^{-1} (*) suggests a similar red shift (by about 40 cm^{-1}) of the degradation product, and this shoulder potentially masks the low intensity of the second terminal CO ligand (at 2000 cm^{-1}). Similar degradation effects resulting from an excess of cyanide have been observed for carbon monoxide dehydrogenase.[11] However, the cyanide treatment did not induce any noticeable H-cluster degradation in CpI crystals, which fully agrees with the structural results.

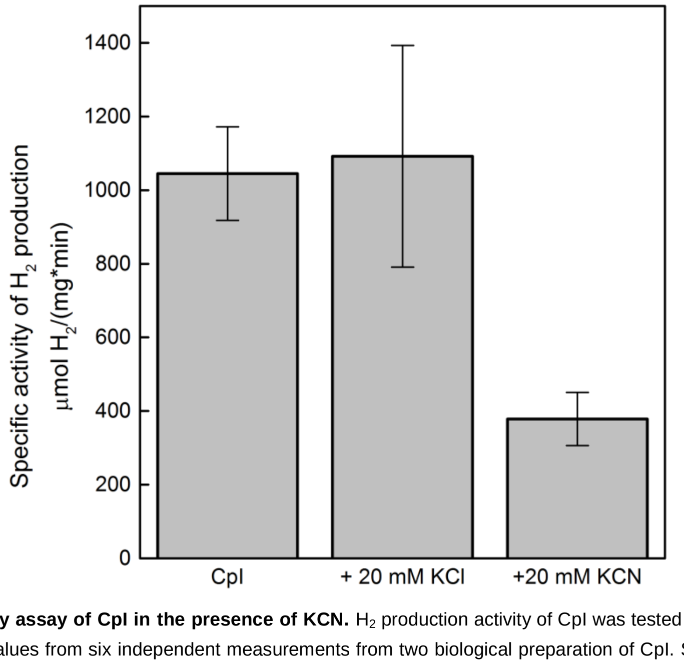

Figure S5. H_2 production activity assay of CpI in the presence of KCN. H_2 production activity of CpI was tested at pH 9.2 upon the addition of 20 mM KCl or KCN. The bars represent mean values from six independent measurements from two biological preparation of CpI. Standard deviations are represented by error bars.

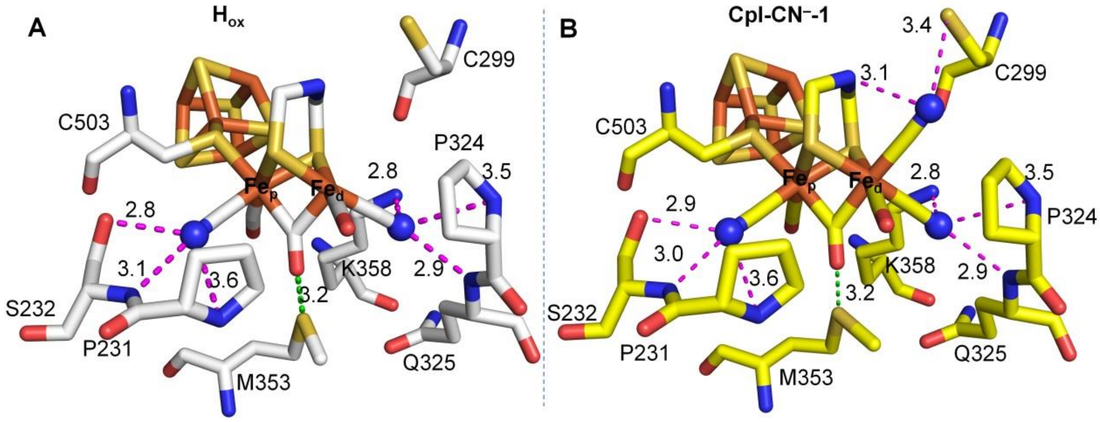

Figure S6. Polar interactions between the CN⁻/CO ligands and the protein environment. H-bond interactions that are formed between the protein environment and the ligands of the $[2Fe]_H$ subcluster[11] were compared in structures of CpI in the H_{ox} state (PDB: 4XDC)[4] and chain B of CpI-CN⁻-1 (PDB: 8AP2). Polar interactions are indicated by dashed lines, and the numbers show the distances in Å. For clarity, the second conformation of M353 in CpI-CN⁻-1 is not shown.

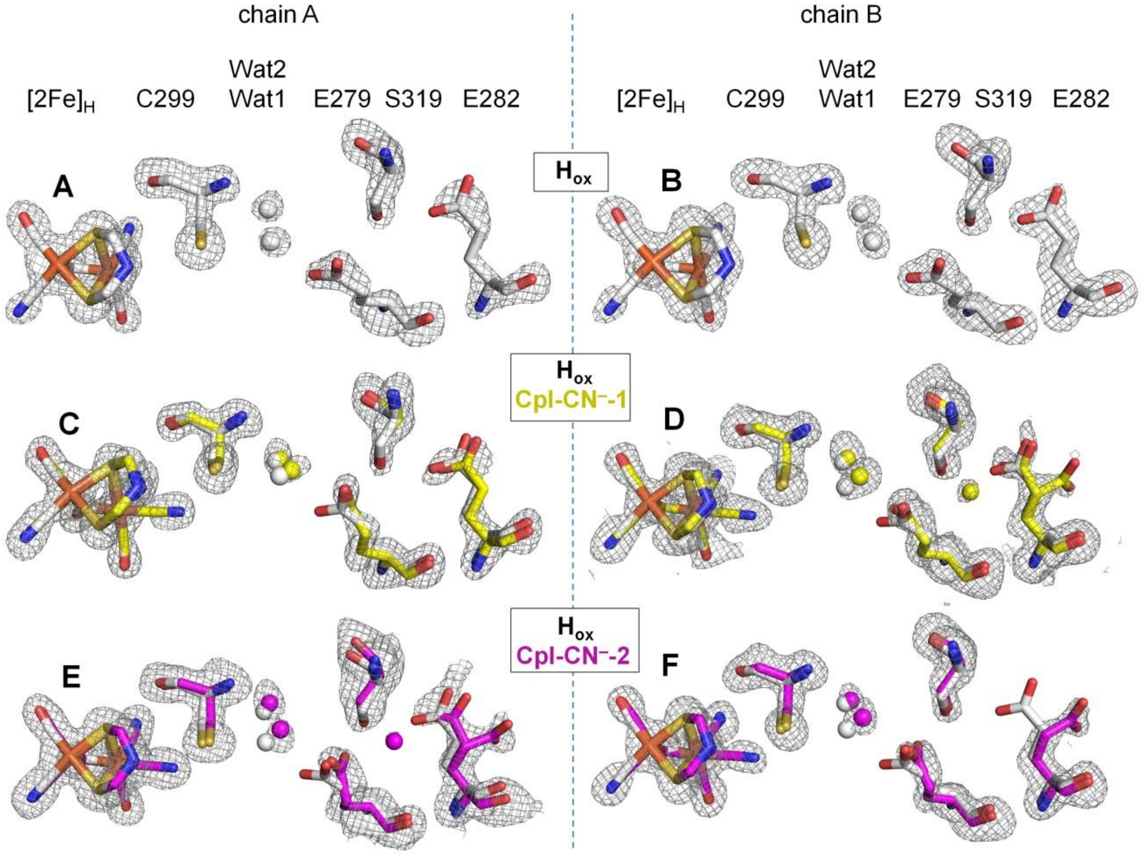

Figure S7. Structural comparison of elements of the proton transfer pathway (PTP) in CpI in the H_{ox}- and the CpI-CN⁻ states. A, B H-cluster and known PTP residues and water molecules of chain A (**A**) and chain B (**B**) of the asymmetric unit of the CpI H_{ox} crystal (PDB: 4XDC)[4]. **C, D, E, F** Superimposition of the PTP elements shown in **A** and **B** of the CN⁻-treated CpI crystals CpI-CN⁻-1 (PDB: 8AP2) (**C**, **D**) and CpI-CN⁻-2 (PDB: 8AJ6) (**E**, **F**) with those of the H_{ox} structure. Simulated annealing omitting maps (*Fo-Fc*) were contoured at 2-3σ.

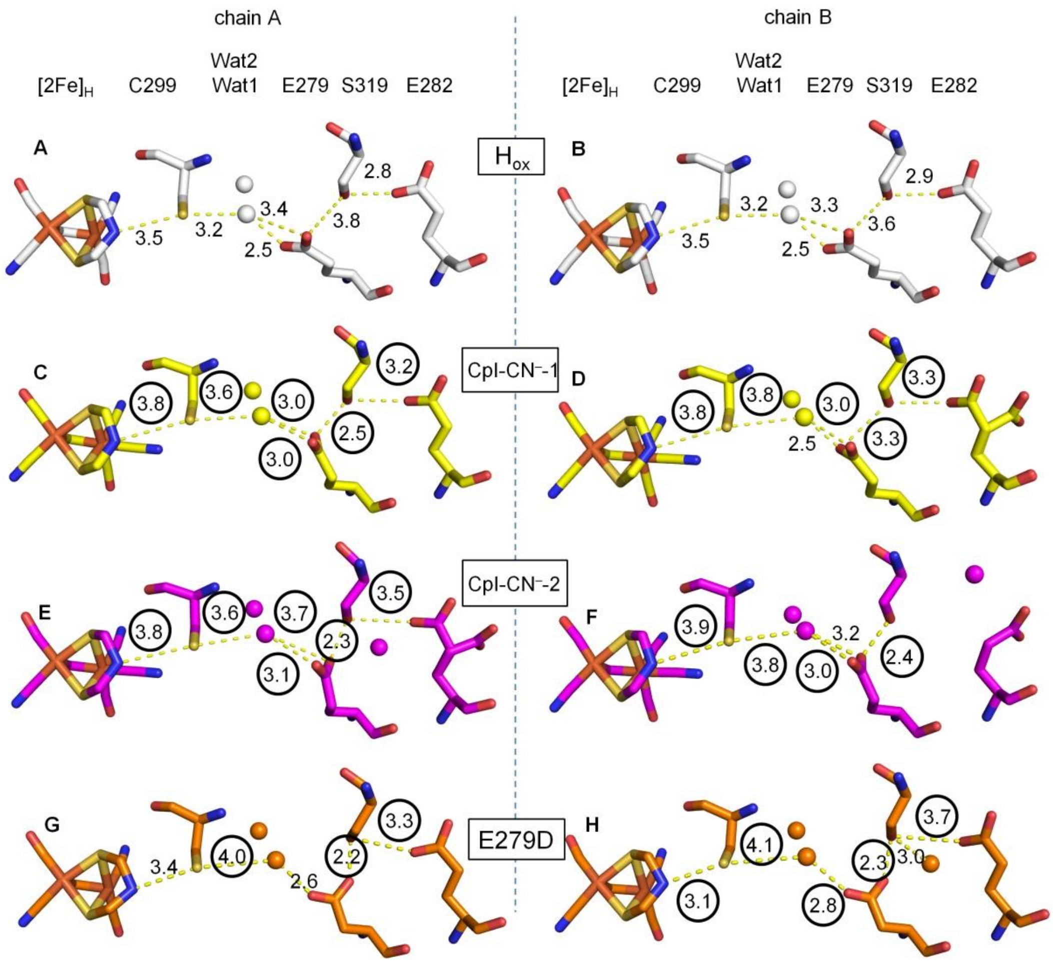

Figure S8. **Distances between H-bond partners of the proton transfer pathway (PTP) within structures H_{ox}, CpI-CN^{-}-1, CpI-CN^{-}-2 and CpI-E279D.** Distances that differ from those in the H_{ox} state (PDB: 4XDC)[4] (**A**, **B**) by at least 0.3 Å are highlighted with cycles. The structures are distinguished by coloring carbon atoms and water molecules white, yellow, magenta and orange for H_{ox} (PDB: 4XDC)[4] (**A**, **B**), CpI-CN^{-}-1 (PDB: 8AP2) (**C**, **D**), CpI-CN^{-}-2 (PDB: 8AJ6) (**E**, **F**), and E279D (PDB: 6YF4)[12] (**G**, **H**), respectively. Dashed lines indicate interactions between adjacent elements, and the numbers show the respective distances in Å.

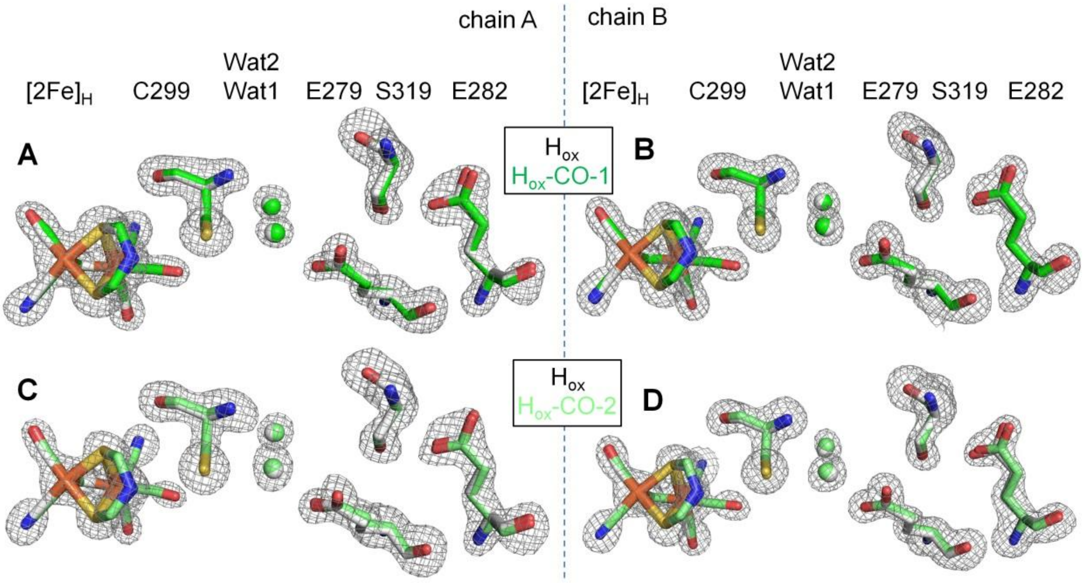

Figure S9. Proton transfer pathway (PTP) structures of CpI in the H_{ox}- and the H_{ox}-CO states. The PTP of each chain A and chain B of the asymmetric units of CpI H_{ox} crystals (PDB: 4XDC)[4] and of CO-treated crystals H_{ox}-CO-1 (PDB: 8ALN) (**A**, **B**) and H_{ox}-CO-2 (PDB: 8AIO) (**C**, **D**) were superimposed. Simulated annealing omitting maps (*Fo-Fc*) were contoured at 3σ.

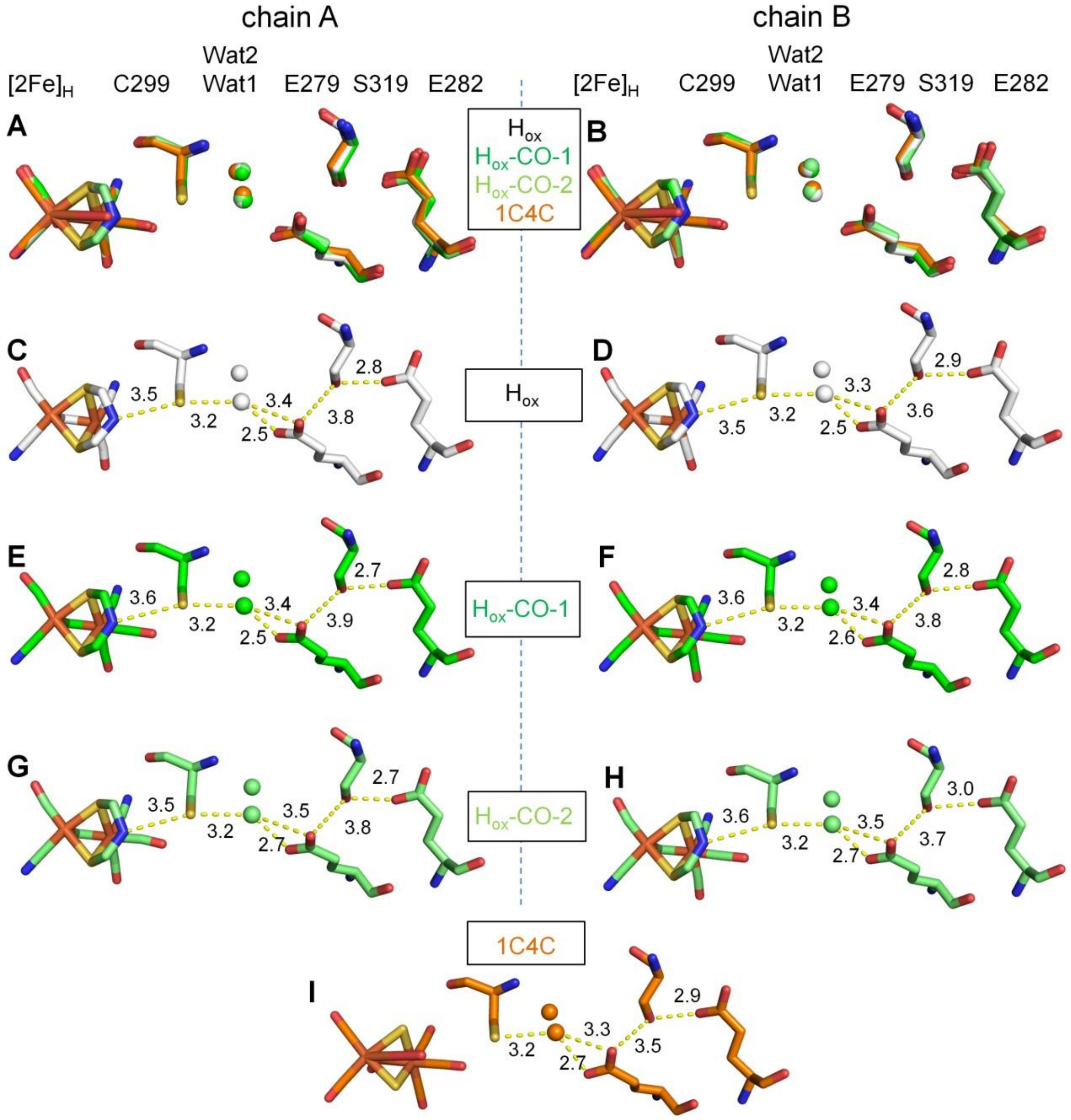

Figure S10. Comparison of proton transfer pathway (PTP) structures of CpI in the H_{ox}- and the H_{ox}-CO states. A, B Superimposition of the PTP of each chain A and chain B of CpI H_{ox} crystals (PDB: 4XDC)[4] and of CO-treated crystals H_{ox}-CO-1 (PDB: 8ALN) and H_{ox}-CO-2 (PDB: 8AIO). **C**-**I** Individual PTP structures in the indicated crystals in which the carbon atoms were colored white (H_{ox}), green (H_{ox}-CO-1), light green (H_{ox}-CO-2) and orange (H_{ox}-CO state from PDB: 1C4C[13]). Dashed lines indicate interactions whose distances in Å are indicated by the numbers. Note that the structure of the H-cluster was not clearly identified at the time of publication of structure 1C4C, and only one chain is present in the asymmetric unit.[13]

Table S1. Statistics for crystallographic data collection and refinement.

	H_{ox}-CO-1	H_{ox}-CO-2	CpI-CN$^-$-1	CpI-CN$^-$-2
**PDB**	8ALN	8AIO	8AP2	8AJ6
**Data collection**				
X-ray source	DESY-P14	X10SA-SLS	DESY-P13	ESRF-ID30B
Wavelength (Å)	0. 9763	0.7491	0.9724	0.9763
Space group	P 1 2_1 1	P 1 2_1 1	P 1 2_1 1	P 1 2_1 1
Cell dimensions				
a, b, c (Å)	89.86, 71.85, 103.23	87.60, 72.16, 103.21	88.07, 71.50, 102.78	90.18, 72.48, 103.71
α, β, γ (°)	90.00, 97.47, 90.00	90.00, 101.943, 90.00	90.00, 100.75, 90.00	90.00, 98.013, 90.00
Resolution (Å)	42.94-1.34 (1.388-1.34)[a]	40.03-1.52 (1.574-1.52)[a]	46.13-1.39 (1.44-1.39)[a]	47.47-1.50 (1.554-1.50)[a]
R_{merge}	0.06505 (2.289)[a]	0.09756 (1.639)[a]	0.1426 (2.423)[a]	0.1161 (1.702)[a]
I / σ(I)	12.40 (0.93)[a]	10.53 (1.07)[a]	8.64 (1.06)[a]	7.76 (1.12)[a]
Completeness (%)	98.61 (98.34)[a]	99.92 (99.89)	99.93 (99.87)[a]	99.82 (99.80)[a]
Redundancy	7.0 (7.2)[a]	7.0 (7.2)[a]	6.7 (6.4)[a]	6.3 (6.4)[a]
CC1/2	0.999 (0.591)[a]	0.999 (0.591)[a]	0.997 (0.487)[a]	0.997 (0.622)[a]
**Refinement**				
Resolution (Å)	42.94-1.34	40.03-1.52	46.13-1.39	47.47-1.50
No. reflections	287719	193291	251433	211263
R_{work} / R_{free}	0.1489/0.1750	0.1673/0.1922	0.1590 /0.1904	0.1752 /0.1987
No. atoms				
Protein	9029	8958	9108	9094
Ligand	135	144	133	127
Water	1136	827	1251	1172
B-factors				
Protein	30.81	29.91	24.72	28.90
Ligand	23.83	20.93	18.24	21.29
Water	40.37	37.94	37.48	37.13
R.m.s deviations				
Bond lengths (Å)	0.01	0.008	0.012	0.009
Bond angles (°)	1.00	0.87	1.09	0.99

[a] Numbers in brackets indicate values in the highest resolution shell.

Table S2. Calculated RMSD (root mean square deviations in Å) of the C-α atoms when superimposing the structures with 4XDC. All C-α atoms (572-579) were included in the calculations.

	H_{ox}-CO-1 (8ALN)	H_{ox}-CO-2 (8AIO)	CpI-CN$^-$-1 (8AP2)	CpI-CN$^-$-2 (8AJ6)
4XDC (A')[a]	0.612	0.736	0.686	0.435
4XDC (B')[b]	0.357	0.545	0.372	0.391

[a] and [b] indicate the values for the respective two chains A and B of the asymmetric unit.

Table S3. Group occupancies of the $[2Fe]_H$ moiety and extrinsic CO/CN$^-$ ligands. Group occupancies were calculated for each chain A and B in the asymmetric units. The extrinsic CO/CN$^-$ integration ratio was calculated by dividing the occupancies of the extrinsic ligands by the $[2Fe]_H$ occupancy.

	H_{ox}-CO-1 (8ALN)	H_{ox}-CO-2 (8AIO)	CpI-CN$^-$-1 (8AP2)	CpI-CN$^-$-2 (8AJ6)
$[2Fe]_H$ (A / B)[a]	0.91 / 0.93	0.82 / 0.80	1.00 / 0.98	0.98 / 0.99
Extrinsic CO/CN$^-$ (A / B)[a]	0.67 / 0.74	0.58 / 0.74	1.00 / 0.98	0.98 / 0.99
Extrinsic CO/CN$^-$ integration ratio (A /B)[a]	0.74 / 0.80	0.71 / 0.93	1.00 / 1.00	1.00 / 1.00

[a] A / B indicates values for the two copies (chain A and B) of the asymmetric units.

Table S4. Refined distances of Fe-CO/CN$^-$ modeled with different σ values of the Fe-C-O/N angle. Fe-CO/CN$^-$ were modeled in structure CpI-CN$^-$-1 as described in the materials and methods section setting the σ value of the Fe-C-O/N angle to 1 (A) or 3 degrees (B).

A

	Fe-C3	Fe-C4	Fe-C6	Fe-C7	Fe-C8
CN$^-$ modeling	1.799±0.012	1.892±0.013	1.911±0.027	1.800±0.018	1.894±0.022
CO modeling	1.784±0.012	1.880±0.008	1.891±0.018	1.777±0.021	1.868±0.006

B

	Fe-C3	Fe-C4	Fe-C6	Fe-C7	Fe-C8
CN$^-$ modeling	1.791±0.021	1.898±0.010	1.904±0.014	1.794±0.004	1.892±0.009
CO modeling	1.781±0.020	1.882±0.011	1.893±0.017	1.780±0.022	1.864±0.011

References

[1] J. M. Kuchenreuther, C. S. Grady-Smith, A. S. Bingham, S. J. George, S. P. Cramer, J. R. Swartz, *PLoS One* **2010**, 5 (11), e15491

[2] H. Li, T. B. Rauchfuss, *J. Am. Chem. Soc.* **2002**, 124 (5), 726-727.

[3] J. Esselborn, C. Lambertz, A. Adamska-Venkates, T. Simmons, G. Berggren, J. Noth, J. Siebel, A. Hemschemeier, V. Artero, E. Reijerse, M. Fontecave, W. Lubitz, T. Happe, *Nat. Chem. Biol.* **2013**, *9*, 607-609.

[4] J. Esselborn, N. Muraki, K. Klein, V. Engelbrecht, N. Metzler-Nolte, U.P. Apfel, E. Hofmann, G. Kurisu, T. Happe, *Chem. Sci.* **2016**, 7 (2), 959-968.

[5] J. Duan, M. Senger, J. Esselborn, V. Engelbrecht, F. Wittkamp, U. P. Apfel, E. Hofmann, S. T. Stripp, T. Happe, M. Winkler, *Nat. Commun.* **2018**, *9*, 4726.

[6] W. Kabsch, XDS. *Acta Crystallogr., Sect. D: Biol. Crystallogr.* **2010**, 66 (2), 125-132.

[7] P. D. Adams, P. V. Afonine, G. Bunkoczi, V. B.Chen, I. W. Davis, N. Echols, J. J. Headd, L. W. Hung, G. J. Kapral, R. W. Grosse-Kunstleve, *Acta Crystallogr., Sect. D: Biol. Crystallogr.* **2010**, 66 (2), 213-221.

[8] P. Emsley, K. Cowtan, *Acta Crystallogr., Sect. D: Biol. Crystallogr.* **2004**, 60 (12), 2126-2132.

[9] A. S. Pandey, T. V. Harris, L. J. Giles, J. W. Peters, R. K. Szilagyi, *J. Am. Chem. Soc.* **2008**, 130 (13), 4533-4540.

[10] H. Ogata, K. Nishikawa, W. Lubitz, *Nature* **2015**, 520 (7548), 571-574.

[11] A. Ciaccafava, D. Tombolelli, L. Domnik, J. Fesseler, J.-H. Jeoung, H. Dobbek, M. A. Mroginski, I. Zebger, P. Hildebrandt, *Chem. Sci.* **2016**, *7*, 3162-3171.

[12] O. Lampret, J. Duan, E. Hofmann, M. Winkler, F Armstrong, T. Happe, *Proc Natl. Acad. Sci. U. S. A.* **2020**, 117 (34), 20520-20529.

[13] B. J. Lemon, J. W. Peters, *Biochemistry* **1999**, 38 (40), 12969-12973.
