# Supplementary material for: Cyanide Binding to [FeFe]‐Hydrogenase Stabilizes the Alternative Configuration of the Proton Transfer Pathway
Source: Angew Chem Int Ed Engl. 2023 Jan 10;62(7):e202216903. doi: 10.1002/anie.202216903 (PMC10107461; doi:10.1002/anie.202216903)
Supplement: Supplementary file 2 — Supporting Information [file ANIE-62-0-s001.pdf]

Full wwPDB X-ray Structure Validation Report (i)

Jul 27, 2022 – 01:52 pm BST

PDB ID	:	8AIO
Title	:	CO-bound [FeFe]-hydrogenase I from Clostridium pasteurianum (CpI)
Deposited on	:	2022-07-26
Resolution	:	1.52 Å(reported)

This wwPDB validation report is for manuscript review

This is a Full wwPDB X-ray Structure Validation Report.

This report is produced by the wwPDB biocuration pipeline after annotation of the structure.

We welcome your comments at *validation@mail.wwpdb.org*
A user guide is available at
https://www.wwpdb.org/validation/2017/XrayValidationReportHelp
with specific help available everywhere you see the (i) symbol.

The types of validation reports are described at http://www.wwpdb.org/validation/2017/FAQs#types.

The following versions of software and data (see references (i)) were used in the production of this report:

MolProbity	:	4.02b-467
Mogul	:	1.8.4, CSD as541be (2020)
Xtriage (Phenix)	:	1.13
EDS	:	2.29
buster-report	:	1.1.7 (2018)
Percentile statistics	:	20191225.v01 (using entries in the PDB archive December 25th 2019)
Refmac	:	5.8.0267
CCP4	:	7.1.010 (Gargrove)
Ideal geometry (proteins)	:	Engh & Huber (2001)
Ideal geometry (DNA, RNA)	:	Parkinson et al. (1996)
Validation Pipeline (wwPDB-VP)	:	2.29

For Manuscript Review

1 Overall quality at a glance (i)

The following experimental techniques were used to determine the structure:
X-RAY DIFFRACTION

The reported resolution of this entry is 1.52 Å.

Percentile scores (ranging between 0-100) for global validation metrics of the entry are shown in the following graphic. The table shows the number of entries on which the scores are based.

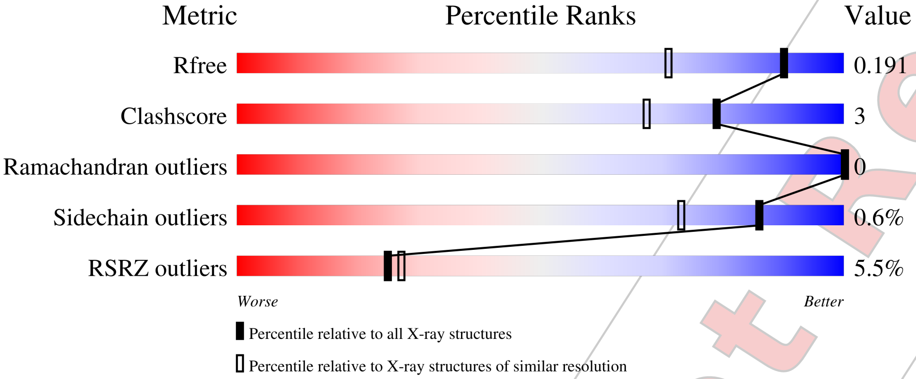

Metric	Whole archive (#Entries)	Similar resolution (#Entries, resolution range(Å))
R_{free}	130704	4009 (1.54-1.50)
Clashscore	141614	4249 (1.54-1.50)
Ramachandran outliers	138981	4148 (1.54-1.50)
Sidechain outliers	138945	4146 (1.54-1.50)
RSRZ outliers	127900	3943 (1.54-1.50)

The table below summarises the geometric issues observed across the polymeric chains and their fit to the electron density. The red, orange, yellow and green segments of the lower bar indicate the fraction of residues that contain outliers for >=3, 2, 1 and 0 types of geometric quality criteria respectively. A grey segment represents the fraction of residues that are not modelled. The numeric value for each fraction is indicated below the corresponding segment, with a dot representing fractions <=5% The upper red bar (where present) indicates the fraction of residues that have poor fit to the electron density. The numeric value is given above the bar.

Mol	Chain	Length	Quality of chain
1	A	584	3% / 93% 5% •
1	B	584	7% / 91% 7% •

For Manuscript Review

2 Entry composition (i)

There are 8 unique types of molecules in this entry. The entry contains 18777 atoms, of which 8864 are hydrogens and 0 are deuteriums.

In the tables below, the ZeroOcc column contains the number of atoms modelled with zero occupancy, the AltConf column contains the number of residues with at least one atom in alternate conformation and the Trace column contains the number of residues modelled with at most 2 atoms.

- Molecule 1 is a protein called Iron hydrogenase 1.

Mol	Chain	Residues	Atoms						ZeroOcc	AltConf	Trace
1	A	573	Total 8886	C 2800	H 4415	N 768	O 861	S 42	0	3	0
1	B	573	Total 8920	C 2809	H 4433	N 771	O 866	S 41	0	5	0

There are 20 discrepancies between the modelled and reference sequences:

Chain	Residue	Modelled	Actual	Comment	Reference
A	575	SER	-	expression tag	UNP P29166
A	576	ALA	-	expression tag	UNP P29166
A	577	TRP	-	expression tag	UNP P29166
A	578	SER	-	expression tag	UNP P29166
A	579	HIS	-	expression tag	UNP P29166
A	580	PRO	-	expression tag	UNP P29166
A	581	GLN	-	expression tag	UNP P29166
A	582	PHE	-	expression tag	UNP P29166
A	583	GLU	-	expression tag	UNP P29166
A	584	LYS	-	expression tag	UNP P29166
B	575	SER	-	expression tag	UNP P29166
B	576	ALA	-	expression tag	UNP P29166
B	577	TRP	-	expression tag	UNP P29166
B	578	SER	-	expression tag	UNP P29166
B	579	HIS	-	expression tag	UNP P29166
B	580	PRO	-	expression tag	UNP P29166
B	581	GLN	-	expression tag	UNP P29166
B	582	PHE	-	expression tag	UNP P29166
B	583	GLU	-	expression tag	UNP P29166
B	584	LYS	-	expression tag	UNP P29166

- Molecule 2 is 1,1,7-tris(hydroxymethyl)-8-oxidanylidene-2$l^{3},6$l^{3}-dithia-4-aza-1$l^{ 6},7$l^{5}-diferratricyclo[4.2.0.0^{2,7}]octane-1,7-dicarbonitrile (three-letter code: MHX) (formula: $C_8H_{14}Fe_2N_3O_4S_2$).

For Manuscript Review

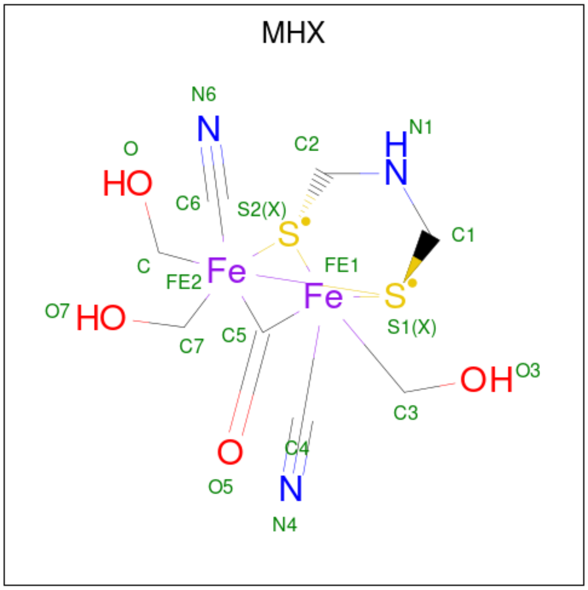

Mol	Chain	Residues	Atoms	ZeroOcc	AltConf
2	A	1	Total C Fe N O S<br>19 8 2 3 4 2	0	0
2	B	1	Total C Fe N O S<br>19 8 2 3 4 2	0	0

- Molecule 3 is IRON/SULFUR CLUSTER (three-letter code: SF4) (formula: Fe_4S_4) (labeled as "Ligand of Interest" by depositor).

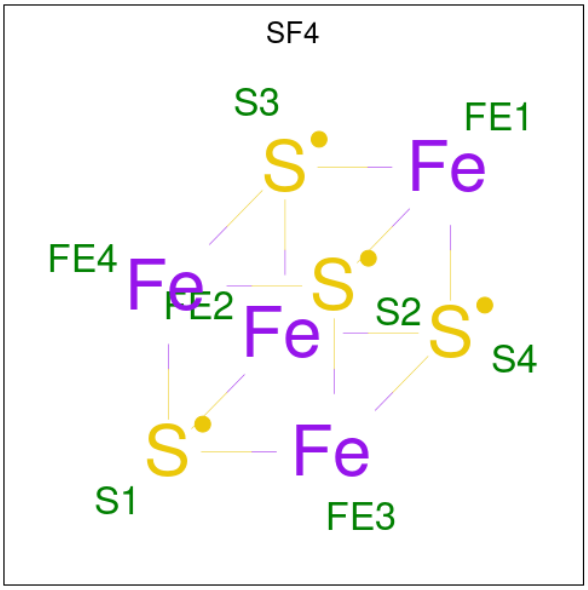

Mol	Chain	Residues	Atoms	ZeroOcc	AltConf
3	A	1	Total Fe S<br>8 4 4	0	0

Continued on next page...

For Manuscript Review

Continued from previous page...

Mol	Chain	Residues	Atoms	ZeroOcc	AltConf
3	A	1	Total Fe S<br>8 4 4	0	0
3	A	1	Total Fe S<br>8 4 4	0	0
3	A	1	Total Fe S<br>8 4 4	0	0
3	B	1	Total Fe S<br>8 4 4	0	0
3	B	1	Total Fe S<br>8 4 4	0	0
3	B	1	Total Fe S<br>8 4 4	0	0
3	B	1	Total Fe S<br>8 4 4	0	0

- Molecule 4 is FE2/S2 (INORGANIC) CLUSTER (three-letter code: FES) (formula: Fe_2S_2) (labeled as "Ligand of Interest" by depositor).

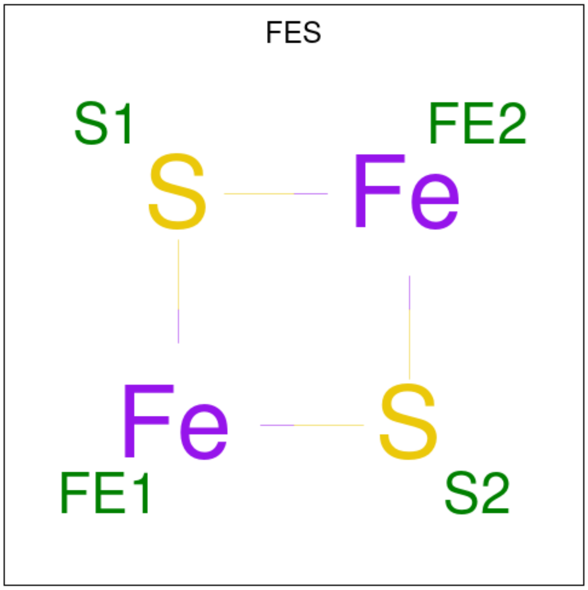

Mol	Chain	Residues	Atoms	ZeroOcc	AltConf
4	A	1	Total Fe S<br>4 2 2	0	0
4	B	1	Total Fe S<br>4 2 2	0	0

- Molecule 5 is GLYCEROL (three-letter code: GOL) (formula: $C_3H_8O_3$).

For Manuscript Review

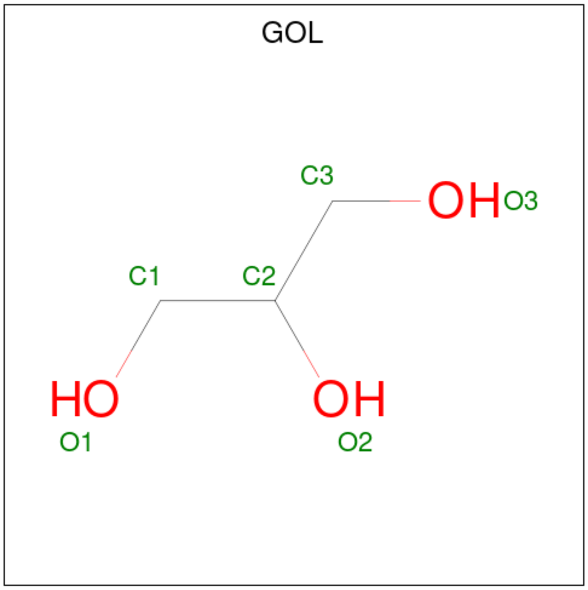

Mol	Chain	Residues	Atoms	ZeroOcc	AltConf
5	A	1	Total C H O<br>14 3 8 3	0	0
5	B	1	Total C H O<br>14 3 8 3	0	0

- Molecule 6 is MAGNESIUM ION (three-letter code: MG) (formula: Mg).

Mol	Chain	Residues	Atoms	ZeroOcc	AltConf
6	A	2	Total Mg<br>2 2	0	0
6	B	2	Total Mg<br>2 2	0	0

- Molecule 7 is CHLORIDE ION (three-letter code: CL) (formula: Cl).

Mol	Chain	Residues	Atoms	ZeroOcc	AltConf
7	A	1	Total Cl<br>1 1	0	0
7	B	1	Total Cl<br>1 1	0	0

- Molecule 8 is water.

Mol	Chain	Residues	Atoms	ZeroOcc	AltConf
8	A	411	Total O<br>411 411	0	0

Continued on next page...

For Manuscript Review

Continued from previous page...

Mol	Chain	Residues	Atoms		ZeroOcc	AltConf
			Total	O		
8	B	416	416	416	0	0

For Manuscript Review

3 Residue-property plots (i)

These plots are drawn for all protein, RNA, DNA and oligosaccharide chains in the entry. The first graphic for a chain summarises the proportions of the various outlier classes displayed in the second graphic. The second graphic shows the sequence view annotated by issues in geometry and electron density. Residues are color-coded according to the number of geometric quality criteria for which they contain at least one outlier: green = 0, yellow = 1, orange = 2 and red = 3 or more. A red dot above a residue indicates a poor fit to the electron density (RSRZ > 2). Stretches of 2 or more consecutive residues without any outlier are shown as a green connector. Residues present in the sample, but not in the model, are shown in grey.

- Molecule 1: Iron hydrogenase 1

Chain A:

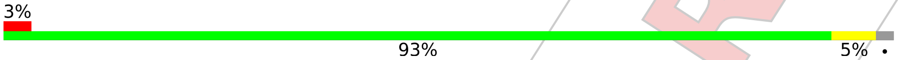

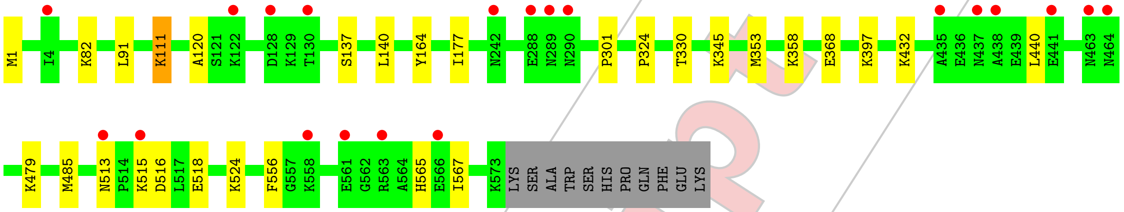

- Molecule 1: Iron hydrogenase 1

Chain B:

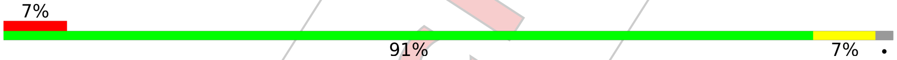

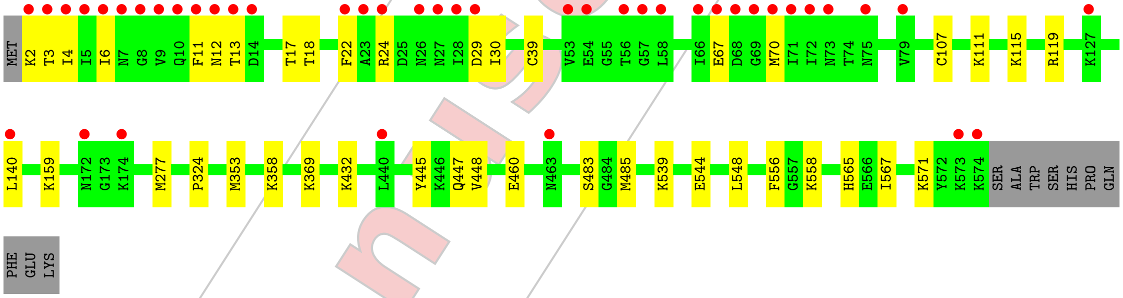

For Manuscript Review

4 Data and refinement statistics (i)

Property	Value	Source
Space group	P 1 21 1	Depositor
Cell constants<br>a, b, c, α, β, γ	87.60Å 72.16Å 103.21Å<br>90.00° 101.94° 90.00°	Depositor
Resolution (Å)	40.03 – 1.52<br>48.07 – 1.52	Depositor<br>EDS
% Data completeness<br>(in resolution range)	99.9 (40.03-1.52)<br>100.0 (48.07-1.52)	Depositor<br>EDS
R_{merge}	0.10	Depositor
R_{sym}	(Not available)	Depositor
$< I/\sigma(I) >$ [1]	1.32 (at 1.52Å)	Xtriage
Refinement program	PHENIX 1.19.1_4122	Depositor
R, R_{free}	0.167 , 0.192<br>0.167 , 0.191	Depositor<br>DCC
R_{free} test set	9665 reflections (5.00%)	wwPDB-VP
Wilson B-factor (Å^2)	19.0	Xtriage
Anisotropy	0.477	Xtriage
Bulk solvent k_{sol}(e/Å^3), B_{sol}(Å^2)	(Not available) , (Not available)	EDS
L-test for twinning[2]	$< \|L\| > = 0.50$, $< L^2 > = 0.33$	Xtriage
Estimated twinning fraction	No twinning to report.	Xtriage
F_o,F_c correlation	0.98	EDS
Total number of atoms	18777	wwPDB-VP
Average B, all atoms (Å^2)	33.0	wwPDB-VP

Xtriage's analysis on translational NCS is as follows: *The largest off-origin peak in the Patterson function is 7.19% of the height of the origin peak. No significant pseudotranslation is detected.*

[1]Intensities estimated from amplitudes.

[2]Theoretical values of $< |L| >$, $< L^2 >$ for acentric reflections are 0.5, 0.333 respectively for untwinned datasets, and 0.375, 0.2 for perfectly twinned datasets.

For Manuscript Review

5 Model quality (i)

5.1 Standard geometry (i)

Bond lengths and bond angles in the following residue types are not validated in this section: FES, MG, SF4, CL, MHX, GOL

The Z score for a bond length (or angle) is the number of standard deviations the observed value is removed from the expected value. A bond length (or angle) with $|Z| > 5$ is considered an outlier worth inspection. RMSZ is the root-mean-square of all Z scores of the bond lengths (or angles).

Mol	Chain	Bond lengths		Bond angles	
		RMSZ	#$|Z|$ >5	RMSZ	#$|Z|$ >5
1	A	0.38	0/4544	0.59	0/6119
1	B	0.38	0/4560	0.60	0/6140
All	All	0.38	0/9104	0.59	0/12259

There are no bond length outliers.

There are no bond angle outliers.

There are no chirality outliers.

There are no planarity outliers.

5.2 Too-close contacts (i)

In the following table, the Non-H and H(model) columns list the number of non-hydrogen atoms and hydrogen atoms in the chain respectively. The H(added) column lists the number of hydrogen atoms added and optimized by MolProbity. The Clashes column lists the number of clashes within the asymmetric unit, whereas Symm-Clashes lists symmetry-related clashes.

Mol	Chain	Non-H	H(model)	H(added)	Clashes	Symm-Clashes
1	A	4471	4415	4434	22	0
1	B	4487	4433	4444	27	0
2	A	19	0	0	2	0
2	B	19	0	0	1	0
3	A	32	0	0	1	0
3	B	32	0	0	1	0
4	A	4	0	0	0	0
4	B	4	0	0	0	0
5	A	6	8	8	0	0
5	B	6	8	8	0	0
6	A	2	0	0	0	0

Continued on next page...

For Manuscript Review

Continued from previous page...

Mol	Chain	Non-H	H(model)	H(added)	Clashes	Symm-Clashes
6	B	2	0	0	0	0
7	A	1	0	0	0	0
7	B	1	0	0	0	0
8	A	411	0	0	7	1
8	B	416	0	0	1	1
All	All	9913	8864	8894	50	1

The all-atom clashscore is defined as the number of clashes found per 1000 atoms (including hydrogen atoms). The all-atom clashscore for this structure is 3.

All (50) close contacts within the same asymmetric unit are listed below, sorted by their clash magnitude.

Atom-1	Atom-2	Interatomic distance (Å)	Clash overlap (Å)
1:B:39[A]:CYS:SG	1:B:159:LYS:NZ	2.56	0.78
1:A:432:LYS:HE3	1:A:440:LEU:HD13	1.70	0.73
1:B:353[B]:MET:HE2	1:B:358:LYS:HG3	1.78	0.65
1:B:565:HIS:ND1	8:B:702:HOH:O	2.30	0.64
1:B:29:ASP:OD2	1:B:115:LYS:NZ	2.29	0.62
1:B:107:CYS:O	1:B:111:LYS:HD3	1.99	0.62
1:A:513:ASN:HD21	1:A:515:LYS:HE3	1.65	0.62
1:B:556:PHE:HE1	1:B:567:ILE:HD11	1.66	0.59
1:B:111:LYS:HD2	1:B:111:LYS:N	2.18	0.59
1:A:556:PHE:HE1	1:A:567:ILE:HD11	1.69	0.58
1:A:513:ASN:OD1	1:A:515:LYS:HB2	2.09	0.53
1:B:2:LYS:N	1:B:13:THR:O	2.42	0.53
1:A:432:LYS:HE3	1:A:440:LEU:CD1	2.38	0.52
1:A:111:LYS:NZ	8:A:709:HOH:O	2.42	0.52
2:A:601:MHX:C	2:A:601:MHX:N1	2.73	0.52
1:B:324:PRO:HG2	2:B:601:MHX:C	2.39	0.51
1:A:515:LYS:O	1:A:518:GLU:HG2	2.10	0.51
1:B:24:ARG:NH2	1:B:30:ILE:O	2.41	0.51
1:A:324:PRO:HG2	2:A:601:MHX:C	2.42	0.50
1:B:4:ILE:HD11	1:B:67:GLU:O	2.11	0.50
1:A:111:LYS:HE3	8:A:871:HOH:O	2.11	0.49
1:B:483:SER:OG	1:B:485:MET:HG3	2.13	0.48
1:B:67:GLU:O	1:B:70:MET:HG3	2.12	0.48
1:A:368:GLU:OE1	8:A:701:HOH:O	2.20	0.48
1:A:330:THR:HG22	1:A:567:ILE:HD12	1.95	0.47
1:A:524:LYS:NZ	8:A:711:HOH:O	2.42	0.47
1:B:277:MET:HG3	1:B:548:LEU:HD21	1.97	0.47
1:B:140:LEU:HD11	3:B:603:SF4:S2	2.56	0.46

Continued on next page...

For Manuscript Review

Continued from previous page...

Atom-1	Atom-2	Interatomic distance (Å)	Clash overlap (Å)
1:A:516:ASP:OD1	8:A:702:HOH:O	2.21	0.46
1:B:3:THR:HG22	1:B:12:ASN:OD1	2.14	0.46
1:A:397:LYS:HA	1:A:397:LYS:HD2	1.70	0.46
1:B:3:THR:HA	1:B:11:PHE:O	2.16	0.44
1:B:4:ILE:O	1:B:11:PHE:N	2.43	0.44
1:B:4:ILE:HD12	1:B:70:MET:HB2	1.99	0.44
1:B:447:GLN:H	1:B:447:GLN:CD	2.21	0.44
1:A:353[B]:MET:HE2	1:A:358:LYS:HG3	1.99	0.44
1:A:479:LYS:HE2	1:A:485:MET:HE1	2.01	0.43
1:A:565:HIS:HE1	8:A:746:HOH:O	2.03	0.42
1:B:369:LYS:HE3	1:B:567:ILE:HG22	2.00	0.42
1:B:445:TYR:O	1:B:448:VAL:HG22	2.21	0.41
1:B:539:LYS:HD3	1:B:544:GLU:O	2.19	0.41
1:B:558:LYS:HB3	1:B:558:LYS:HE3	1.76	0.41
1:A:91:LEU:HB3	1:A:120:ALA:HB2	2.03	0.41
1:A:137:SER:HB3	1:A:140:LEU:O	2.21	0.41
1:A:164:TYR:HA	8:A:735:HOH:O	2.20	0.41
1:A:301:PRO:HD2	3:A:602:SF4:S2	2.61	0.41
1:B:432:LYS:HE2	1:B:460:GLU:O	2.21	0.40
1:B:6:ILE:HD12	1:B:22:PHE:HE2	1.86	0.40
1:A:177:ILE:O	1:A:177:ILE:HD12	2.22	0.40
1:B:17:THR:HG23	1:B:18:THR:O	2.21	0.40

All (1) symmetry-related close contacts are listed below. The label for Atom-2 includes the symmetry operator and encoded unit-cell translations to be applied.

Atom-1	Atom-2	Interatomic distance (Å)	Clash overlap (Å)
8:A:1054:HOH:O	8:B:727:HOH:O[1_556]	2.11	0.09

5.3 Torsion angles (i)

5.3.1 Protein backbone (i)

In the following table, the Percentiles column shows the percent Ramachandran outliers of the chain as a percentile score with respect to all X-ray entries followed by that with respect to entries of similar resolution.

The Analysed column shows the number of residues for which the backbone conformation was analysed, and the total number of residues.

For Manuscript Review

Mol	Chain	Analysed	Favoured	Allowed	Outliers	Percentiles	
1	A	574/584 (98%)	558 (97%)	16 (3%)	0	100	100
1	B	576/584 (99%)	554 (96%)	22 (4%)	0	100	100
All	All	1150/1168 (98%)	1112 (97%)	38 (3%)	0	100	100

There are no Ramachandran outliers to report.

5.3.2 Protein sidechains (i)

In the following table, the Percentiles column shows the percent sidechain outliers of the chain as a percentile score with respect to all X-ray entries followed by that with respect to entries of similar resolution.

The Analysed column shows the number of residues for which the sidechain conformation was analysed, and the total number of residues.

Mol	Chain	Analysed	Rotameric	Outliers	Percentiles	
1	A	492/499 (99%)	488 (99%)	4 (1%)	81	65
1	B	494/499 (99%)	492 (100%)	2 (0%)	91	82
All	All	986/998 (99%)	980 (99%)	6 (1%)	86	73

All (6) residues with a non-rotameric sidechain are listed below:

Mol	Chain	Res	Type
1	A	1	MET
1	A	82	LYS
1	A	111	LYS
1	A	345	LYS
1	B	119	ARG
1	B	571	LYS

Sometimes sidechains can be flipped to improve hydrogen bonding and reduce clashes. There are no such sidechains identified.

5.3.3 RNA (i)

There are no RNA molecules in this entry.

5.4 Non-standard residues in protein, DNA, RNA chains (i)

There are no non-standard protein/DNA/RNA residues in this entry.

For Manuscript Review

5.5 Carbohydrates (i)

There are no monosaccharides in this entry.

5.6 Ligand geometry (i)

Of 20 ligands modelled in this entry, 6 are monoatomic - leaving 14 for Mogul analysis.

In the following table, the Counts columns list the number of bonds (or angles) for which Mogul statistics could be retrieved, the number of bonds (or angles) that are observed in the model and the number of bonds (or angles) that are defined in the Chemical Component Dictionary. The Link column lists molecule types, if any, to which the group is linked. The Z score for a bond length (or angle) is the number of standard deviations the observed value is removed from the expected value. A bond length (or angle) with $|Z| > 2$ is considered an outlier worth inspection. RMSZ is the root-mean-square of all Z scores of the bond lengths (or angles).

Mol	Type	Chain	Res	Link	Bond lengths			Bond angles		
					Counts	RMSZ	#$\lvert Z \rvert > 2$	Counts	RMSZ	#$\lvert Z \rvert > 2$
3	SF4	B	604	1	0,12,12	-	-	-		
3	SF4	A	603	1	0,12,12	-	-	-		
5	GOL	A	607	-	5,5,5	1.14	0	5,5,5	0.76	0
4	FES	A	606	1	0,4,4	-	-	-		
3	SF4	B	603	1	0,12,12	-	-	-		
5	GOL	B	608	-	5,5,5	1.01	0	5,5,5	0.92	0
4	FES	B	606	1	0,4,4	-	-	-		
3	SF4	B	605	1	0,12,12	-	-	-		
3	SF4	A	605	1	0,12,12	-	-	-		
2	MHX	B	601	1	11,21,21	4.29	7 (63%)	2,42,42	2.51	1 (50%)
3	SF4	B	602	1	0,12,12	-	-	-		
3	SF4	A	602	1	0,12,12	-	-	-		
3	SF4	A	604	1	0,12,12	-	-	-		
2	MHX	A	601	1	11,21,21	4.13	6 (54%)	2,42,42	2.73	1 (50%)

In the following table, the Chirals column lists the number of chiral outliers, the number of chiral centers analysed, the number of these observed in the model and the number defined in the Chemical Component Dictionary. Similar counts are reported in the Torsion and Rings columns. '-' means no outliers of that kind were identified.

Mol	Type	Chain	Res	Link	Chirals	Torsions	Rings
3	SF4	B	604	1	-	-	0/6/5/5
3	SF4	A	603	1	-	-	0/6/5/5
5	GOL	A	607	-	-	0/4/4/4	-
5	GOL	B	608	-	-	0/4/4/4	-
3	SF4	B	603	1	-	-	0/6/5/5

Continued on next page...

For Manuscript Review

Continued from previous page...

Mol	Type	Chain	Res	Link	Chirals	Torsions	Rings
4	FES	A	606	1	-	-	0/1/1/1
4	FES	B	606	1	-	-	0/1/1/1
3	SF4	B	605	1	-	-	0/6/5/5
3	SF4	A	605	1	-	-	0/6/5/5
2	MHX	B	601	1	-	-	0/5/3/3
3	SF4	B	602	1	-	-	0/6/5/5
3	SF4	A	602	1	-	-	0/6/5/5
3	SF4	A	604	1	-	-	0/6/5/5
2	MHX	A	601	1	-	-	0/5/3/3

All (13) bond length outliers are listed below:

Mol	Chain	Res	Type	Atoms	Z	Observed(Å)	Ideal(Å)
2	A	601	MHX	O-C	-7.93	1.15	1.40
2	B	601	MHX	O-C	-7.76	1.16	1.40
2	A	601	MHX	O7-C7	-7.09	1.18	1.40
2	B	601	MHX	O7-C7	-7.03	1.18	1.40
2	A	601	MHX	O3-C3	-5.56	1.23	1.40
2	B	601	MHX	O3-C3	-5.40	1.23	1.40
2	B	601	MHX	S2-FE1	4.72	2.33	2.26
2	A	601	MHX	C4-N4	4.45	1.23	1.15
2	B	601	MHX	C4-N4	4.21	1.22	1.15
2	B	601	MHX	S1-FE1	3.91	2.31	2.26
2	A	601	MHX	S1-FE1	3.51	2.31	2.26
2	A	601	MHX	C2-S2	2.56	1.89	1.85
2	B	601	MHX	C2-S2	2.41	1.89	1.85

All (2) bond angle outliers are listed below:

Mol	Chain	Res	Type	Atoms	Z	Observed(^{o})	Ideal(^{o})
2	A	601	MHX	S2-C2-N1	-3.55	107.40	117.18
2	B	601	MHX	S2-C2-N1	-3.39	107.84	117.18

There are no chirality outliers.

There are no torsion outliers.

There are no ring outliers.

4 monomers are involved in 5 short contacts:

Mol	Chain	Res	Type	Clashes	Symm-Clashes
3	B	603	SF4	1	0

Continued on next page...

For Manuscript Review

Continued from previous page...

Mol	Chain	Res	Type	Clashes	Symm-Clashes
2	B	601	MHX	1	0
3	A	602	SF4	1	0
2	A	601	MHX	2	0

The following is a two-dimensional graphical depiction of Mogul quality analysis of bond lengths, bond angles, torsion angles, and ring geometry for all instances of the Ligand of Interest. In addition, ligands with molecular weight > 250 and outliers as shown on the validation Tables will also be included. For torsion angles, if less then 5% of the Mogul distribution of torsion angles is within 10 degrees of the torsion angle in question, then that torsion angle is considered an outlier. Any bond that is central to one or more torsion angles identified as an outlier by Mogul will be highlighted in the graph. For rings, the root-mean-square deviation (RMSD) between the ring in question and similar rings identified by Mogul is calculated over all ring torsion angles. If the average RMSD is greater than 60 degrees and the minimal RMSD between the ring in question and any Mogul-identified rings is also greater than 60 degrees, then that ring is considered an outlier. The outliers are highlighted in purple. The color gray indicates Mogul did not find sufficient equivalents in the CSD to analyse the geometry.

Ligand SF4 B 604

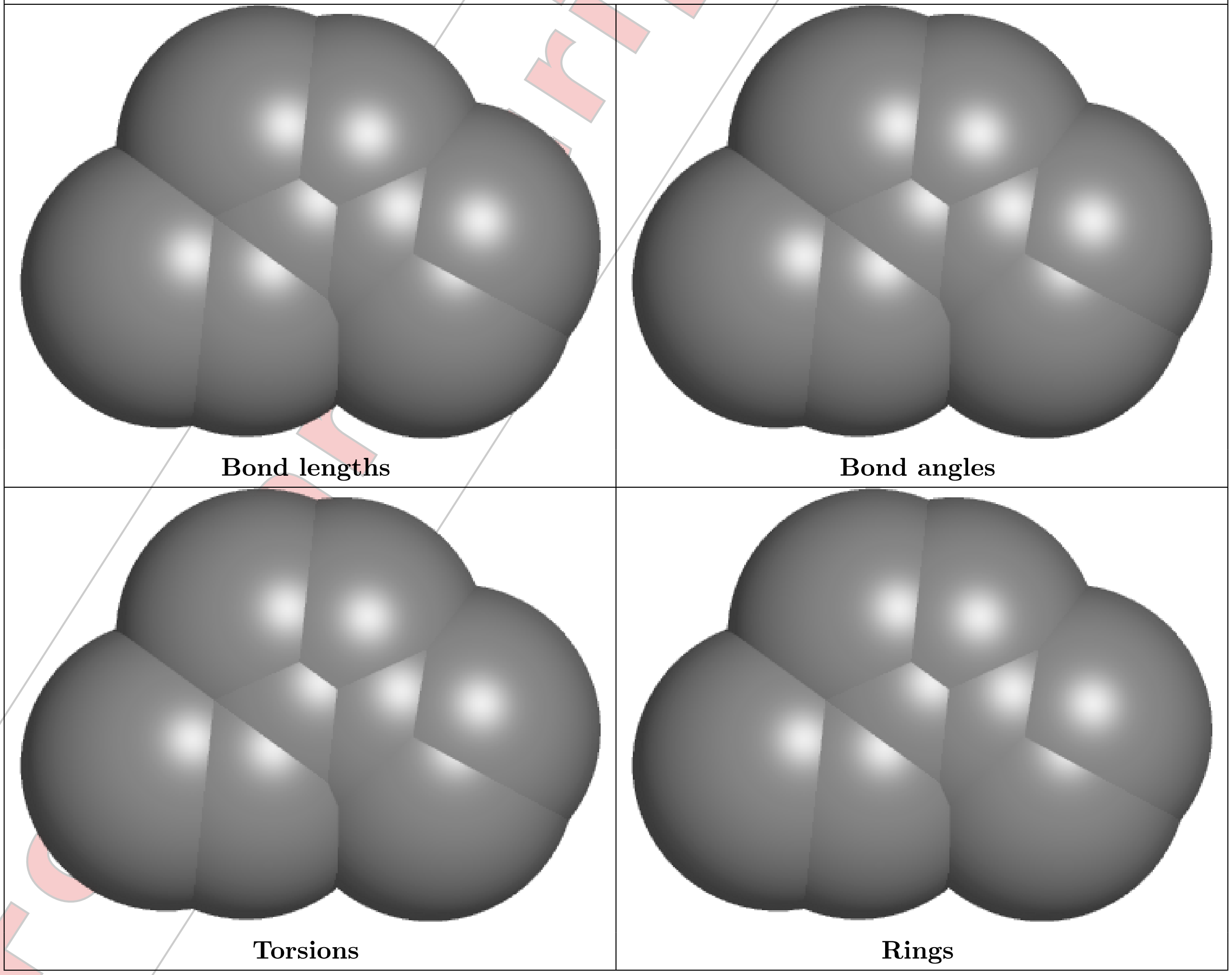

For Manuscript Review

Ligand SF4 A 603	
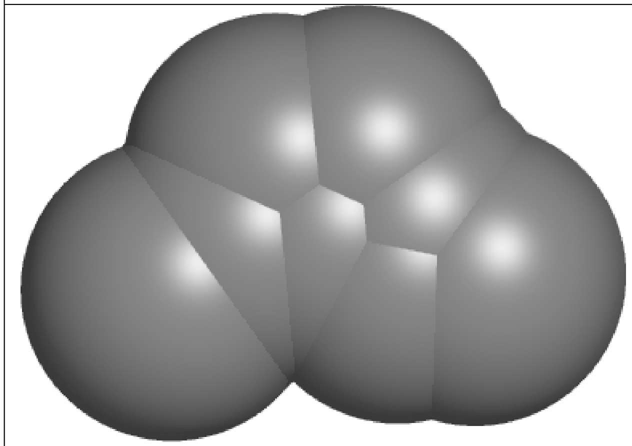 Bond lengths	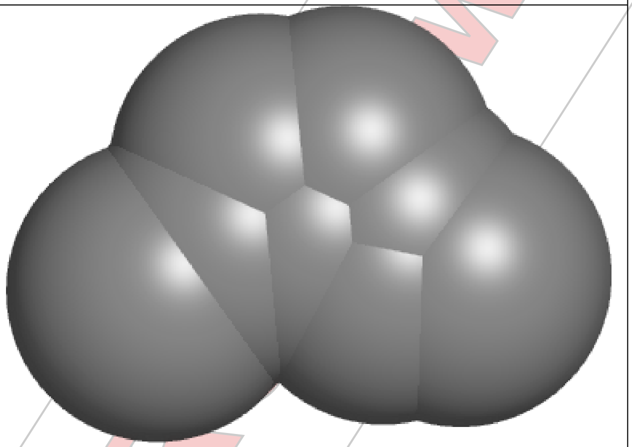 Bond angles
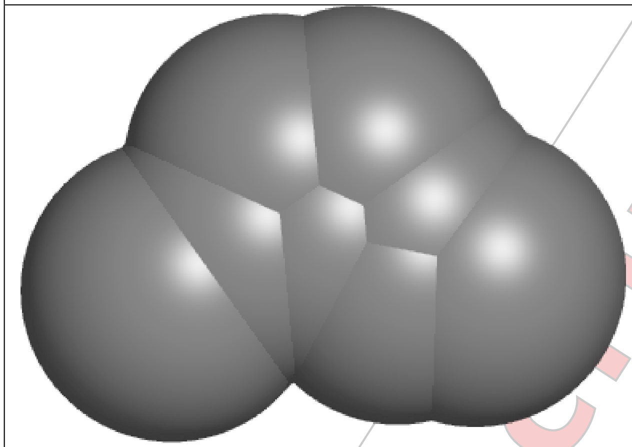 Torsions	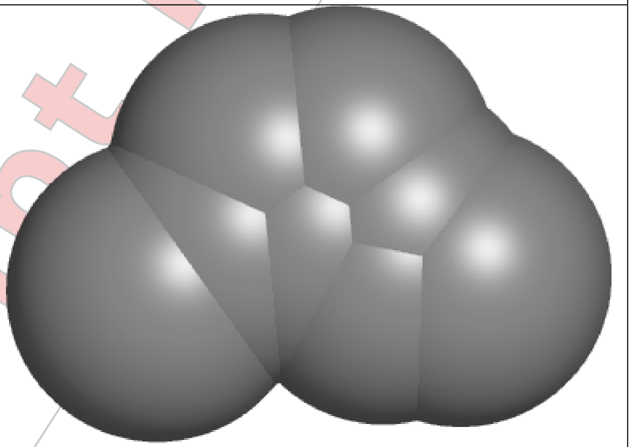 Rings

For Manuscript Review

Ligand FES A 606

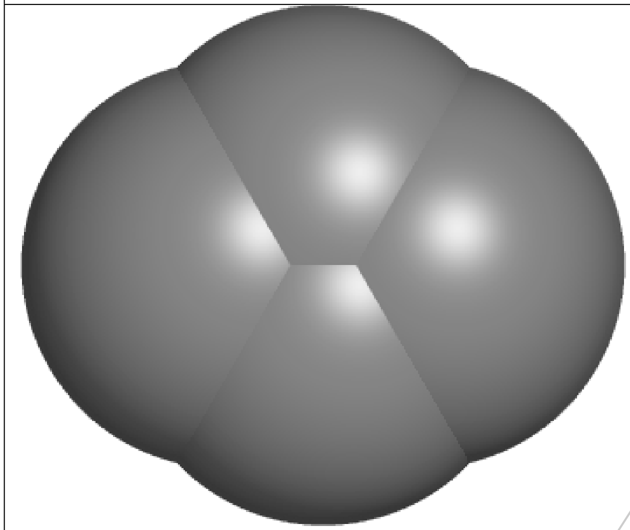

Bond lengths

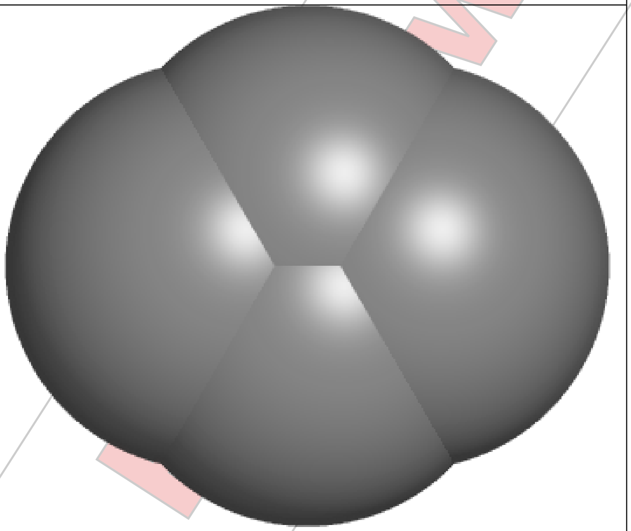

Bond angles

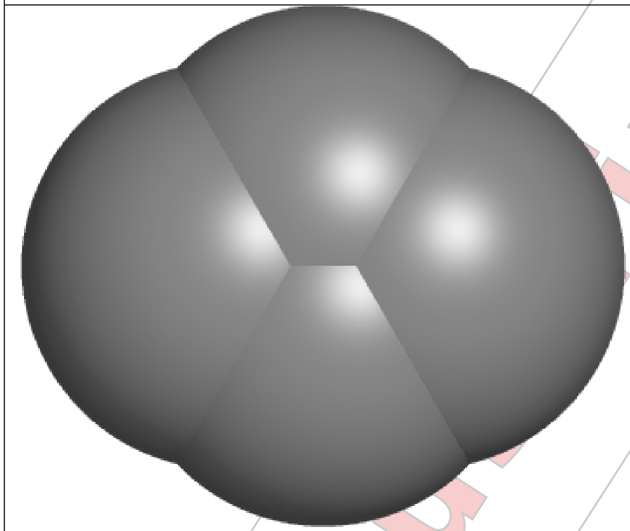

Torsions

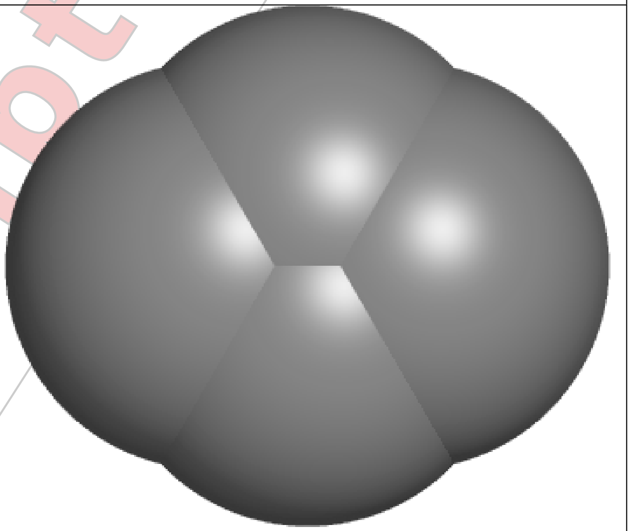

Rings

For Manuscript Review

Ligand SF4 B 603	
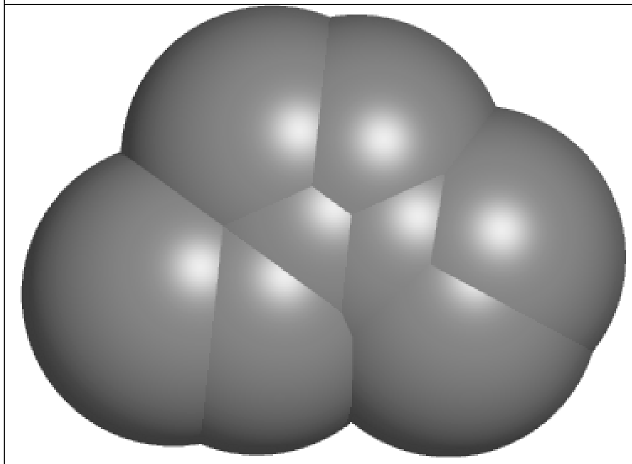 Bond lengths	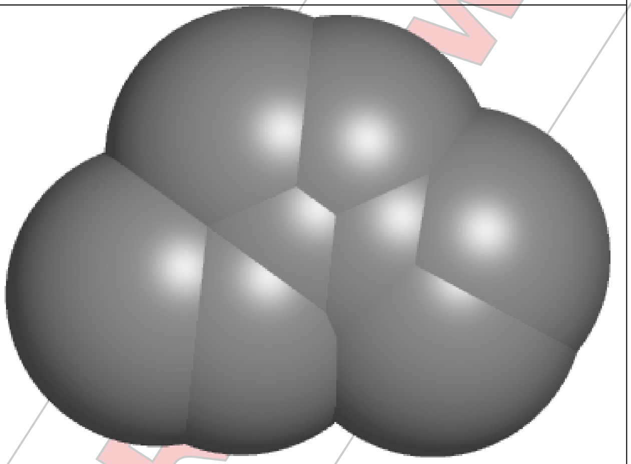 Bond angles
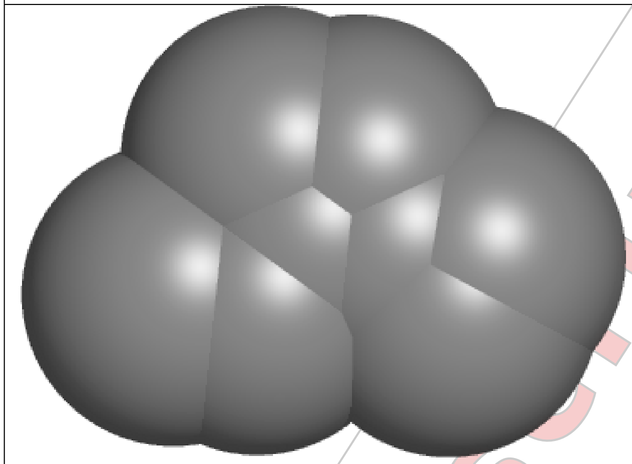 Torsions	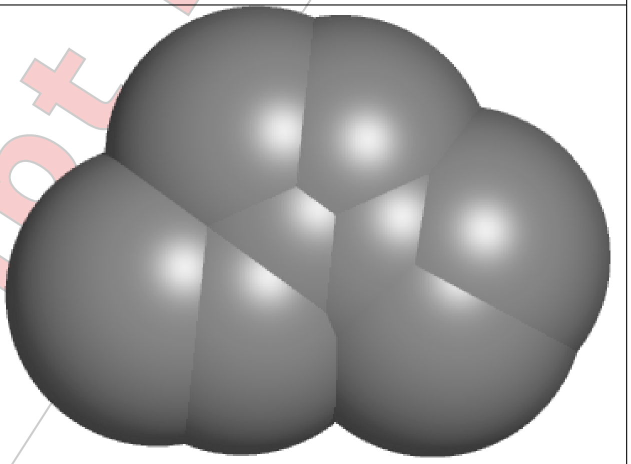 Rings

For Manuscript Review

Ligand FES B 606

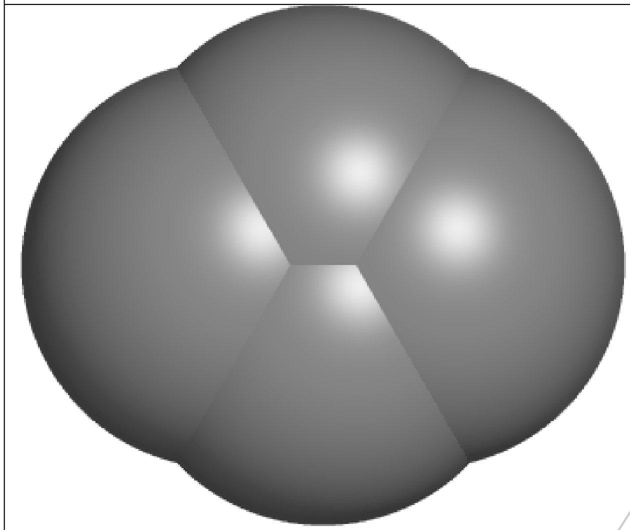

Bond lengths

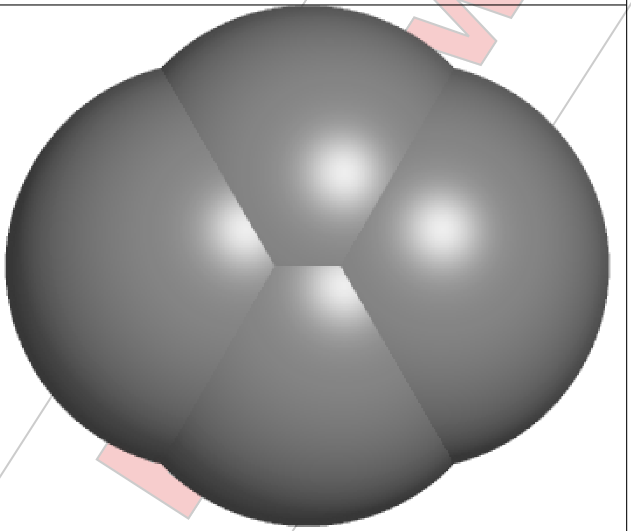

Bond angles

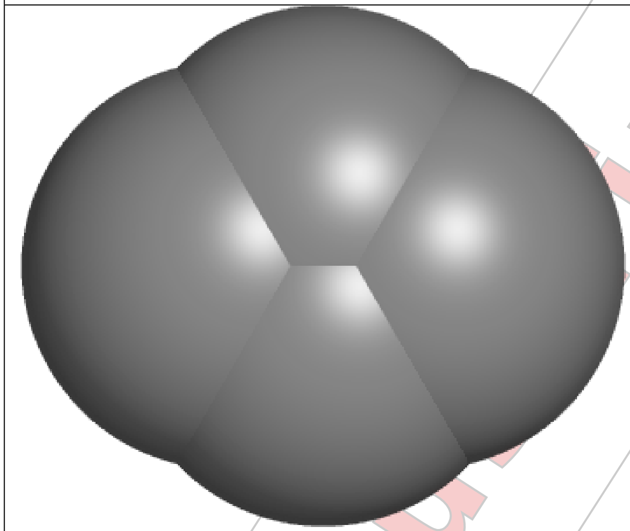

Torsions

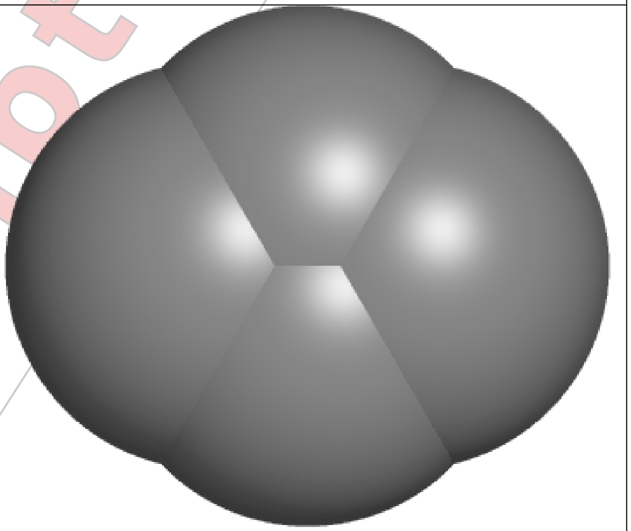

Rings

For Manuscript Review

Ligand SF4 B 605

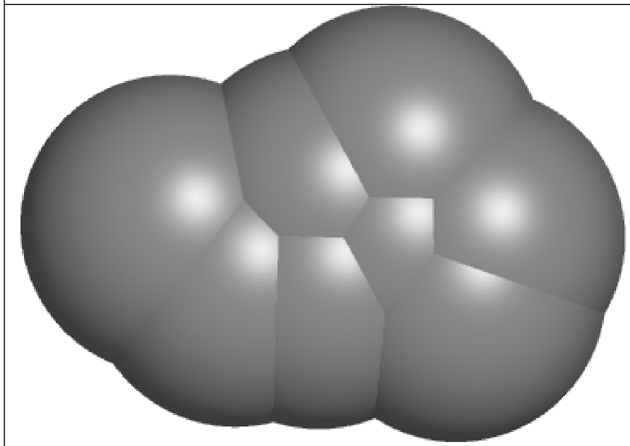

Bond lengths

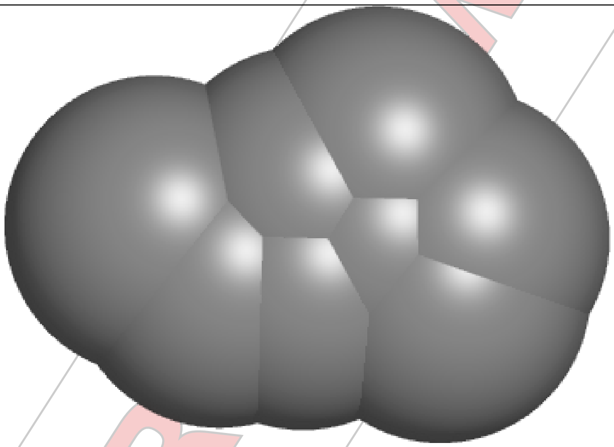

Bond angles

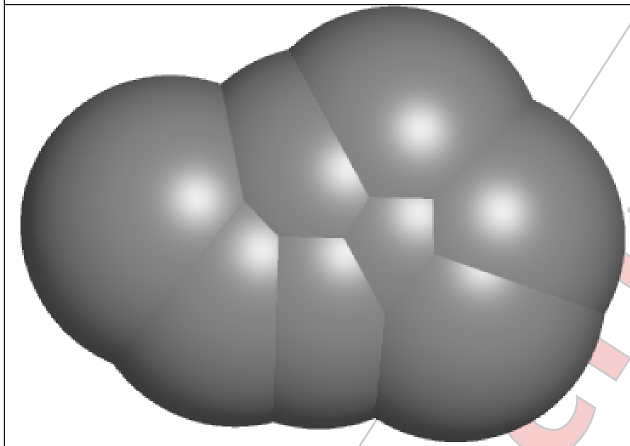

Torsions

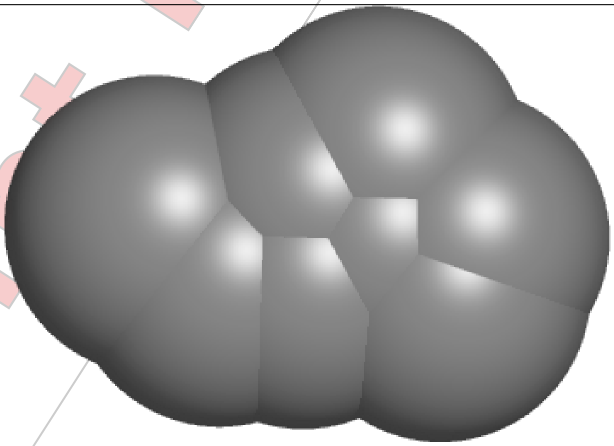

Rings

For Manuscript Review

Ligand SF4 A 605

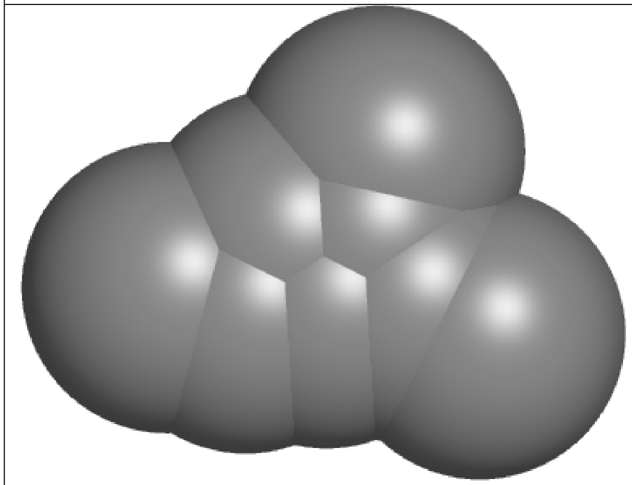

Bond lengths

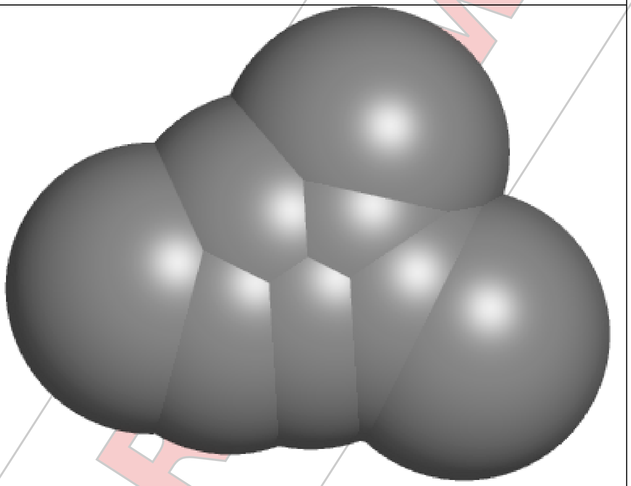

Bond angles

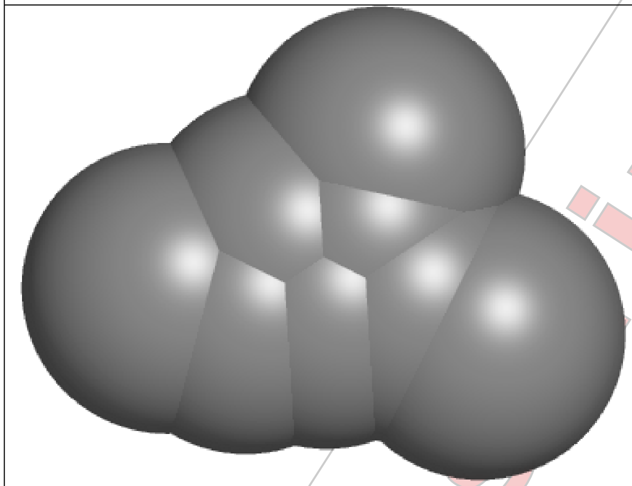

Torsions

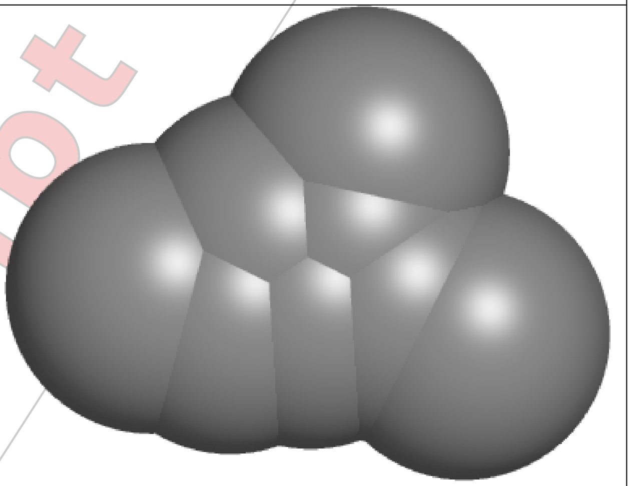

Rings

For Manuscript Review

Ligand MHX B 601	
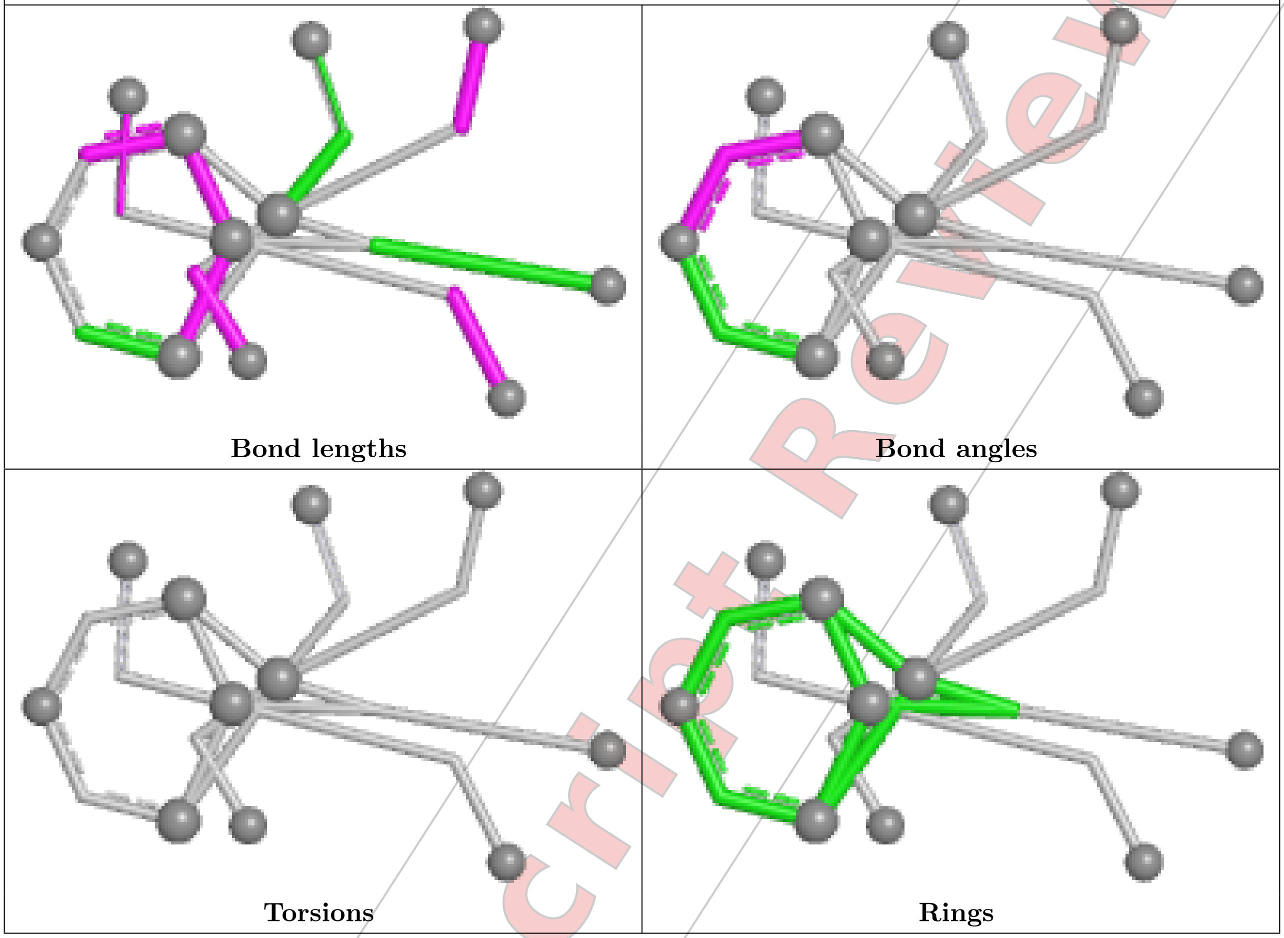	

Bond lengths

Bond angles

Torsions

Rings

For Manuscript Review

Ligand SF4 B 602

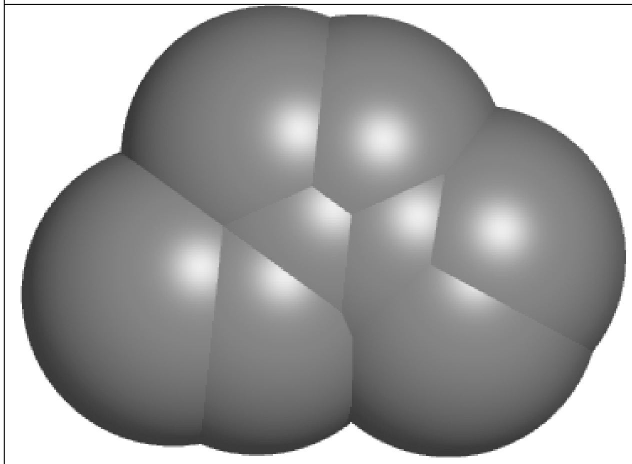

Bond lengths

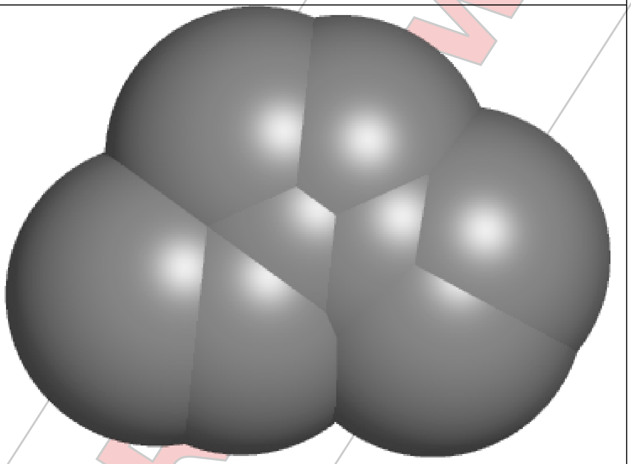

Bond angles

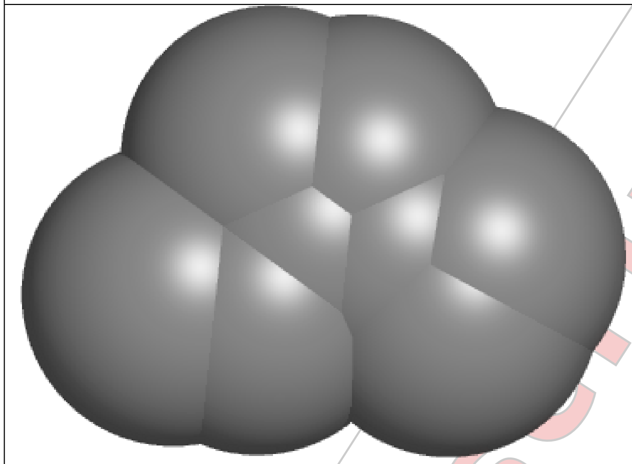

Torsions

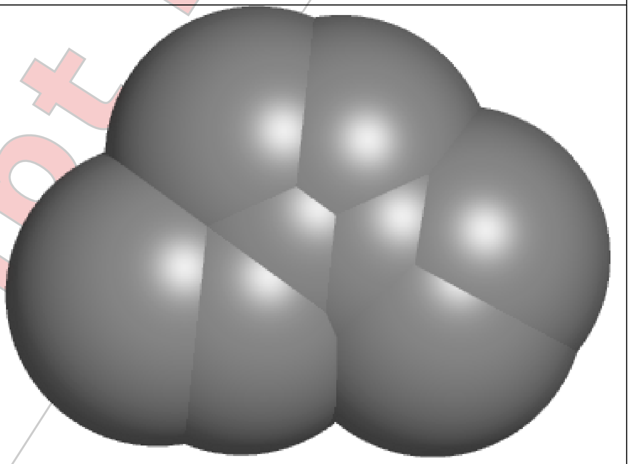

Rings

For Manuscript Review

Ligand SF4 A 602	
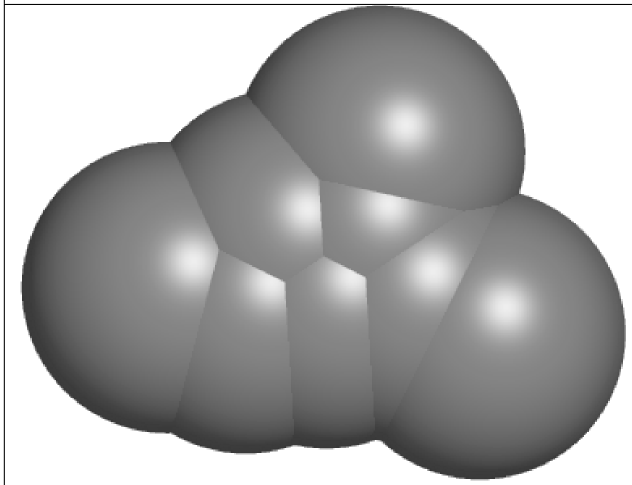 Bond lengths	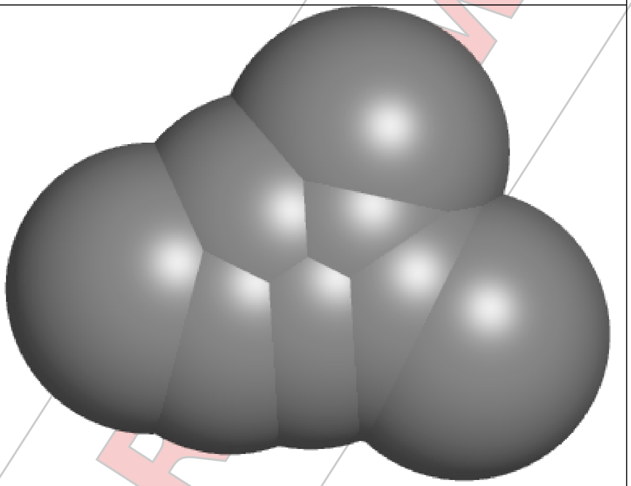 Bond angles
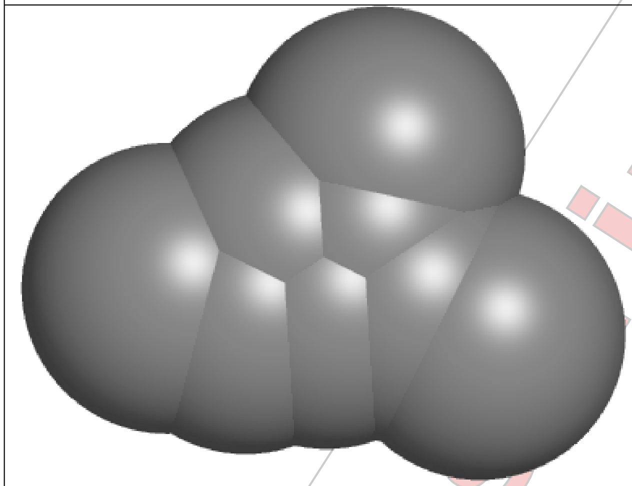 Torsions	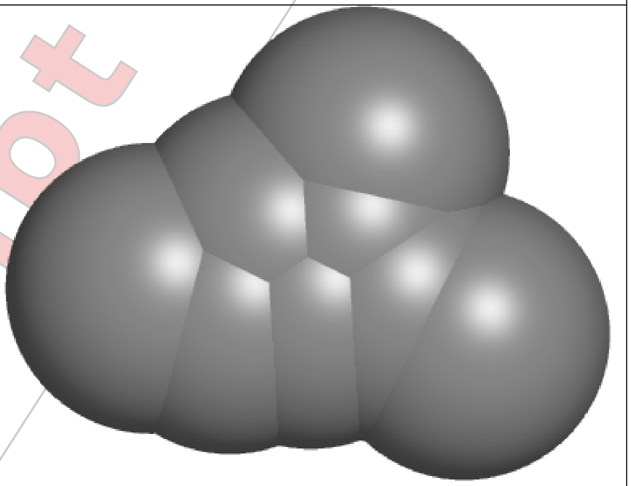 Rings

For Manuscript Review

Ligand SF4 A 604

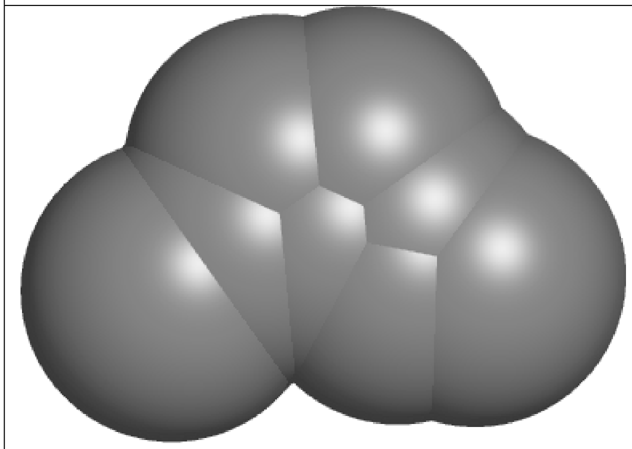

Bond lengths

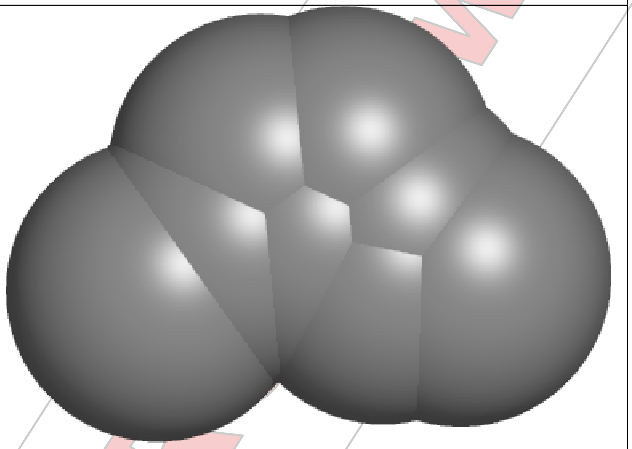

Bond angles

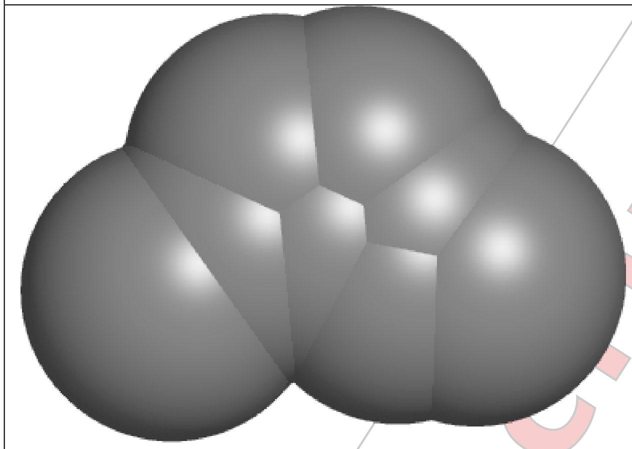

Torsions

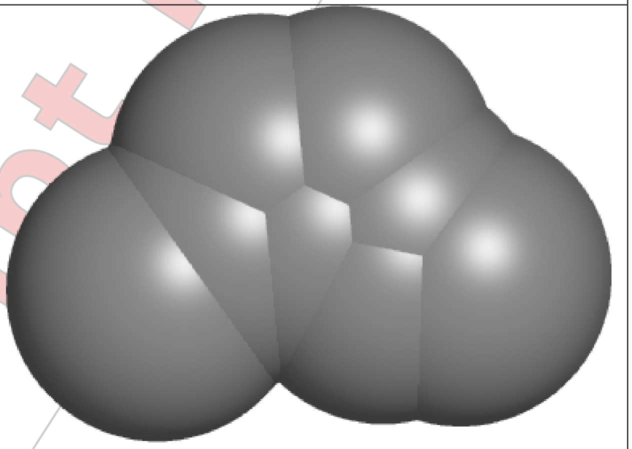

Rings

For Manuscript Review

Ligand MHX A 601

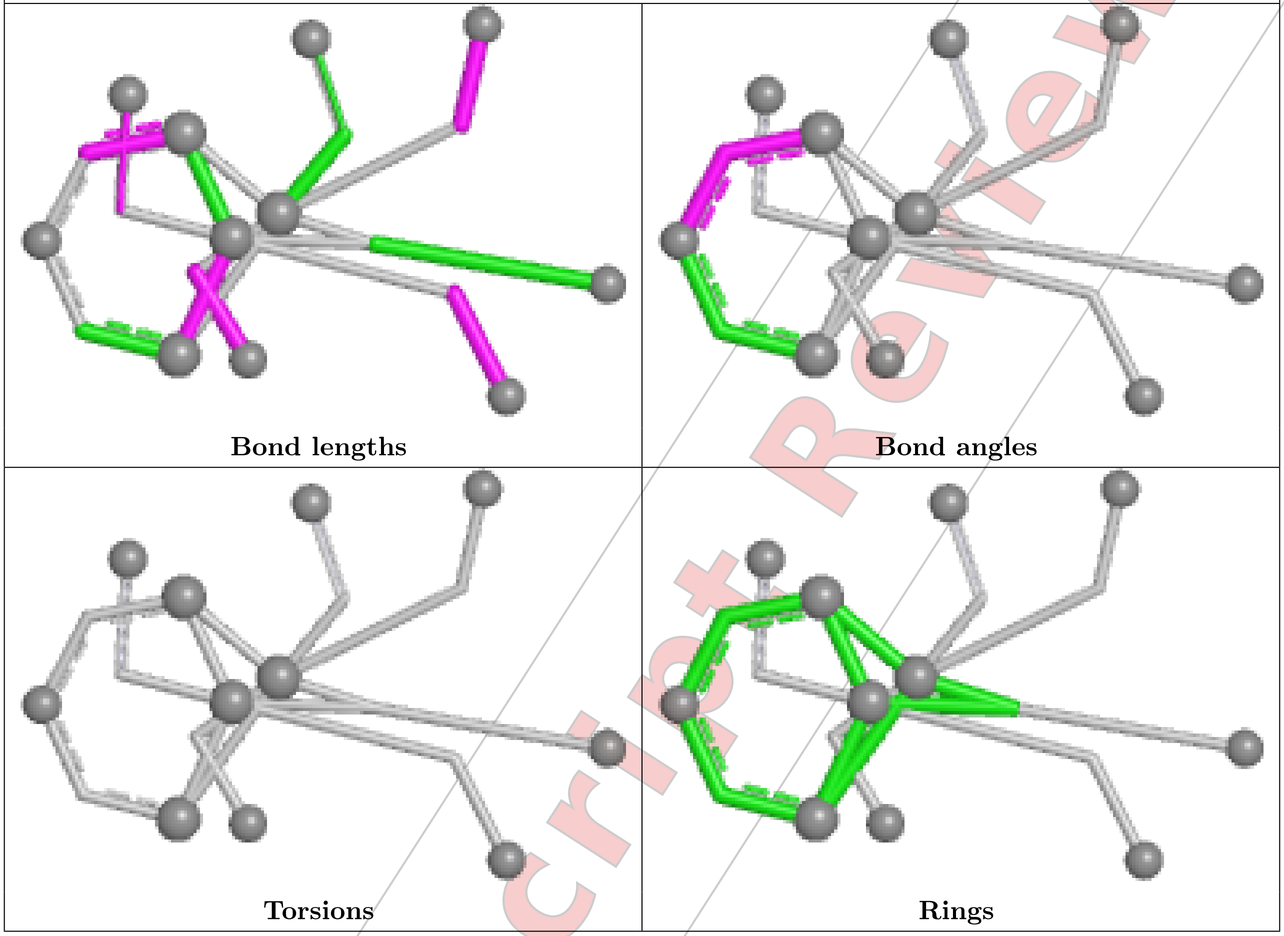

5.7 Other polymers (i)

There are no such residues in this entry.

5.8 Polymer linkage issues (i)

There are no chain breaks in this entry.

For Manuscript Review

6 Fit of model and data (i)

6.1 Protein, DNA and RNA chains (i)

In the following table, the column labelled '#RSRZ> 2' contains the number (and percentage) of RSRZ outliers, followed by percent RSRZ outliers for the chain as percentile scores relative to all X-ray entries and entries of similar resolution. The OWAB column contains the minimum, median, 95th percentile and maximum values of the occupancy-weighted average B-factor per residue. The column labelled 'Q< 0.9' lists the number of (and percentage) of residues with an average occupancy less than 0.9.

Mol	Chain	Analysed	<RSRZ>	#RSRZ>2			OWAB(Å^2)	Q<0.9
1	A	573/584 (98%)	0.04	20 (3%)	44	48	17, 26, 52, 67	0
1	B	573/584 (98%)	0.29	43 (7%)	14	15	16, 24, 60, 86	0
All	All	1146/1168 (98%)	0.17	63 (5%)	25	27	16, 26, 56, 86	0

All (63) RSRZ outliers are listed below:

Mol	Chain	Res	Type	RSRZ
1	B	5	ILE	10.2
1	B	9	VAL	9.0
1	B	8	GLY	8.9
1	B	4	ILE	8.8
1	B	6	ILE	7.8
1	B	3	THR	7.4
1	B	71	ILE	7.2
1	B	11	PHE	7.0
1	B	69	GLY	6.0
1	B	10	GLN	5.9
1	B	12	ASN	5.9
1	B	2	LYS	5.3
1	B	28	ILE	5.2
1	B	14	ASP	4.9
1	B	72	ILE	4.7
1	A	463	ASN	4.3
1	B	22	PHE	4.2
1	B	68	ASP	4.1
1	A	437	ASN	4.0
1	B	27	ASN	3.9
1	B	7	ASN	3.7
1	B	13	THR	3.6
1	B	53	VAL	3.6
1	B	29	ASP	3.5

Continued on next page...

For Manuscript Review

Continued from previous page...

Mol	Chain	Res	Type	RSRZ
1	A	515	LYS	3.5
1	A	435	ALA	3.3
1	B	172	ASN	3.2
1	B	79	VAL	3.2
1	A	290	ASN	3.2
1	A	464	ASN	3.2
1	B	127	LYS	3.1
1	B	75	ASN	3.0
1	A	513	ASN	2.9
1	B	573	LYS	2.8
1	B	574	LYS	2.8
1	B	56	THR	2.6
1	B	463	ASN	2.6
1	A	128	ASP	2.6
1	A	558	LYS	2.6
1	B	26	ASN	2.6
1	A	566	GLU	2.5
1	B	24	ARG	2.5
1	A	288	GLU	2.5
1	B	440	LEU	2.5
1	A	438	ALA	2.5
1	B	54	GLU	2.4
1	B	73	ASN	2.4
1	B	66	ILE	2.3
1	B	23	ALA	2.3
1	A	130	THR	2.3
1	A	122	LYS	2.2
1	B	58	LEU	2.2
1	A	561	GLU	2.2
1	B	140	LEU	2.1
1	A	441	GLU	2.1
1	B	174	LYS	2.1
1	A	563	ARG	2.1
1	B	67	GLU	2.1
1	B	57	GLY	2.1
1	A	289	ASN	2.1
1	A	4	ILE	2.1
1	A	242	ASN	2.0
1	B	70	MET	2.0

For Manuscript Review

6.2 Non-standard residues in protein, DNA, RNA chains (i)

There are no non-standard protein/DNA/RNA residues in this entry.

6.3 Carbohydrates (i)

There are no monosaccharides in this entry.

6.4 Ligands (i)

In the following table, the Atoms column lists the number of modelled atoms in the group and the number defined in the chemical component dictionary. The B-factors column lists the minimum, median, 95th percentile and maximum values of B factors of atoms in the group. The column labelled 'Q< 0.9' lists the number of atoms with occupancy less than 0.9.

Mol	Type	Chain	Res	Atoms	RSCC	RSR	B-factors(Å^2)	Q<0.9
5	GOL	B	608	6/6	0.92	0.15	23,34,42,50	0
5	GOL	A	607	6/6	0.94	0.08	23,31,41,44	0
3	SF4	A	605	8/8	0.97	0.07	23,25,27,28	0
6	MG	B	609	1/1	0.98	0.06	30,30,30,30	0
2	MHX	A	601	19/19	0.99	0.12	12,19,21,21	19
3	SF4	B	605	8/8	0.99	0.08	21,22,24,25	0
4	FES	B	606	4/4	0.99	0.10	23,25,25,25	0
2	MHX	B	601	19/19	0.99	0.13	10,16,18,21	19
3	SF4	A	603	8/8	0.99	0.10	17,18,19,19	0
6	MG	A	608	1/1	0.99	0.03	24,24,24,24	0
6	MG	A	609	1/1	0.99	0.06	21,21,21,21	0
6	MG	B	607	1/1	0.99	0.04	26,26,26,26	0
3	SF4	A	604	8/8	0.99	0.08	18,19,20,20	0
7	CL	A	610	1/1	0.99	0.08	31,31,31,31	0
7	CL	B	610	1/1	0.99	0.05	27,27,27,27	0
4	FES	A	606	4/4	1.00	0.10	20,21,22,22	0
3	SF4	B	602	8/8	1.00	0.12	16,17,18,18	0
3	SF4	B	603	8/8	1.00	0.11	16,17,18,19	0
3	SF4	B	604	8/8	1.00	0.10	18,19,19,19	0
3	SF4	A	602	8/8	1.00	0.11	17,18,19,19	0

The following is a graphical depiction of the model fit to experimental electron density of all instances of the Ligand of Interest. In addition, ligands with molecular weight > 250 and outliers as shown on the geometry validation Tables will also be included. Each fit is shown from different orientation to approximate a three-dimensional view.

For Manuscript Review

Electron density around SF4 A 605:

$2mF_o$-DF_c (at 0.7 rmsd) in gray
mF_o-DF_c (at 3 rmsd) in purple (negative)
and green (positive)

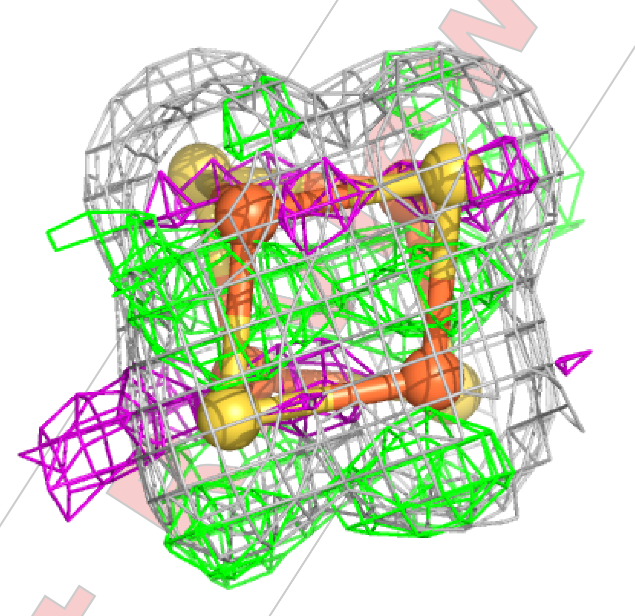

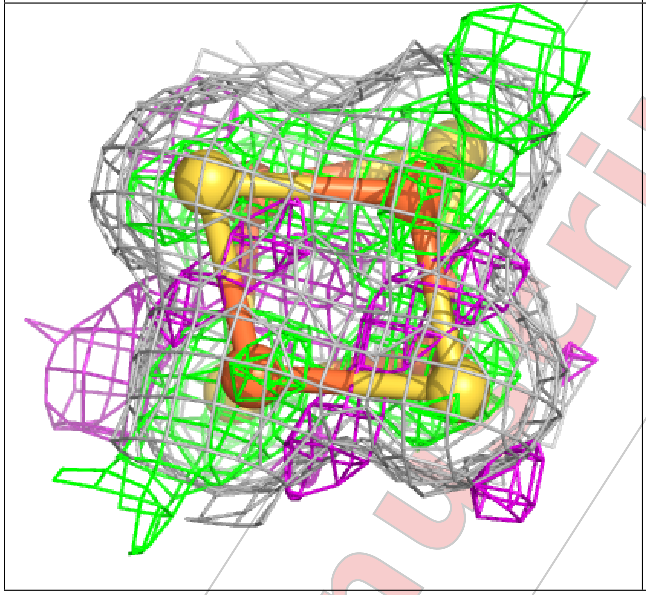

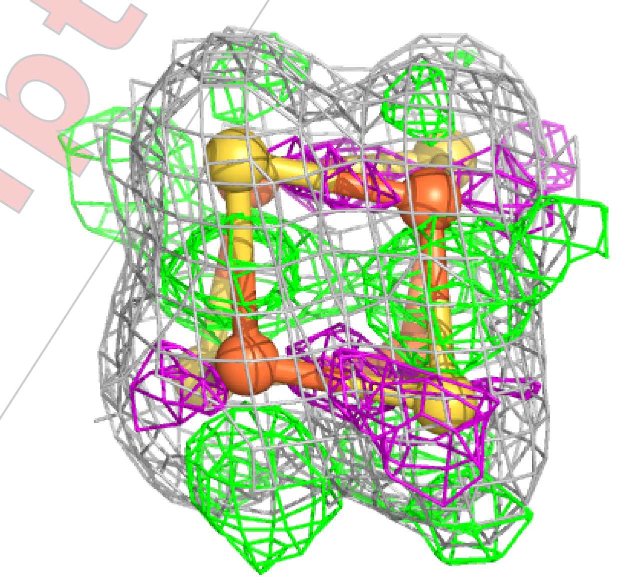

For Manuscript Review

**Electron density around MHX A 601:**<br><br>$2mF_o$-DF_c (at 0.7 rmsd) in gray<br>mF_o-DF_c (at 3 rmsd) in purple (negative) and green (positive)	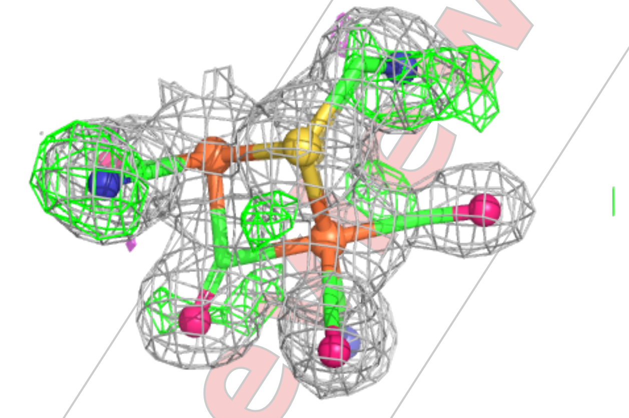
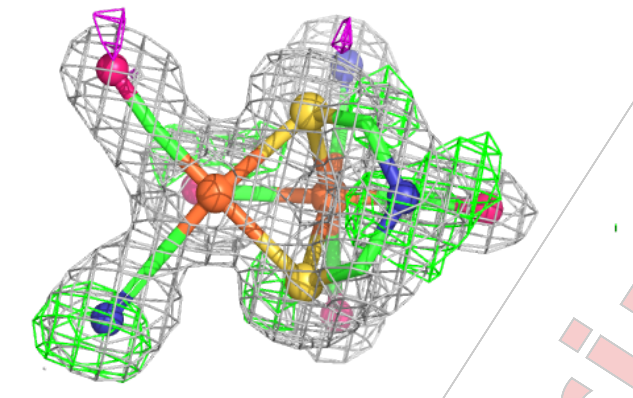	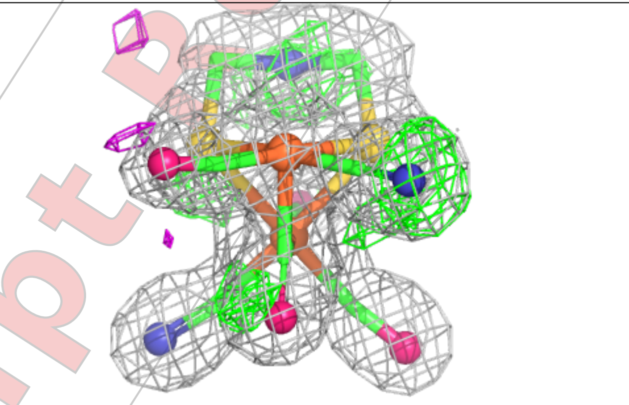

For Manuscript Review

Electron density around SF4 B 605:

$2mF_o$-DF_c (at 0.7 rmsd) in gray
mF_o-DF_c (at 3 rmsd) in purple (negative)
and green (positive)

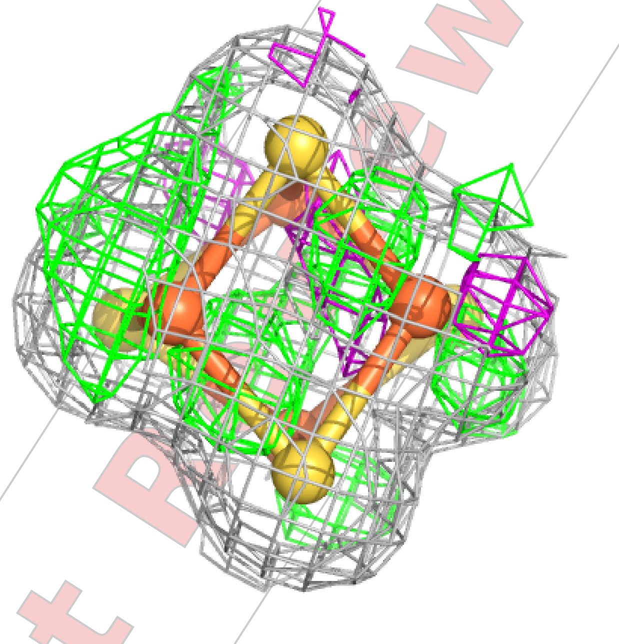

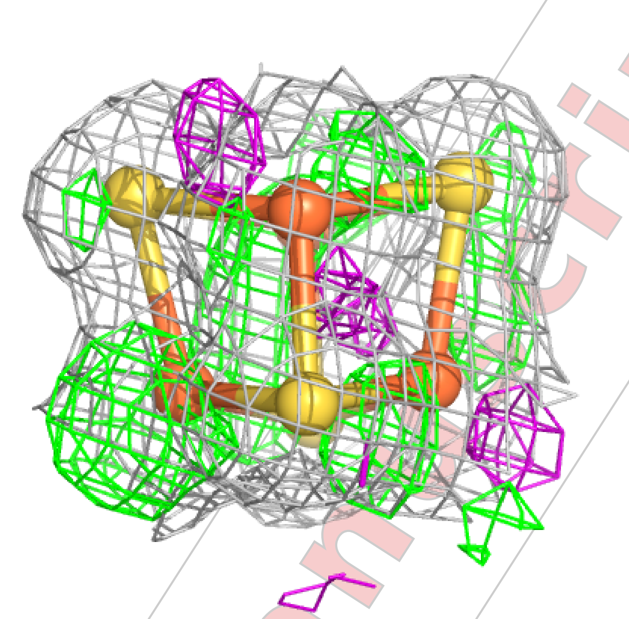

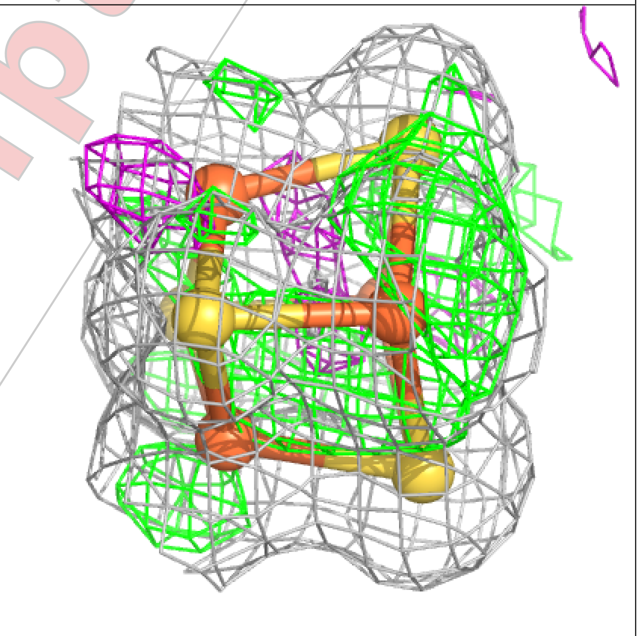

For Manuscript Review

Electron density around FES B 606:

$2mF_o$-DF_c (at 0.7 rmsd) in gray
mF_o-DF_c (at 3 rmsd) in purple (negative)
and green (positive)

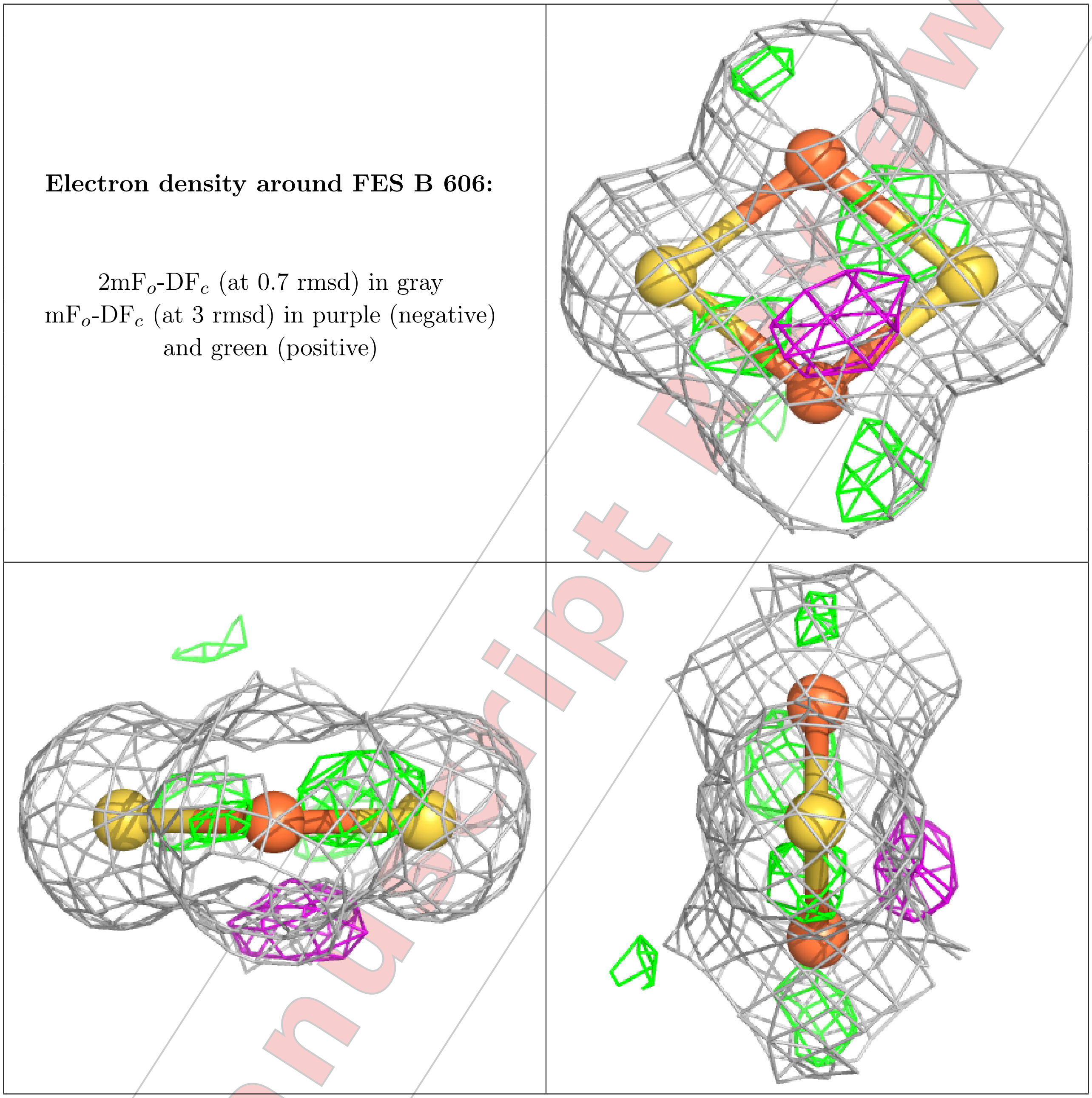

For Manuscript Review

Electron density around MHX B 601:

2mF$_o$-DF$_c$ (at 0.7 rmsd) in gray
mF$_o$-DF$_c$ (at 3 rmsd) in purple (negative)
and green (positive)

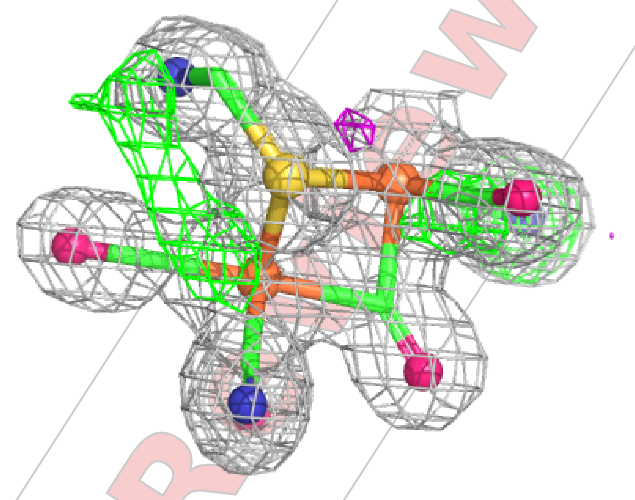

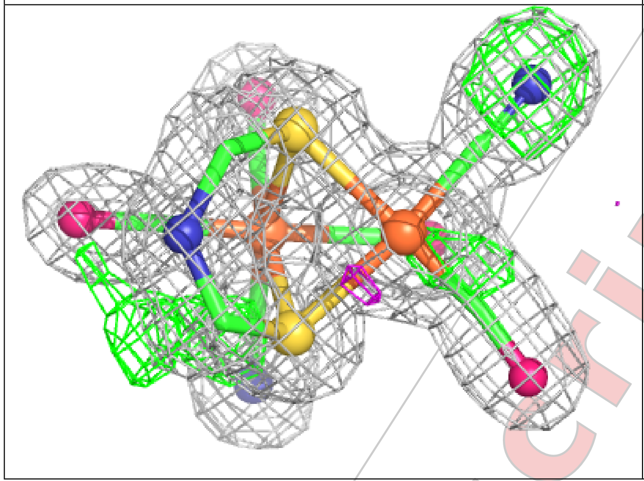

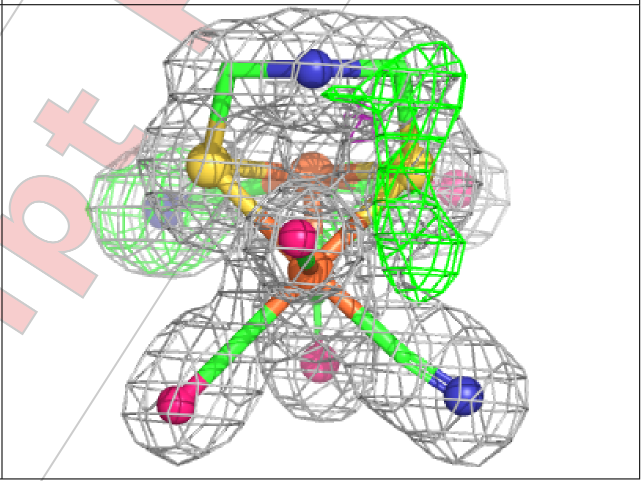

For Manuscript Review

Electron density around SF4 A 603:

$2mF_o$-DF_c (at 0.7 rmsd) in gray
mF_o-DF_c (at 3 rmsd) in purple (negative)
and green (positive)

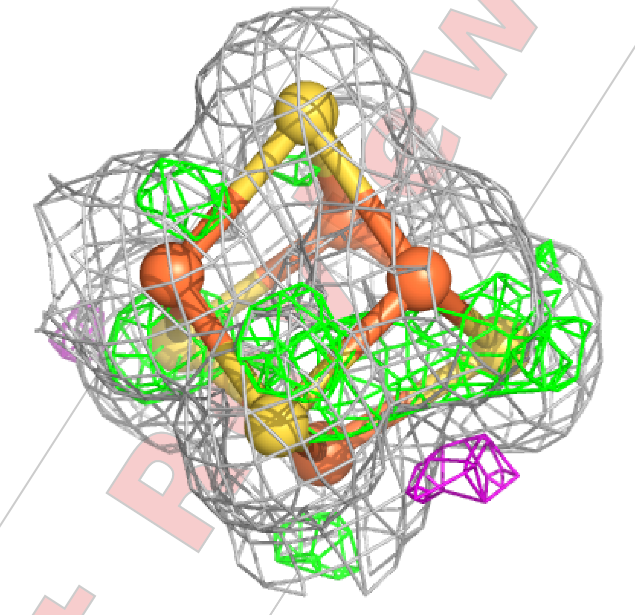

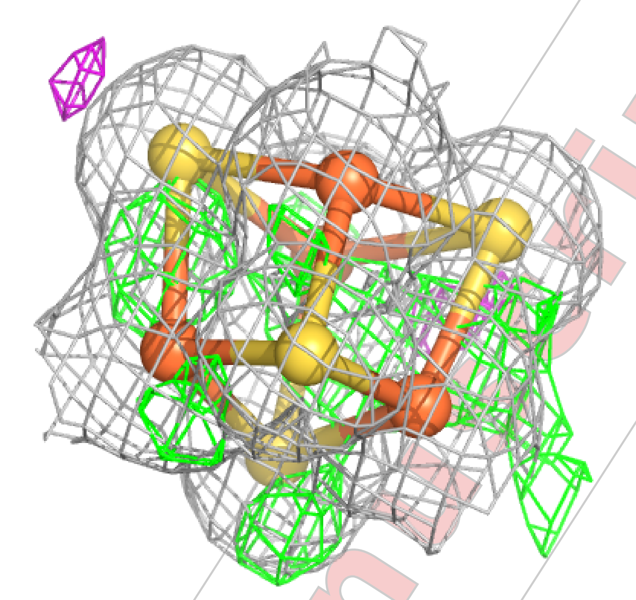

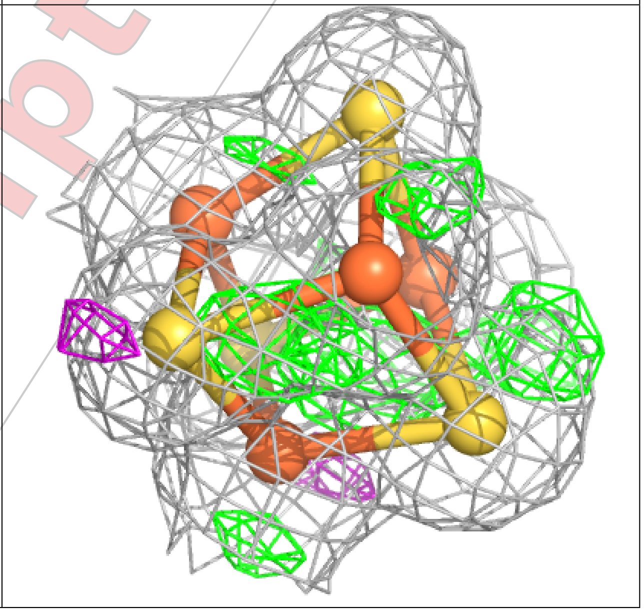

For Manuscript Review

Electron density around SF4 A 604:

$2mF_o$-DF_c (at 0.7 rmsd) in gray
mF_o-DF_c (at 3 rmsd) in purple (negative)
and green (positive)

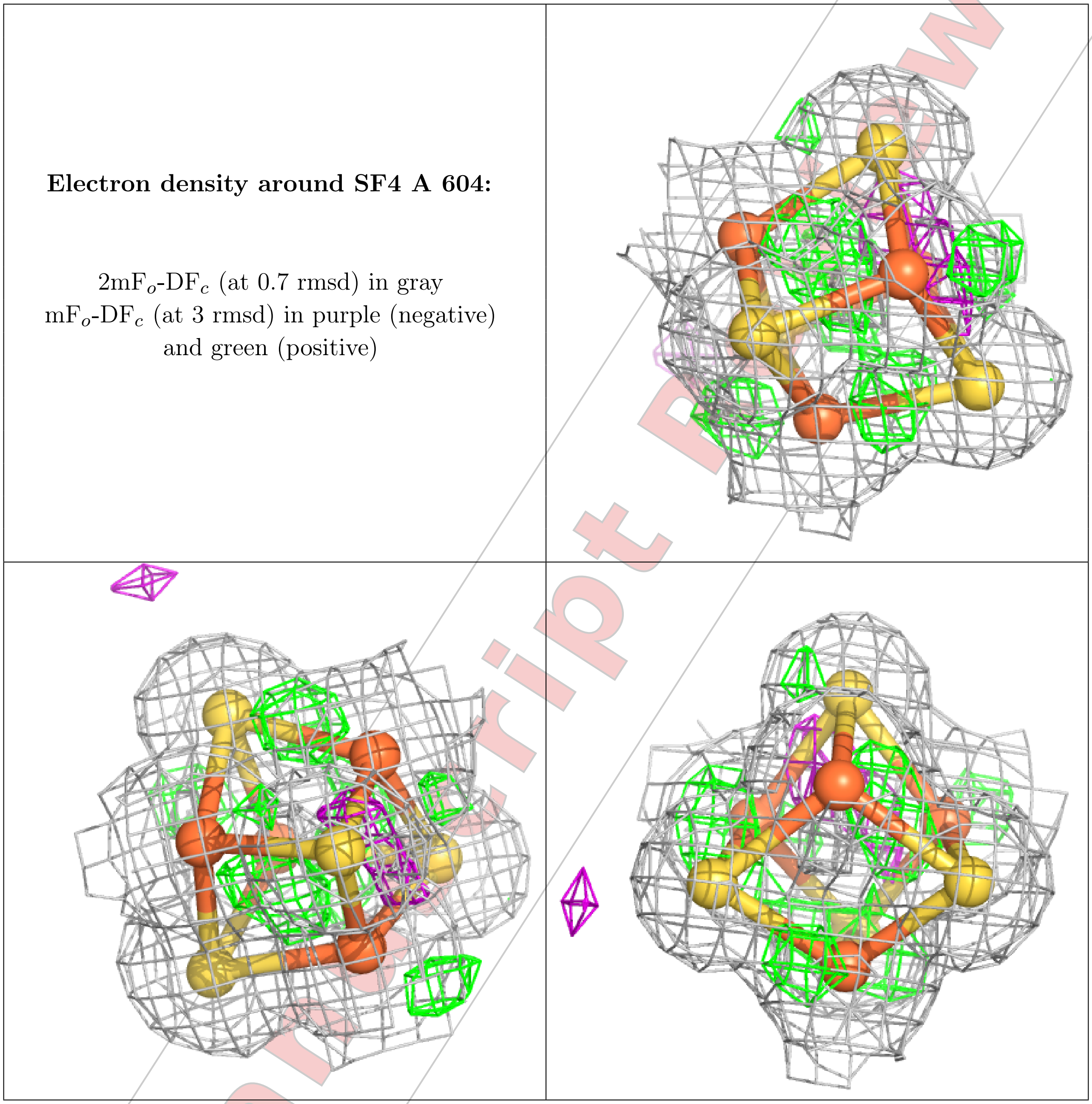

For Manuscript Review

Electron density around FES A 606:

$2mF_o$-DF_c (at 0.7 rmsd) in gray
mF_o-DF_c (at 3 rmsd) in purple (negative)
and green (positive)

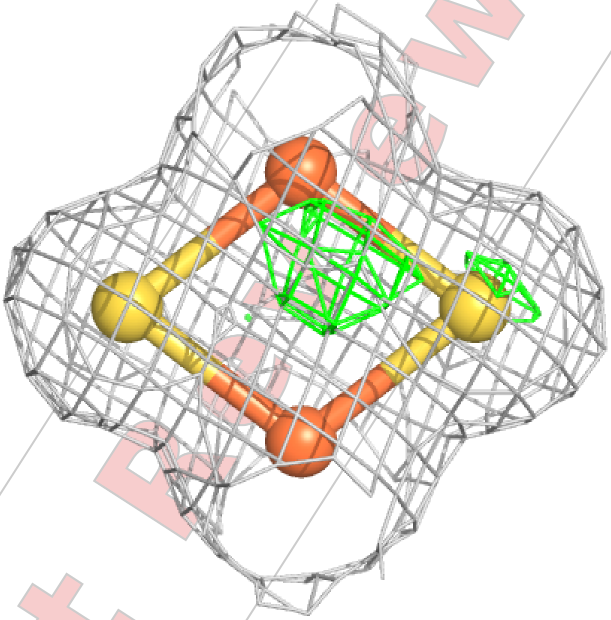

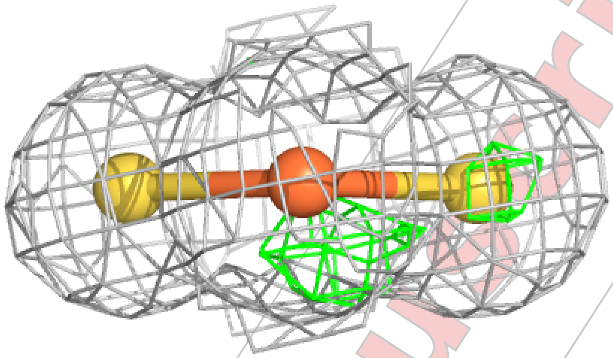

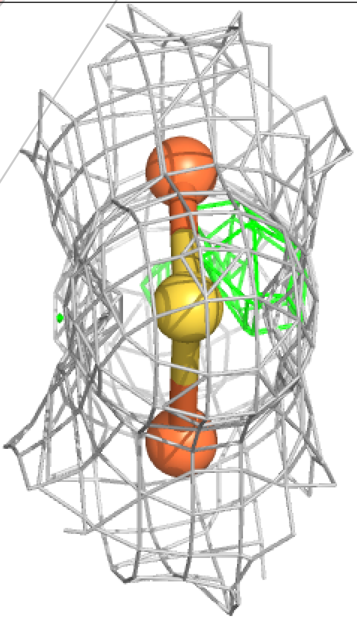

For Manuscript Review

Electron density around SF4 B 602:

$2mF_o$-DF_c (at 0.7 rmsd) in gray
mF_o-DF_c (at 3 rmsd) in purple (negative) and green (positive)

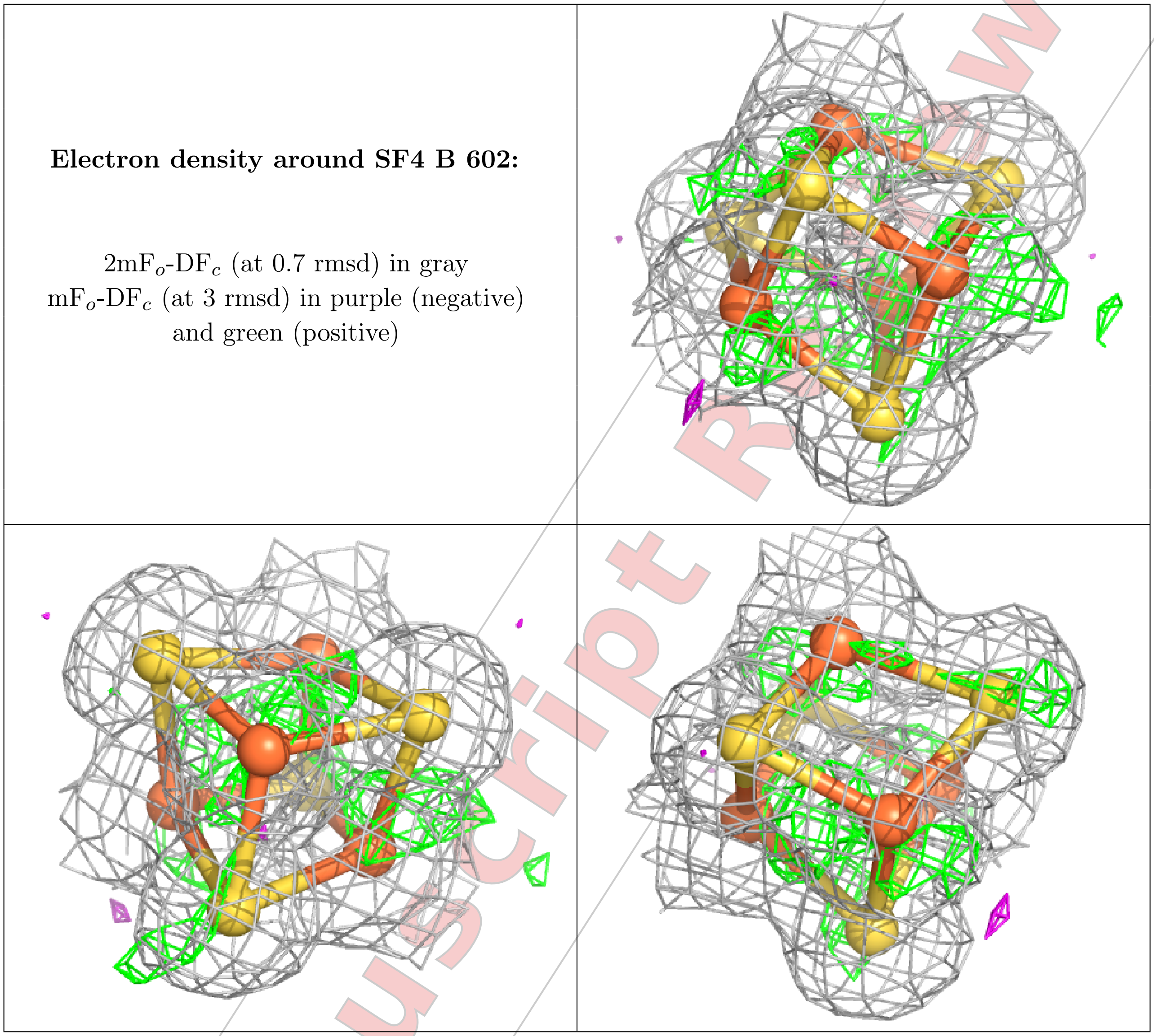

For Manuscript Review

Electron density around SF4 B 603:

2mF$_o$-DF$_c$ (at 0.7 rmsd) in gray
mF$_o$-DF$_c$ (at 3 rmsd) in purple (negative)
and green (positive)

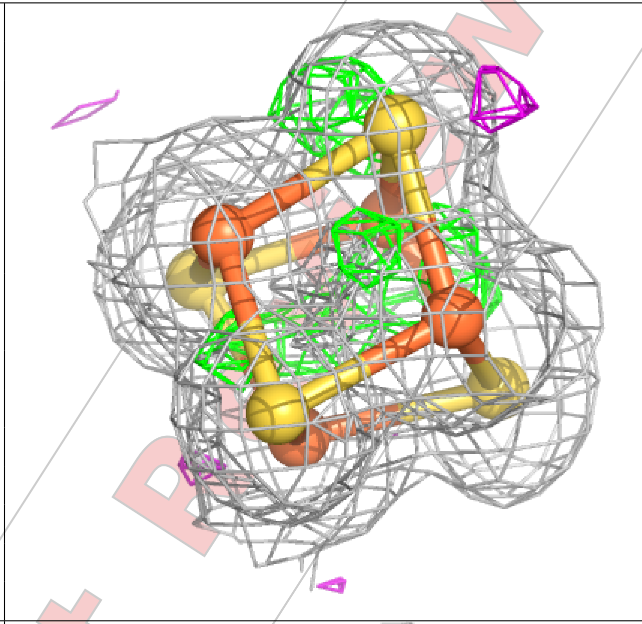

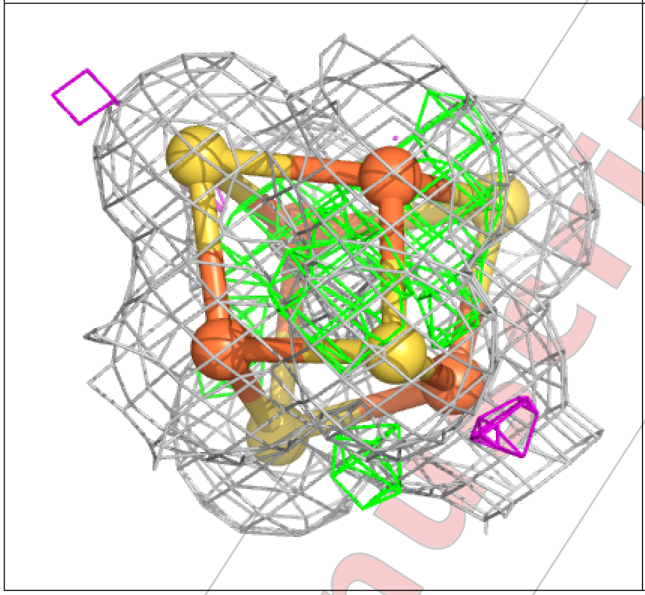

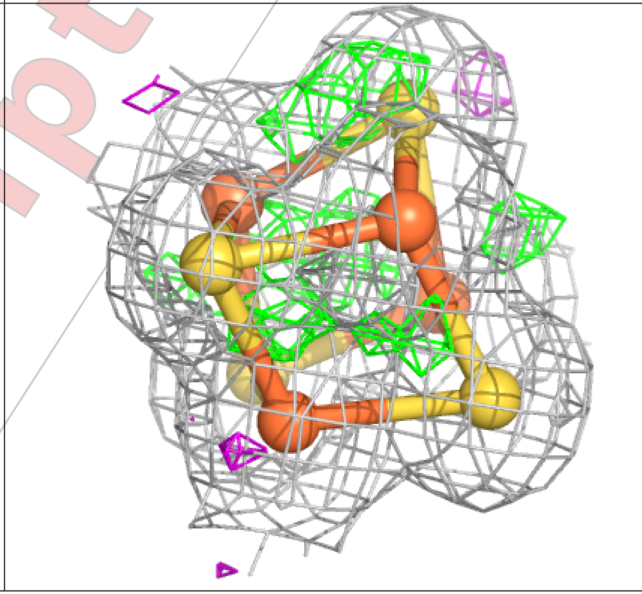

For Manuscript Review

Electron density around SF4 B 604:

$2mF_o$-DF_c (at 0.7 rmsd) in gray
mF_o-DF_c (at 3 rmsd) in purple (negative)
and green (positive)

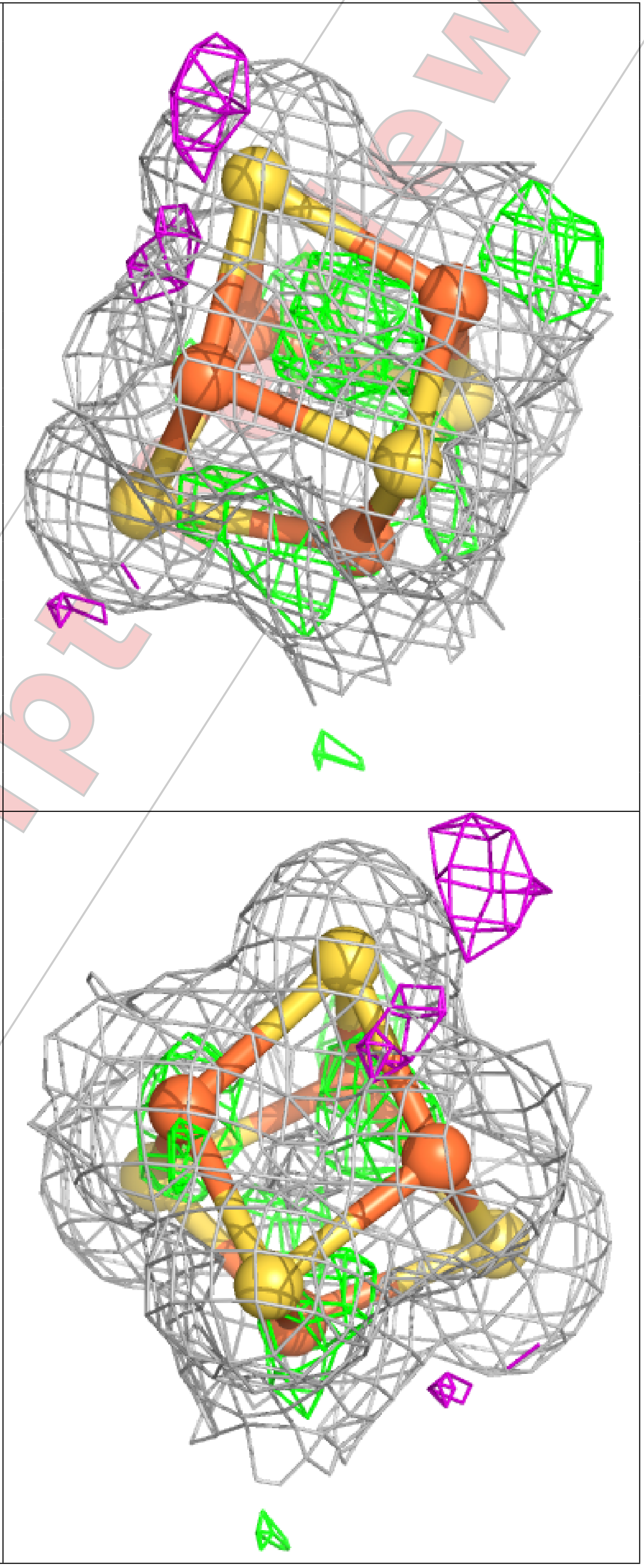

For Manuscript Review

Electron density around SF4 A 602:

$2mF_o$-DF_c (at 0.7 rmsd) in gray
mF_o-DF_c (at 3 rmsd) in purple (negative)
and green (positive)

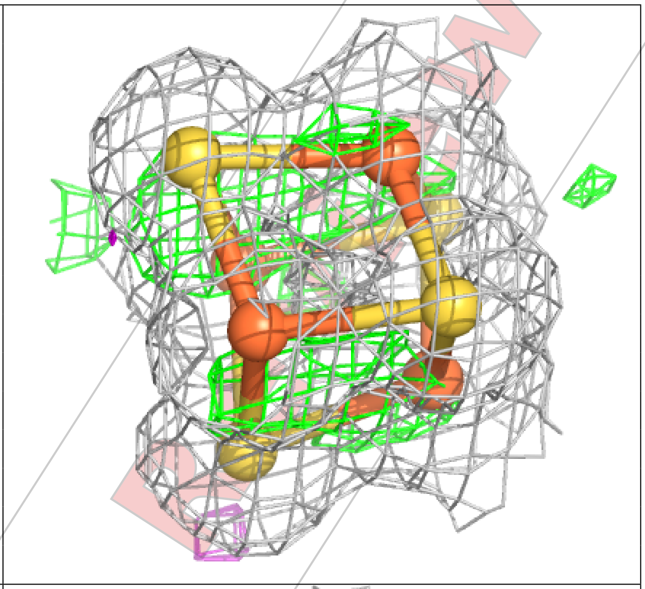

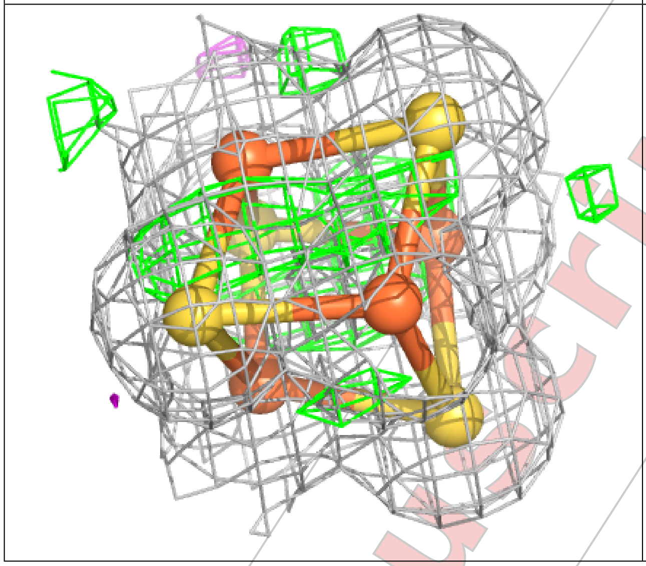

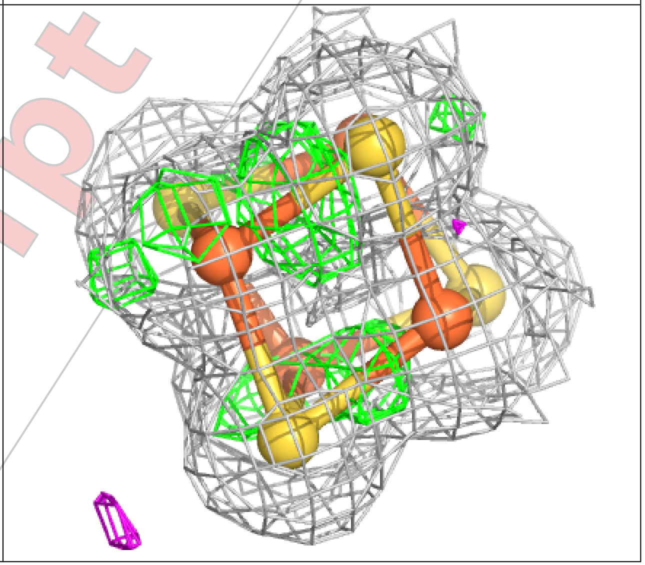

6.5 Other polymers (i)

There are no such residues in this entry.

For Manuscript Review
