# Supplementary material for: Cyanide Binding to [FeFe]‐Hydrogenase Stabilizes the Alternative Configuration of the Proton Transfer Pathway
Source: Angew Chem Int Ed Engl. 2023 Jan 10;62(7):e202216903. doi: 10.1002/anie.202216903 (PMC10107461; doi:10.1002/anie.202216903)
Supplement: Supplementary file 5 — Supporting Information [file ANIE-62-0-s002.pdf]

Full wwPDB X-ray Structure Validation Report (i)

Aug 30, 2022 – 05:23 pm BST

PDB ID	:	8AP2
Title	:	cyanide-bound [FeFe]-hydrogenase I from Clostridium pasteurianum (CpI)
Deposited on	:	2022-08-09
Resolution	:	1.39 Å(reported)

This wwPDB validation report is for manuscript review

This is a Full wwPDB X-ray Structure Validation Report.

This report is produced by the wwPDB biocuration pipeline after annotation of the structure.

We welcome your comments at *validation@mail.wwpdb.org*
A user guide is available at
https://www.wwpdb.org/validation/2017/XrayValidationReportHelp
with specific help available everywhere you see the (i) symbol.

The types of validation reports are described at http://www.wwpdb.org/validation/2017/FAQs#types.

The following versions of software and data (see references (i)) were used in the production of this report:

MolProbity	:	4.02b-467
Mogul	:	1.8.4, CSD as541be (2020)
Xtriage (Phenix)	:	1.13
EDS	:	2.30
buster-report	:	1.1.7 (2018)
Percentile statistics	:	20191225.v01 (using entries in the PDB archive December 25th 2019)
Refmac	:	5.8.0267
CCP4	:	7.1.010 (Gargrove)
Ideal geometry (proteins)	:	Engh & Huber (2001)
Ideal geometry (DNA, RNA)	:	Parkinson et al. (1996)
Validation Pipeline (wwPDB-VP)	:	2.30

For Manuscript Review

1 Overall quality at a glance (i)

The following experimental techniques were used to determine the structure:
X-RAY DIFFRACTION

The reported resolution of this entry is 1.39 Å.

Percentile scores (ranging between 0-100) for global validation metrics of the entry are shown in the following graphic. The table shows the number of entries on which the scores are based.

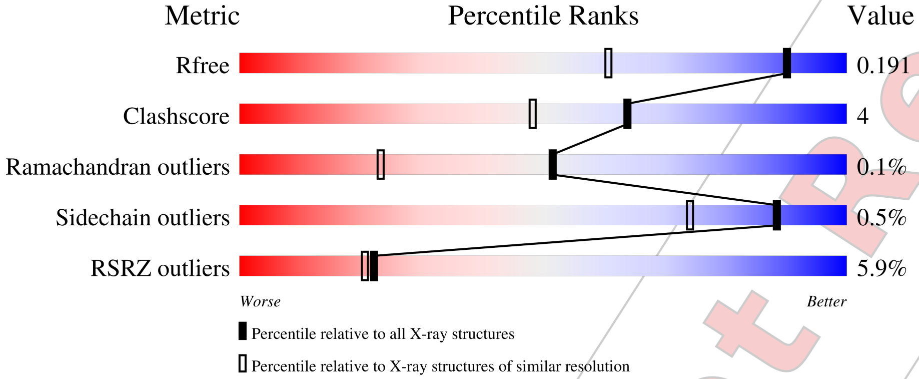

Metric	Whole archive (#Entries)	Similar resolution (#Entries, resolution range(Å))
R_{free}	130704	1714 (1.40-1.40)
Clashscore	141614	1812 (1.40-1.40)
Ramachandran outliers	138981	1763 (1.40-1.40)
Sidechain outliers	138945	1762 (1.40-1.40)
RSRZ outliers	127900	1674 (1.40-1.40)

The table below summarises the geometric issues observed across the polymeric chains and their fit to the electron density. The red, orange, yellow and green segments of the lower bar indicate the fraction of residues that contain outliers for >=3, 2, 1 and 0 types of geometric quality criteria respectively. A grey segment represents the fraction of residues that are not modelled. The numeric value for each fraction is indicated below the corresponding segment, with a dot representing fractions <=5% The upper red bar (where present) indicates the fraction of residues that have poor fit to the electron density. The numeric value is given above the bar.

Mol	Chain	Length	Quality of chain
1	A	584	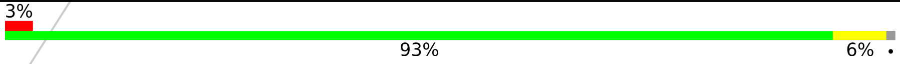
1	B	584	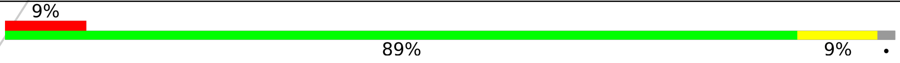

For Manuscript Review

2 Entry composition (i)

There are 9 unique types of molecules in this entry. The entry contains 10492 atoms, of which 0 are hydrogens and 0 are deuteriums.

In the tables below, the ZeroOcc column contains the number of atoms modelled with zero occupancy, the AltConf column contains the number of residues with at least one atom in alternate conformation and the Trace column contains the number of residues modelled with at most 2 atoms.

- Molecule 1 is a protein called Iron hydrogenase 1.

Mol	Chain	Residues	Atoms					ZeroOcc	AltConf	Trace
1	A	581	Total 4565	C 2857	N 785	O 880	S 43	0	7	0
1	B	573	Total 4543	C 2840	N 782	O 877	S 44	0	12	0

There are 20 discrepancies between the modelled and reference sequences:

Chain	Residue	Modelled	Actual	Comment	Reference
A	575	SER	-	expression tag	UNP P29166
A	576	ALA	-	expression tag	UNP P29166
A	577	TRP	-	expression tag	UNP P29166
A	578	SER	-	expression tag	UNP P29166
A	579	HIS	-	expression tag	UNP P29166
A	580	PRO	-	expression tag	UNP P29166
A	581	GLN	-	expression tag	UNP P29166
A	582	PHE	-	expression tag	UNP P29166
A	583	GLU	-	expression tag	UNP P29166
A	584	LYS	-	expression tag	UNP P29166
B	575	SER	-	expression tag	UNP P29166
B	576	ALA	-	expression tag	UNP P29166
B	577	TRP	-	expression tag	UNP P29166
B	578	SER	-	expression tag	UNP P29166
B	579	HIS	-	expression tag	UNP P29166
B	580	PRO	-	expression tag	UNP P29166
B	581	GLN	-	expression tag	UNP P29166
B	582	PHE	-	expression tag	UNP P29166
B	583	GLU	-	expression tag	UNP P29166
B	584	LYS	-	expression tag	UNP P29166

- Molecule 2 is dicarbonyl[bis(cyanide-kappaC)]-mu-(iminodimethanethiolatato-1kappaS:2kappaS)-mu-(oxomethylidene)diiron(2+) (three-letter code: 402) (formula: $C_7H_5Fe_2N_3O_3S_2$) (labeled as "Ligand of Interest" by depositor).

For Manuscript Review

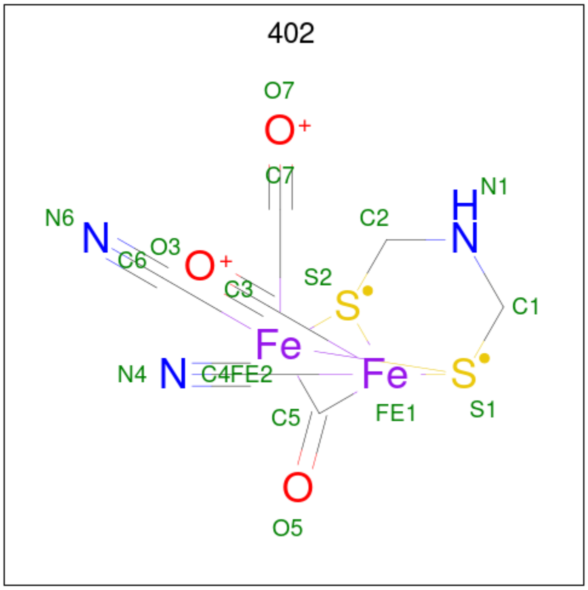

Mol	Chain	Residues	Atoms						ZeroOcc	AltConf
			Total	C	Fe	N	O	S		
2	A	1	17	7	2	3	3	2	0	0
2	B	1	17	7	2	3	3	2	0	0

- Molecule 3 is IRON/SULFUR CLUSTER (three-letter code: SF4) (formula: Fe_4S_4).

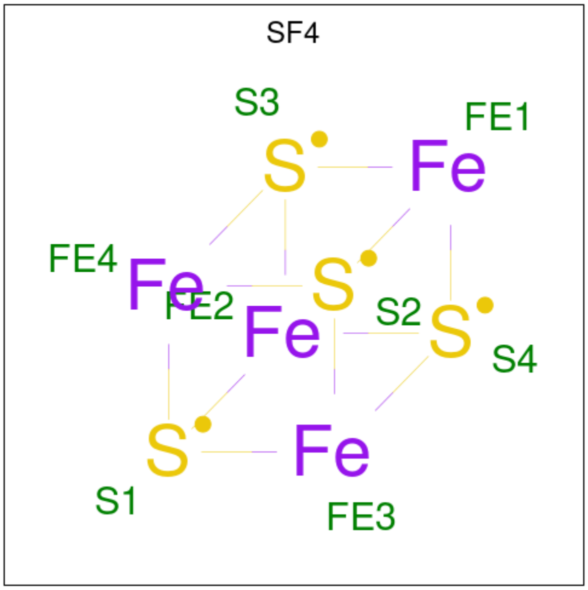

Mol	Chain	Residues	Atoms			ZeroOcc	AltConf
			Total	Fe	S		
3	A	1	8	4	4	0	0
3	A	1	8	4	4	0	0

Continued on next page...

For Manuscript Review

Continued from previous page...

Mol	Chain	Residues	Atoms	ZeroOcc	AltConf
3	A	1	Total Fe S<br>8 4 4	0	0
3	A	1	Total Fe S<br>8 4 4	0	0
3	B	1	Total Fe S<br>8 4 4	0	0
3	B	1	Total Fe S<br>8 4 4	0	0
3	B	1	Total Fe S<br>8 4 4	0	0
3	B	1	Total Fe S<br>8 4 4	0	0

- Molecule 4 is FE2/S2 (INORGANIC) CLUSTER (three-letter code: FES) (formula: Fe_2S_2).

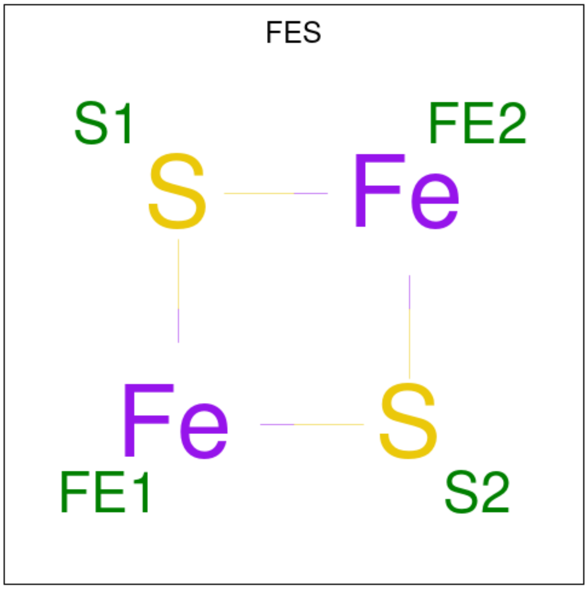

Mol	Chain	Residues	Atoms	ZeroOcc	AltConf
4	A	1	Total Fe S<br>4 2 2	0	0
4	B	1	Total Fe S<br>4 2 2	0	0

- Molecule 5 is CYANIDE ION (three-letter code: CYN) (formula: CN) (labeled as "Ligand of Interest" by depositor).

For Manuscript Review

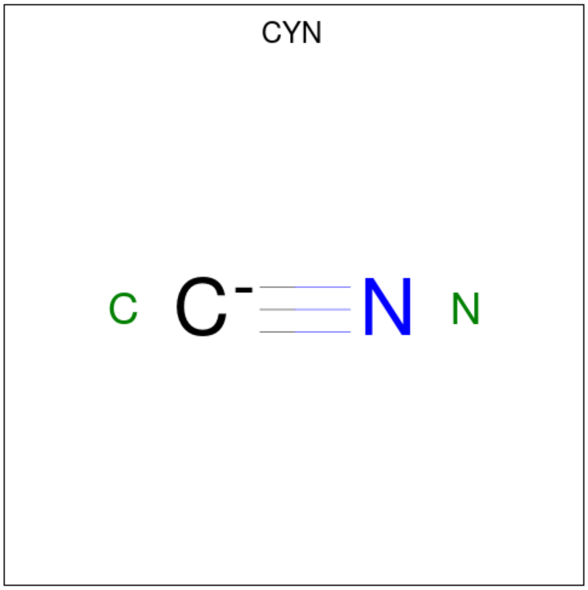

Mol	Chain	Residues	Atoms	ZeroOcc	AltConf
5	A	1	Total C N<br>2 1 1	0	0
5	B	1	Total C N<br>2 1 1	0	0

- Molecule 6 is GLYCEROL (three-letter code: GOL) (formula: $C_3H_8O_3$).

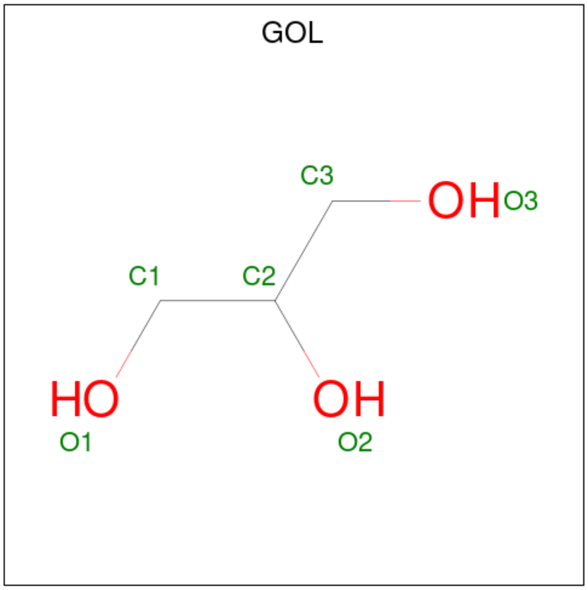

Mol	Chain	Residues	Atoms	ZeroOcc	AltConf
6	A	1	Total C O<br>6 3 3	0	0
6	B	1	Total C O<br>6 3 3	0	0

For Manuscript Review

- Molecule 7 is CHLORIDE ION (three-letter code: CL) (formula: Cl).

Mol	Chain	Residues	Atoms	ZeroOcc	AltConf
7	A	1	Total Cl<br>1 1	0	0
7	B	2	Total Cl<br>2 2	0	0

- Molecule 8 is MAGNESIUM ION (three-letter code: MG) (formula: Mg).

Mol	Chain	Residues	Atoms	ZeroOcc	AltConf
8	A	4	Total Mg<br>4 4	0	0
8	B	4	Total Mg<br>4 4	0	0

- Molecule 9 is water.

Mol	Chain	Residues	Atoms	ZeroOcc	AltConf
9	A	649	Total O<br>649 649	0	0
9	B	602	Total O<br>602 602	0	0

For Manuscript Review

3 Residue-property plots (i)

These plots are drawn for all protein, RNA, DNA and oligosaccharide chains in the entry. The first graphic for a chain summarises the proportions of the various outlier classes displayed in the second graphic. The second graphic shows the sequence view annotated by issues in geometry and electron density. Residues are color-coded according to the number of geometric quality criteria for which they contain at least one outlier: green = 0, yellow = 1, orange = 2 and red = 3 or more. A red dot above a residue indicates a poor fit to the electron density (RSRZ > 2). Stretches of 2 or more consecutive residues without any outlier are shown as a green connector. Residues present in the sample, but not in the model, are shown in grey.

- Molecule 1: Iron hydrogenase 1

Chain A:

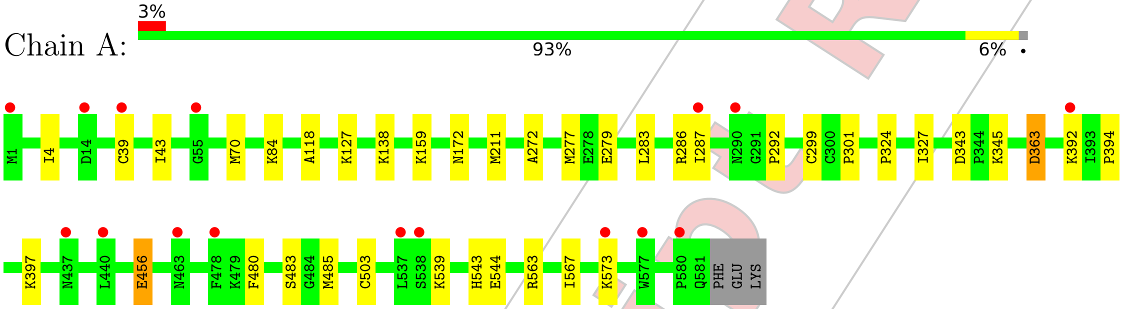

- Molecule 1: Iron hydrogenase 1

Chain B:

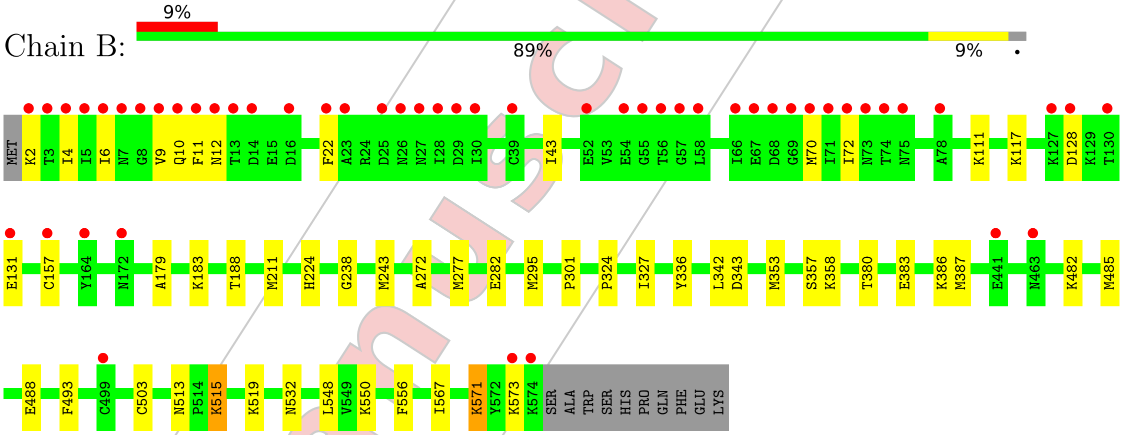

For Manuscript Review

4 Data and refinement statistics (i)

Property	Value	Source
Space group	P 1 21 1	Depositor
Cell constants<br>a, b, c, α, β, γ	88.07Å 71.50Å 102.78Å<br>90.00° 100.75° 90.00°	Depositor
Resolution (Å)	46.13 – 1.39<br>47.65 – 1.39	Depositor<br>EDS
% Data completeness<br>(in resolution range)	99.9 (46.13-1.39)<br>99.9 (47.65-1.39)	Depositor<br>EDS
R_{merge}	0.14	Depositor
R_{sym}	(Not available)	Depositor
$< I/\sigma(I) >$ [1]	1.31 (at 1.39Å)	Xtriage
Refinement program	PHENIX 1.19.1_4122	Depositor
R, R_{free}	0.159 , 0.191<br>0.159 , 0.191	Depositor<br>DCC
R_{free} test set	12582 reflections (5.00%)	wwPDB-VP
Wilson B-factor (Å^2)	15.9	Xtriage
Anisotropy	0.260	Xtriage
Bulk solvent k_{sol}(e/Å^3), B_{sol}(Å^2)	(Not available) , (Not available)	EDS
L-test for twinning[2]	$< \|L\| > = 0.50$, $< L^2 > = 0.33$	Xtriage
Estimated twinning fraction	No twinning to report.	Xtriage
F_o,F_c correlation	0.97	EDS
Total number of atoms	10492	wwPDB-VP
Average B, all atoms (Å^2)	26.0	wwPDB-VP

Xtriage's analysis on translational NCS is as follows: *The largest off-origin peak in the Patterson function is 6.03% of the height of the origin peak. No significant pseudotranslation is detected.*

[1] Intensities estimated from amplitudes.

[2] Theoretical values of $< |L| >$, $< L^2 >$ for acentric reflections are 0.5, 0.333 respectively for untwinned datasets, and 0.375, 0.2 for perfectly twinned datasets.

For Manuscript Review

5 Model quality (i)

5.1 Standard geometry (i)

Bond lengths and bond angles in the following residue types are not validated in this section: MG, GOL, 402, SF4, CYN, CL, FES

The Z score for a bond length (or angle) is the number of standard deviations the observed value is removed from the expected value. A bond length (or angle) with $|Z| > 5$ is considered an outlier worth inspection. RMSZ is the root-mean-square of all Z scores of the bond lengths (or angles).

Mol	Chain	Bond lengths		Bond angles	
		RMSZ	#$\|Z\|$ >5	RMSZ	#$\|Z\|$ >5
1	A	0.54	0/4642	0.69	2/6252 (0.0%)
1	B	0.56	1/4616 (0.0%)	0.69	0/6215
All	All	0.55	1/9258 (0.0%)	0.69	2/12467 (0.0%)

All (1) bond length outliers are listed below:

Mol	Chain	Res	Type	Atoms	Z	Observed(Å)	Ideal(Å)
1	B	157	CYS	CB-SG	-5.69	1.72	1.81

All (2) bond angle outliers are listed below:

Mol	Chain	Res	Type	Atoms	Z	Observed(^{o})	Ideal(^{o})
1	A	363[A]	ASP	CB-CG-OD1	5.25	123.02	118.30
1	A	363[B]	ASP	CB-CG-OD1	5.25	123.02	118.30

There are no chirality outliers.

There are no planarity outliers.

5.2 Too-close contacts (i)

In the following table, the Non-H and H(model) columns list the number of non-hydrogen atoms and hydrogen atoms in the chain respectively. The H(added) column lists the number of hydrogen atoms added and optimized by MolProbity. The Clashes column lists the number of clashes within the asymmetric unit, whereas Symm-Clashes lists symmetry-related clashes.

Mol	Chain	Non-H	H(model)	H(added)	Clashes	Symm-Clashes
1	A	4565	0	4512	32	0
1	B	4543	0	4489	48	0
2	A	17	0	5	1	0

Continued on next page...

For Manuscript Review

Continued from previous page...

Mol	Chain	Non-H	H(model)	H(added)	Clashes	Symm-Clashes
2	B	17	0	5	1	0
3	A	32	0	0	1	0
3	B	32	0	0	0	0
4	A	4	0	0	0	0
4	B	4	0	0	0	0
5	A	2	0	0	1	0
5	B	2	0	0	0	0
6	A	6	0	7	0	0
6	B	6	0	8	0	0
7	A	1	0	0	0	0
7	B	2	0	0	0	0
8	A	4	0	0	0	0
8	B	4	0	0	0	0
9	A	649	0	0	3	3
9	B	602	0	0	10	3
All	All	10492	0	9026	77	3

The all-atom clashscore is defined as the number of clashes found per 1000 atoms (including hydrogen atoms). The all-atom clashscore for this structure is 4.

All (77) close contacts within the same asymmetric unit are listed below, sorted by their clash magnitude.

Atom-1	Atom-2	Interatomic distance (Å)	Clash overlap (Å)
1:A:211:MET:HE2	9:A:710:HOH:O	1.71	0.88
1:A:483[A]:SER:OG	1:A:485:MET:HG3	1.79	0.83
1:B:556:PHE:HE1	1:B:567:ILE:HD11	1.47	0.78
1:A:277:MET:HE1	1:A:543:HIS:HA	1.71	0.72
1:A:563:ARG:HG2	1:A:567:ILE:HD12	1.75	0.68
1:B:513[A]:ASN:ND2	9:B:704:HOH:O	2.28	0.67
1:B:211:MET:CE	1:B:387:MET:HG3	2.26	0.66
1:B:282[A]:GLU:OE1	9:B:701:HOH:O	2.13	0.66
1:A:84:LYS:CE	1:A:118:ALA:HA	2.26	0.65
1:A:539:LYS:HD2	1:A:544:GLU:HB3	1.77	0.65
1:B:131:GLU:H	1:B:131:GLU:CD	2.03	0.62
1:B:211:MET:HE1	1:B:387:MET:HG3	1.82	0.61
1:B:571:LYS:HE3	9:B:742:HOH:O	2.00	0.61
1:A:39[A]:CYS:SG	1:A:159:LYS:HE3	2.42	0.60
1:A:43:ILE:HG12	1:B:485:MET:HG2	1.85	0.59
1:B:482:LYS:NZ	9:B:703:HOH:O	2.22	0.57
1:B:211:MET:HE3	1:B:383:GLU:HB3	1.86	0.57
1:A:84:LYS:HE3	1:A:118:ALA:HA	1.85	0.57

Continued on next page...

For Manuscript Review

Continued from previous page...

Atom-1	Atom-2	Interatomic distance (Å)	Clash overlap (Å)
1:A:84:LYS:HE2	1:A:118:ALA:HA	1.86	0.57
1:A:573:LYS:NZ	9:A:707:HOH:O	2.38	0.55
1:B:211:MET:HE1	1:B:383:GLU:O	2.06	0.55
1:B:70:MET:HE3	1:B:72:ILE:HD11	1.90	0.54
1:B:295[B]:MET:HG2	1:B:493:PHE:HD1	1.72	0.54
1:B:277[A]:MET:HG3	1:B:548:LEU:HD21	1.89	0.54
1:B:353[B]:MET:HE2	1:B:358:LYS:HD2	1.91	0.53
1:B:513[A]:ASN:OD1	1:B:515:LYS:HE2	2.09	0.53
1:B:9:VAL:HB	1:B:11:PHE:CE1	2.44	0.53
1:B:295[B]:MET:HG2	1:B:493:PHE:CD1	2.43	0.52
1:A:456:GLU:HG2	1:B:43:ILE:HA	1.93	0.49
1:B:211:MET:HE2	1:B:387:MET:HG3	1.94	0.49
1:A:84:LYS:C	1:A:84:LYS:HD3	2.31	0.49
1:A:138:LYS:HD3	1:A:363[B]:ASP:HB3	1.95	0.49
1:B:183:LYS:HE2	1:B:188:THR:HG22	1.94	0.49
1:B:10:GLN:NE2	1:B:11:PHE:O	2.46	0.49
1:B:343:ASP:OD1	9:B:702:HOH:O	2.20	0.48
1:B:282[A]:GLU:HG2	1:B:295[A]:MET:SD	2.53	0.48
1:A:127:LYS:HD2	1:A:127:LYS:H	1.77	0.48
1:A:392:LYS:HA	1:A:392:LYS:HD2	1.68	0.48
1:B:111:LYS:N	1:B:111:LYS:HD3	2.29	0.48
1:B:238:GLY:O	1:B:243:MET:HB2	2.14	0.48
1:A:277:MET:HE1	1:A:543:HIS:CA	2.41	0.48
1:B:532[A]:ASN:ND2	9:B:719:HOH:O	2.46	0.47
1:A:277:MET:HE3	1:A:277:MET:HB2	1.61	0.47
1:A:4:ILE:HD12	1:A:70:MET:HB2	1.95	0.47
1:A:343:ASP:OD2	1:A:345:LYS:HE3	2.13	0.47
1:B:224:HIS:CD2	1:B:342:LEU:HD11	2.49	0.47
1:A:283:LEU:O	1:A:287:ILE:HG13	2.15	0.46
1:B:336:TYR:CE2	1:B:342:LEU:HD12	2.50	0.46
1:B:4:ILE:HD12	1:B:6:ILE:HG13	1.98	0.46
1:B:224:HIS:CD2	1:B:342:LEU:CD1	2.99	0.46
1:A:503:CYS:CB	2:A:601:402:C4	2.93	0.46
1:A:394:PRO:HB2	1:A:397:LYS:HD2	1.96	0.46
1:B:503:CYS:CB	2:B:601:402:C4	2.94	0.46
1:A:43:ILE:HD13	1:B:488:GLU:CD	2.37	0.45
1:B:515:LYS:HE2	1:B:515:LYS:H	1.83	0.43
1:B:556:PHE:CE1	1:B:567:ILE:HD11	2.39	0.43
1:A:286:ARG:HD3	1:A:292:PRO:O	2.18	0.43
1:A:127:LYS:HD2	1:A:127:LYS:N	2.33	0.43
1:B:179:ALA:HB1	1:B:188:THR:HG21	2.00	0.43

Continued on next page...

For Manuscript Review

Continued from previous page...

Atom-1	Atom-2	Interatomic distance (Å)	Clash overlap (Å)
1:B:117:LYS:NZ	9:B:728:HOH:O	2.52	0.43
1:B:128:ASP:O	1:B:131:GLU:OE2	2.37	0.42
1:B:353[B]:MET:O	1:B:380:THR:HA	2.19	0.42
1:B:301:PRO:HG2	1:B:357[B]:SER:OG	2.20	0.42
1:B:2:LYS:O	1:B:12:ASN:HA	2.20	0.42
1:B:324:PRO:HA	1:B:327:ILE:HG22	2.01	0.42
1:B:386:LYS:HE2	9:B:1106:HOH:O	2.19	0.42
1:B:4:ILE:HD11	1:B:22:PHE:CE1	2.55	0.42
1:A:127:LYS:HE2	9:A:1146:HOH:O	2.20	0.41
1:A:324:PRO:HA	1:A:327:ILE:HG22	2.03	0.41
1:A:301:PRO:HD2	3:A:602:SF4:S2	2.61	0.41
1:B:519:LYS:HB3	1:B:519:LYS:HE3	1.80	0.41
1:A:480:PHE:O	1:A:485:MET:HB2	2.21	0.41
1:B:550:LYS:NZ	9:B:712:HOH:O	2.37	0.40
1:A:272:ALA:HA	1:A:324:PRO:HG3	2.04	0.40
1:A:299:CYS:HB2	5:A:607:CYN:N	2.36	0.40
1:B:272:ALA:HA	1:B:324:PRO:HG3	2.03	0.40
1:B:488:GLU:HB3	9:B:737:HOH:O	2.21	0.40

All (3) symmetry-related close contacts are listed below. The label for Atom-2 includes the symmetry operator and encoded unit-cell translations to be applied.

Atom-1	Atom-2	Interatomic distance (Å)	Clash overlap (Å)
9:A:1193:HOH:O	9:B:1106:HOH:O[2_555]	2.13	0.07
9:A:1193:HOH:O	9:B:1020:HOH:O[2_555]	2.18	0.02
9:A:1071:HOH:O	9:B:1162:HOH:O[2_445]	2.19	0.01

5.3 Torsion angles (i)

5.3.1 Protein backbone (i)

In the following table, the Percentiles column shows the percent Ramachandran outliers of the chain as a percentile score with respect to all X-ray entries followed by that with respect to entries of similar resolution.

The Analysed column shows the number of residues for which the backbone conformation was analysed, and the total number of residues.

Mol	Chain	Analysed	Favoured	Allowed	Outliers	Percentiles
1	A	586/584 (100%)	570 (97%)	15 (3%)	1 (0%)	47 21

Continued on next page...

For Manuscript Review

Continued from previous page...

Mol	Chain	Analysed	Favoured	Allowed	Outliers	Percentiles	
1	B	583/584 (100%)	564 (97%)	19 (3%)	0	100	100
All	All	1169/1168 (100%)	1134 (97%)	34 (3%)	1 (0%)	51	23

All (1) Ramachandran outliers are listed below:

Mol	Chain	Res	Type
1	A	172	ASN

5.3.2 Protein sidechains ⓘ

In the following table, the Percentiles column shows the percent sidechain outliers of the chain as a percentile score with respect to all X-ray entries followed by that with respect to entries of similar resolution.

The Analysed column shows the number of residues for which the sidechain conformation was analysed, and the total number of residues.

Mol	Chain	Analysed	Rotameric	Outliers	Percentiles	
1	A	503/499 (101%)	501 (100%)	2 (0%)	91	78
1	B	501/499 (100%)	498 (99%)	3 (1%)	86	70
All	All	1004/998 (101%)	999 (100%)	5 (0%)	88	74

All (5) residues with a non-rotameric sidechain are listed below:

Mol	Chain	Res	Type
1	A	279	GLU
1	A	456	GLU
1	B	515	LYS
1	B	571	LYS
1	B	573	LYS

Sometimes sidechains can be flipped to improve hydrogen bonding and reduce clashes. There are no such sidechains identified.

5.3.3 RNA ⓘ

There are no RNA molecules in this entry.

For Manuscript Review

5.4 Non-standard residues in protein, DNA, RNA chains (i)

There are no non-standard protein/DNA/RNA residues in this entry.

5.5 Carbohydrates (i)

There are no monosaccharides in this entry.

5.6 Ligand geometry (i)

Of 27 ligands modelled in this entry, 11 are monoatomic - leaving 16 for Mogul analysis.

In the following table, the Counts columns list the number of bonds (or angles) for which Mogul statistics could be retrieved, the number of bonds (or angles) that are observed in the model and the number of bonds (or angles) that are defined in the Chemical Component Dictionary. The Link column lists molecule types, if any, to which the group is linked. The Z score for a bond length (or angle) is the number of standard deviations the observed value is removed from the expected value. A bond length (or angle) with $|Z| > 2$ is considered an outlier worth inspection. RMSZ is the root-mean-square of all Z scores of the bond lengths (or angles).

Mol	Type	Chain	Res	Link	Bond lengths			Bond angles		
					Counts	RMSZ	$\#\|Z\| > 2$	Counts	RMSZ	$\#\|Z\| > 2$
3	SF4	B	602	1	0,12,12	-	-	-		
3	SF4	A	604	1	0,12,12	-	-	-		
5	CYN	B	608	-	0,1,1	-	-	-		
2	402	B	601	1	13,19,19	2.09	3 (23%)	2,36,36	2.23	1 (50%)
3	SF4	B	604	1	0,12,12	-	-	-		
3	SF4	A	605	1	0,12,12	-	-	-		
3	SF4	B	605	1	0,12,12	-	-	-		
4	FES	B	606	1	0,4,4	-	-	-		
5	CYN	A	607	-	0,1,1	-	-	-		
6	GOL	B	609	-	5,5,5	1.29	1 (20%)	5,5,5	1.09	0
6	GOL	A	608	-	5,5,5	1.79	1 (20%)	5,5,5	0.70	0
2	402	A	601	1	13,19,19	1.72	4 (30%)	2,36,36	0.87	0
3	SF4	A	603	1	0,12,12	-	-	-		
3	SF4	A	602	1	0,12,12	-	-	-		
4	FES	A	606	1	0,4,4	-	-	-		
3	SF4	B	603	1	0,12,12	-	-	-		

In the following table, the Chirals column lists the number of chiral outliers, the number of chiral centers analysed, the number of these observed in the model and the number defined in the Chemical Component Dictionary. Similar counts are reported in the Torsion and Rings columns. '-' means no outliers of that kind were identified.

For Manuscript Review

Mol	Type	Chain	Res	Link	Chirals	Torsions	Rings
3	SF4	B	602	1	-	-	0/6/5/5
3	SF4	A	604	1	-	-	0/6/5/5
2	402	A	601	1	-	-	0/5/3/3
2	402	B	601	1	-	-	0/5/3/3
3	SF4	B	604	1	-	-	0/6/5/5
3	SF4	A	605	1	-	-	0/6/5/5
3	SF4	B	605	1	-	-	0/6/5/5
4	FES	B	606	1	-	-	0/1/1/1
6	GOL	A	608	-	-	0/4/4/4	-
6	GOL	B	609	-	-	3/4/4/4	-
3	SF4	A	603	1	-	-	0/6/5/5
3	SF4	A	602	1	-	-	0/6/5/5
4	FES	A	606	1	-	-	0/1/1/1
3	SF4	B	603	1	-	-	0/6/5/5

All (9) bond length outliers are listed below:

Mol	Chain	Res	Type	Atoms	Z	Observed(Å)	Ideal(Å)
2	B	601	402	S1-FE2	5.26	2.33	2.26
2	B	601	402	S1-FE1	3.70	2.31	2.26
2	A	601	402	S1-FE2	3.39	2.31	2.26
6	A	608	GOL	O2-C2	-3.08	1.34	1.43
2	A	601	402	C2-S2	2.49	1.89	1.85
2	A	601	402	S1-FE1	2.35	2.29	2.26
2	A	601	402	C3-FE1	-2.12	1.72	1.79
6	B	609	GOL	O2-C2	-2.07	1.37	1.43
2	B	601	402	C2-S2	2.05	1.88	1.85

All (1) bond angle outliers are listed below:

Mol	Chain	Res	Type	Atoms	Z	Observed(^{o})	Ideal(^{o})
2	B	601	402	S2-C2-N1	-2.71	109.72	117.18

There are no chirality outliers.

All (3) torsion outliers are listed below:

Mol	Chain	Res	Type	Atoms
6	B	609	GOL	O1-C1-C2-C3
6	B	609	GOL	O1-C1-C2-O2
6	B	609	GOL	O2-C2-C3-O3

There are no ring outliers.

For Manuscript Review

4 monomers are involved in 4 short contacts:

Mol	Chain	Res	Type	Clashes	Symm-Clashes
2	B	601	402	1	0
5	A	607	CYN	1	0
2	A	601	402	1	0
3	A	602	SF4	1	0

The following is a two-dimensional graphical depiction of Mogul quality analysis of bond lengths, bond angles, torsion angles, and ring geometry for all instances of the Ligand of Interest. In addition, ligands with molecular weight > 250 and outliers as shown on the validation Tables will also be included. For torsion angles, if less then 5% of the Mogul distribution of torsion angles is within 10 degrees of the torsion angle in question, then that torsion angle is considered an outlier. Any bond that is central to one or more torsion angles identified as an outlier by Mogul will be highlighted in the graph. For rings, the root-mean-square deviation (RMSD) between the ring in question and similar rings identified by Mogul is calculated over all ring torsion angles. If the average RMSD is greater than 60 degrees and the minimal RMSD between the ring in question and any Mogul-identified rings is also greater than 60 degrees, then that ring is considered an outlier. The outliers are highlighted in purple. The color gray indicates Mogul did not find sufficient equivalents in the CSD to analyse the geometry.

Ligand CYN B 608

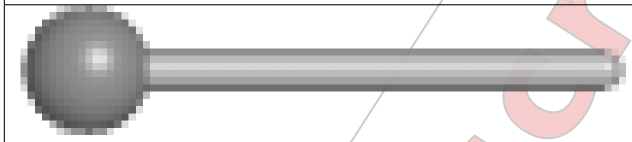

Bond lengths

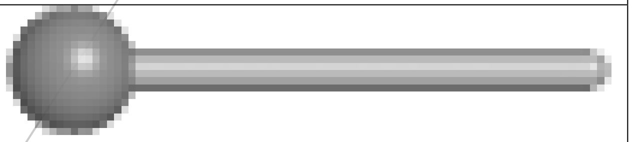

Bond angles

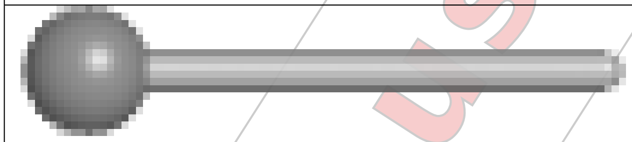

Torsions

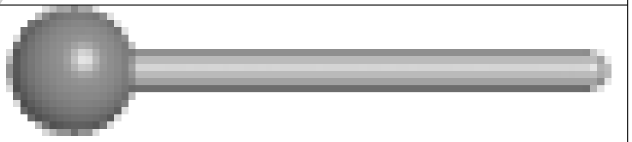

Rings

For Manuscript Review

Ligand 402 B 601

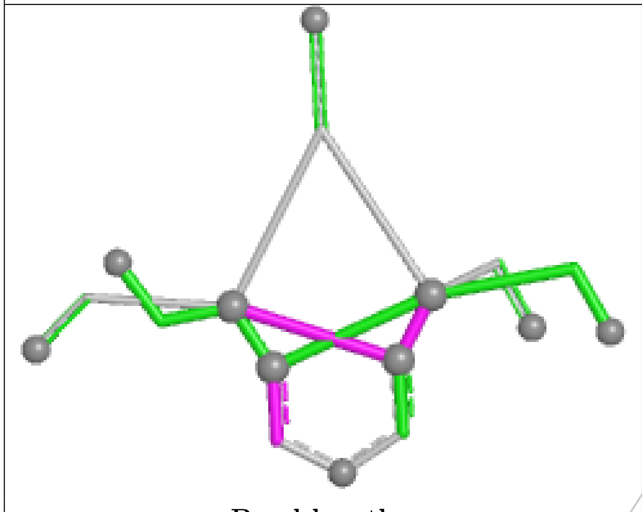
Bond lengths

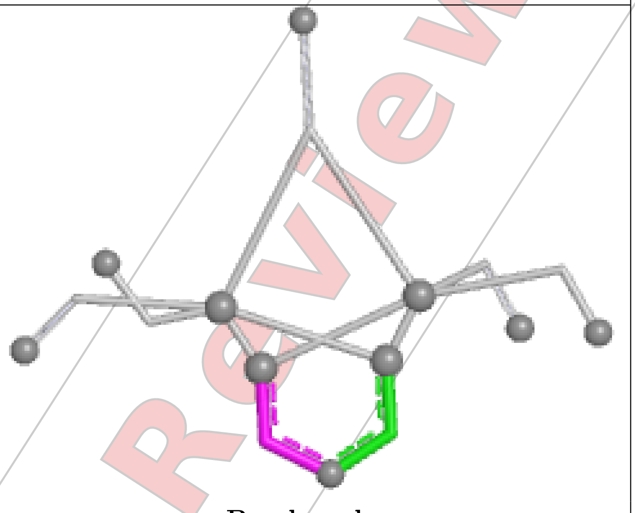
Bond angles

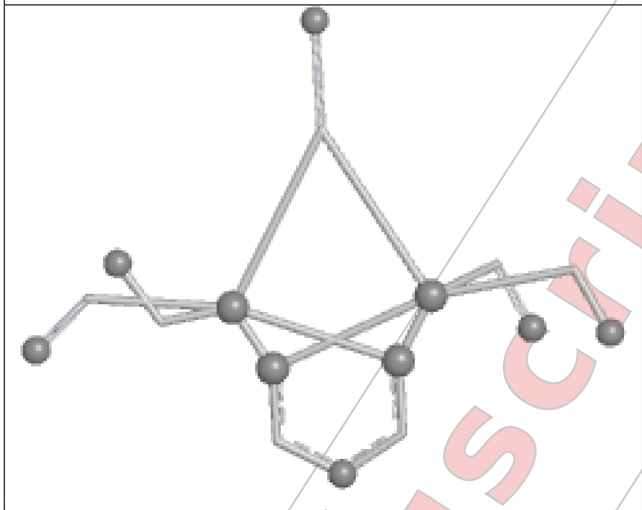
Torsions

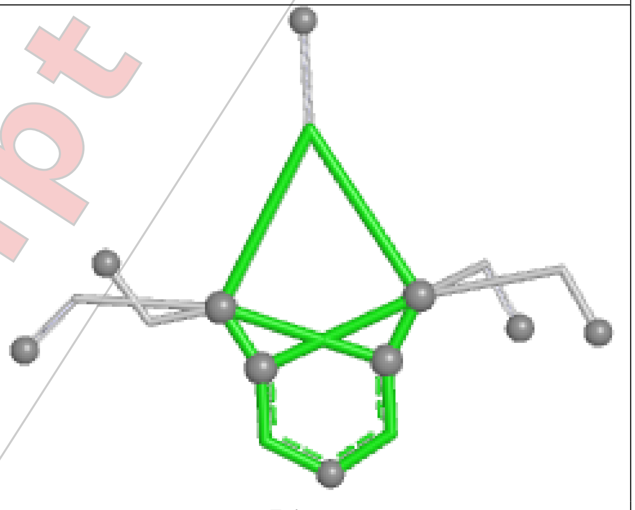
Rings

Ligand CYN A 607

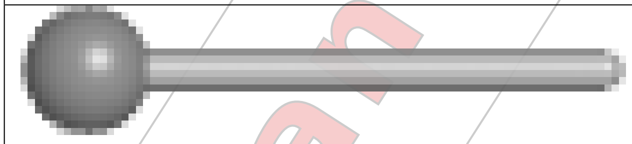
Bond lengths

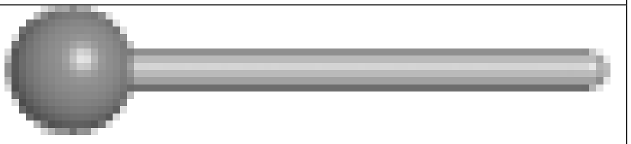
Bond angles

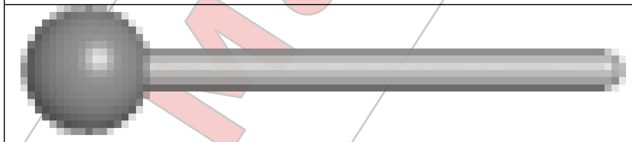
Torsions

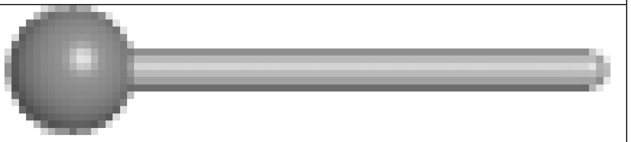
Rings

For Manuscript Review

Ligand 402 A 601

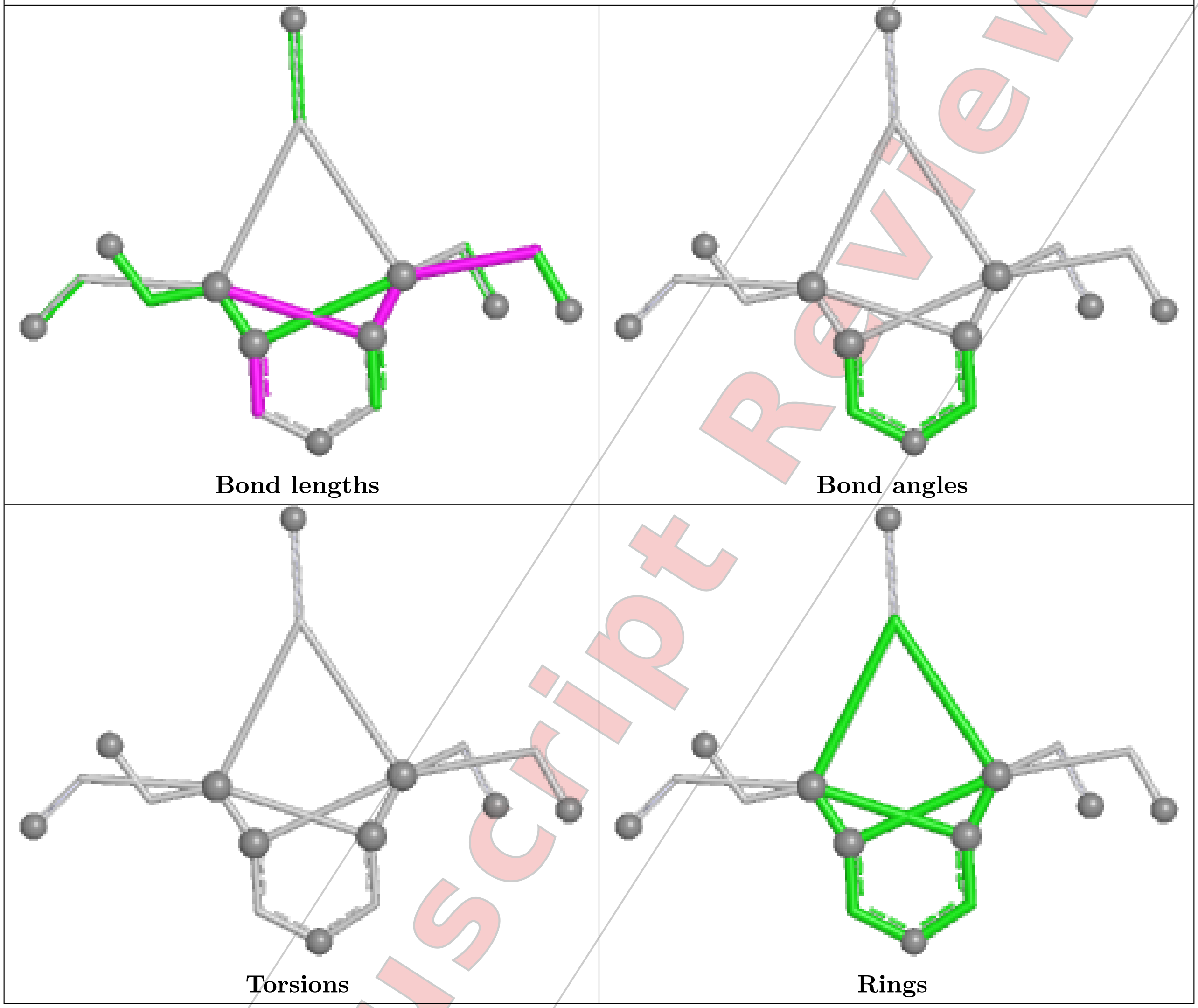

5.7 Other polymers (i)

There are no such residues in this entry.

5.8 Polymer linkage issues (i)

There are no chain breaks in this entry.

For Manuscript Review

6 Fit of model and data (i)

6.1 Protein, DNA and RNA chains (i)

In the following table, the column labelled '#RSRZ> 2' contains the number (and percentage) of RSRZ outliers, followed by percent RSRZ outliers for the chain as percentile scores relative to all X-ray entries and entries of similar resolution. The OWAB column contains the minimum, median, 95th percentile and maximum values of the occupancy-weighted average B-factor per residue. The column labelled 'Q< 0.9' lists the number of (and percentage) of residues with an average occupancy less than 0.9.

Mol	Chain	Analysed	<RSRZ>	#RSRZ>2			OWAB(Å^2)	Q<0.9
1	A	581/584 (99%)	0.14	16 (2%)	53	52	13, 21, 38, 65	0
1	B	573/584 (98%)	0.56	52 (9%)	9	8	12, 20, 58, 80	0
All	All	1154/1168 (98%)	0.35	68 (5%)	22	20	12, 21, 47, 80	0

All (68) RSRZ outliers are listed below:

Mol	Chain	Res	Type	RSRZ
1	B	5	ILE	13.3
1	B	3	THR	12.1
1	B	9	VAL	11.4
1	B	4	ILE	9.8
1	B	11	PHE	9.5
1	B	8	GLY	8.4
1	B	69	GLY	8.2
1	B	6	ILE	7.9
1	B	22	PHE	7.6
1	B	55	GLY	7.0
1	B	71	ILE	6.9
1	B	10	GLN	6.8
1	B	574	LYS	6.3
1	B	56	THR	6.0
1	B	68	ASP	5.9
1	B	27	ASN	5.7
1	B	12	ASN	5.3
1	B	13	THR	5.3
1	B	2	LYS	5.2
1	B	57	GLY	4.4
1	B	14	ASP	4.3
1	B	70	MET	4.2
1	B	67	GLU	4.2
1	B	127	LYS	4.1

Continued on next page...

For Manuscript Review

Continued from previous page...

Mol	Chain	Res	Type	RSRZ
1	B	23	ALA	3.9
1	B	72	ILE	3.8
1	B	75	ASN	3.8
1	B	573	LYS	3.8
1	B	25	ASP	3.8
1	B	7	ASN	3.8
1	A	580	PRO	3.6
1	B	54	GLU	3.6
1	B	26	ASN	3.6
1	A	437	ASN	3.5
1	B	131	GLU	3.4
1	A	440	LEU	3.3
1	A	1	MET	3.2
1	B	73	ASN	3.1
1	B	66	ILE	3.1
1	A	537	LEU	3.0
1	A	538	SER	2.9
1	B	52	GLU	2.9
1	B	28	ILE	2.8
1	B	499	CYS	2.7
1	B	16	ASP	2.6
1	B	58	LEU	2.5
1	B	29	ASP	2.5
1	A	577	TRP	2.4
1	A	39[A]	CYS	2.4
1	B	130	THR	2.3
1	B	172	ASN	2.3
1	A	55	GLY	2.3
1	A	287	ILE	2.3
1	B	39[A]	CYS	2.3
1	B	463	ASN	2.2
1	B	78	ALA	2.2
1	A	463	ASN	2.1
1	B	30	ILE	2.1
1	B	441	GLU	2.1
1	A	14	ASP	2.1
1	A	290	ASN	2.1
1	A	392	LYS	2.1
1	A	573	LYS	2.1
1	B	164	TYR	2.1
1	A	478	PHE	2.1
1	B	157	CYS	2.1

Continued on next page...

For Manuscript Review

Continued from previous page...

Mol	Chain	Res	Type	RSRZ
1	B	128	ASP	2.0
1	B	74	THR	2.0

6.2 Non-standard residues in protein, DNA, RNA chains (i)

There are no non-standard protein/DNA/RNA residues in this entry.

6.3 Carbohydrates (i)

There are no monosaccharides in this entry.

6.4 Ligands (i)

In the following table, the Atoms column lists the number of modelled atoms in the group and the number defined in the chemical component dictionary. The B-factors column lists the minimum, median, 95th percentile and maximum values of B factors of atoms in the group. The column labelled 'Q< 0.9' lists the number of atoms with occupancy less than 0.9.

Mol	Type	Chain	Res	Atoms	RSCC	RSR	B-factors(Å^2)	Q<0.9
8	MG	B	614	1/1	0.70	0.19	45,45,45,45	0
8	MG	A	611	1/1	0.73	0.35	49,49,49,49	0
8	MG	A	610	1/1	0.74	0.29	34,34,34,34	0
5	CYN	A	607	2/2	0.95	0.09	16,16,16,17	0
6	GOL	A	608	6/6	0.96	0.08	20,21,25,27	0
6	GOL	B	609	6/6	0.97	0.07	22,23,26,28	0
8	MG	B	613	1/1	0.98	0.20	38,38,38,38	0
8	MG	A	612	1/1	0.98	0.07	19,19,19,19	0
5	CYN	B	608	2/2	0.99	0.05	15,15,15,15	0
2	402	B	601	17/17	0.99	0.07	9,14,16,17	0
3	SF4	A	603	8/8	0.99	0.06	14,15,15,15	0
7	CL	A	609	1/1	0.99	0.05	29,29,29,29	0
7	CL	B	610	1/1	0.99	0.05	24,24,24,24	0
7	CL	B	611	1/1	0.99	0.03	28,28,28,28	0
3	SF4	A	604	8/8	0.99	0.05	14,15,16,16	0
3	SF4	A	605	8/8	0.99	0.04	19,19,20,21	0
3	SF4	B	602	8/8	0.99	0.07	13,13,14,15	0
8	MG	A	613	1/1	0.99	0.04	21,21,21,21	0
8	MG	B	607	1/1	0.99	0.04	20,20,20,20	0
8	MG	B	612	1/1	0.99	0.10	25,25,25,25	0
3	SF4	B	605	8/8	0.99	0.04	20,22,23,23	0

Continued on next page...

For Manuscript Review

Continued from previous page...

Mol	Type	Chain	Res	Atoms	RSCC	RSR	B-factors(Å^2)	Q<0.9
2	402	A	601	17/17	0.99	0.06	12,16,17,18	0
3	SF4	B	603	8/8	1.00	0.06	14,14,15,15	0
3	SF4	B	604	8/8	1.00	0.05	15,16,16,17	0
3	SF4	A	602	8/8	1.00	0.07	13,14,14,14	0
4	FES	A	606	4/4	1.00	0.05	16,16,16,16	0
4	FES	B	606	4/4	1.00	0.04	20,21,21,23	0

The following is a graphical depiction of the model fit to experimental electron density of all instances of the Ligand of Interest. In addition, ligands with molecular weight > 250 and outliers as shown on the geometry validation Tables will also be included. Each fit is shown from different orientation to approximate a three-dimensional view.

Electron density around CYN A 607:

2mF$_o$-DF$_c$ (at 0.7 rmsd) in gray
mF$_o$-DF$_c$ (at 3 rmsd) in purple (negative)
and green (positive)

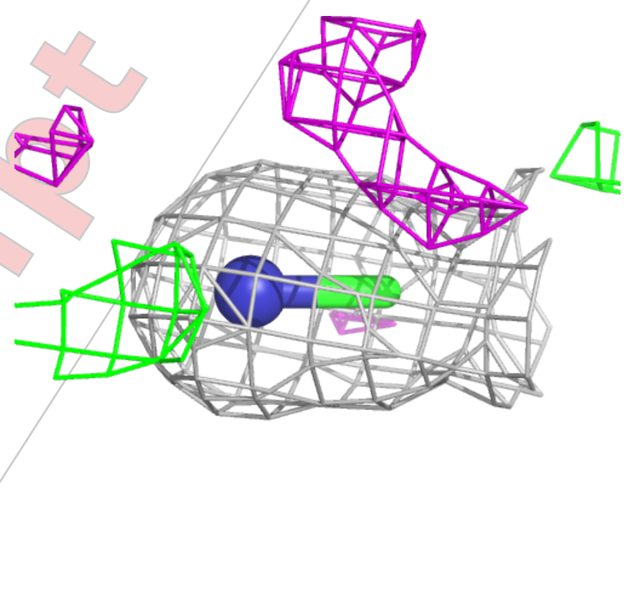

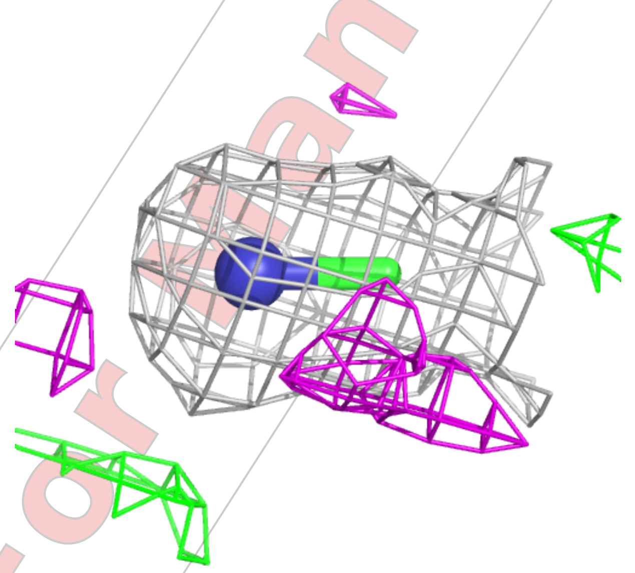

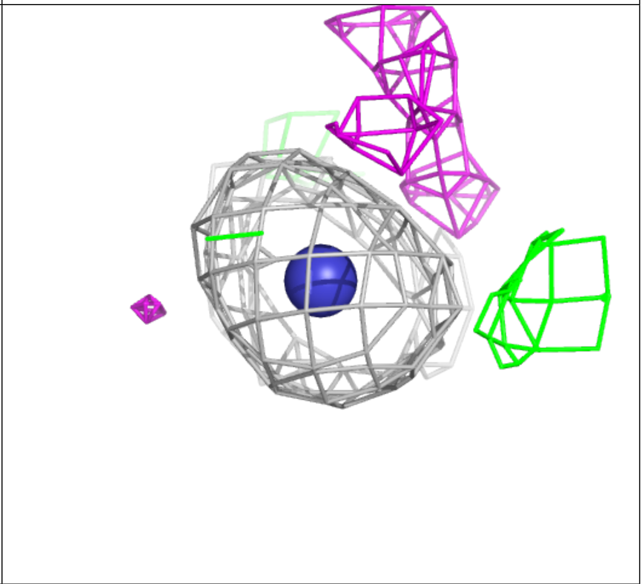

For Manuscript Review

Electron density around CYN B 608:

$2mF_o$-DF_c (at 0.7 rmsd) in gray
mF_o-DF_c (at 3 rmsd) in purple (negative)
and green (positive)

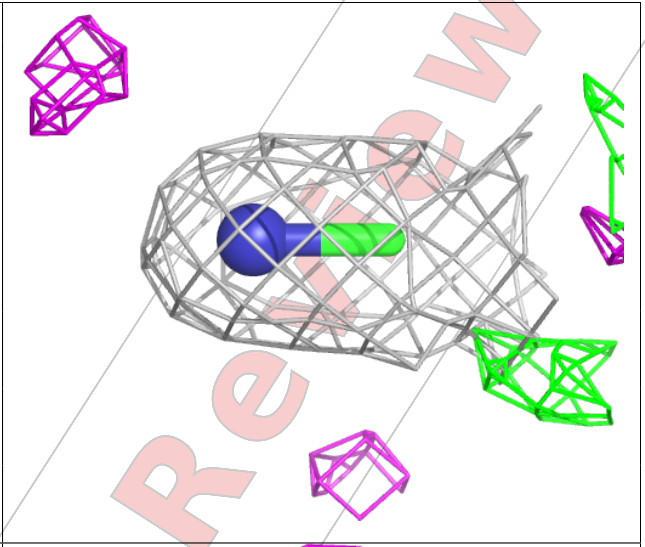

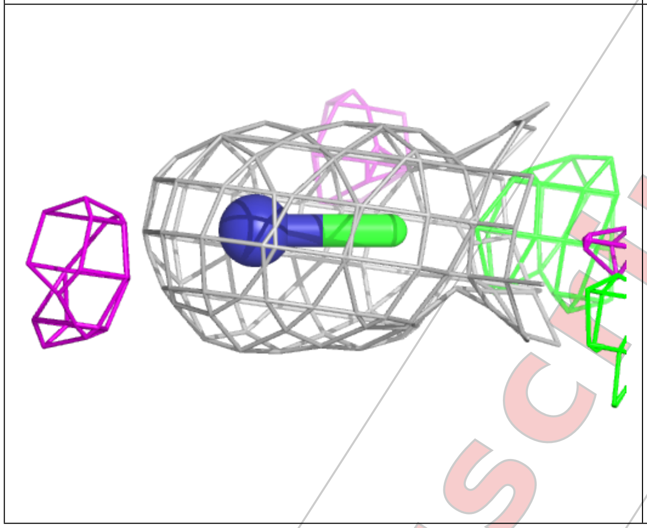

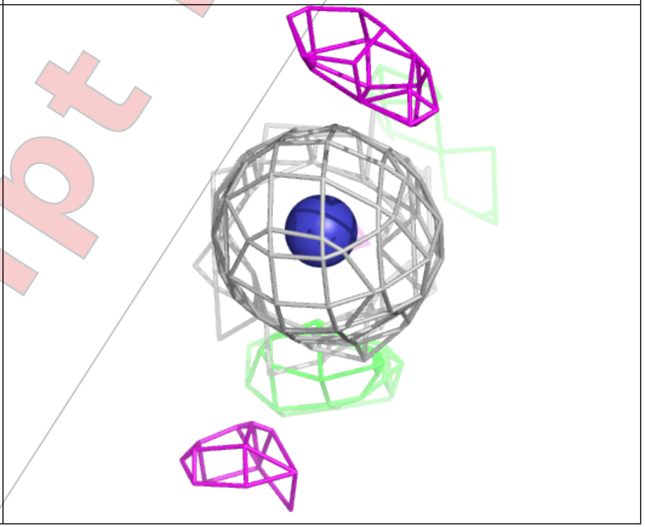

For Manuscript Review

Electron density around 402 B 601:

2mF$_o$-DF$_c$ (at 0.7 rmsd) in gray
mF$_o$-DF$_c$ (at 3 rmsd) in purple (negative)
and green (positive)

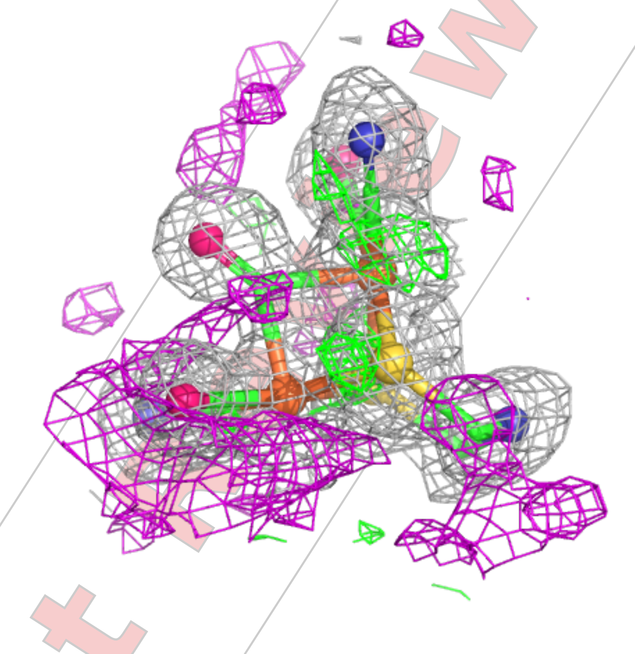

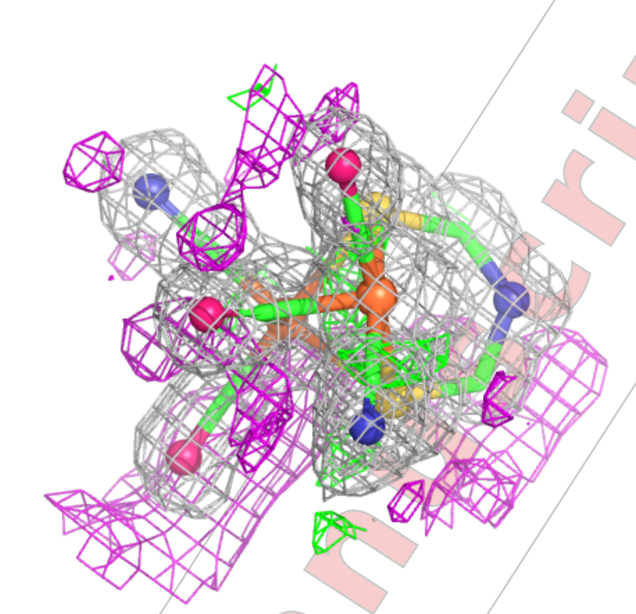

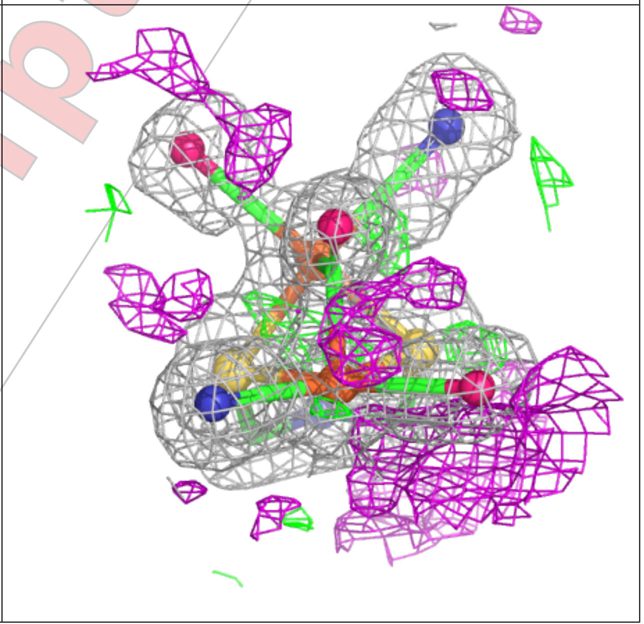

For Manuscript Review

Electron density around 402 A 601:

$2mF_o$-DF_c (at 0.7 rmsd) in gray
mF_o-DF_c (at 3 rmsd) in purple (negative)
and green (positive)

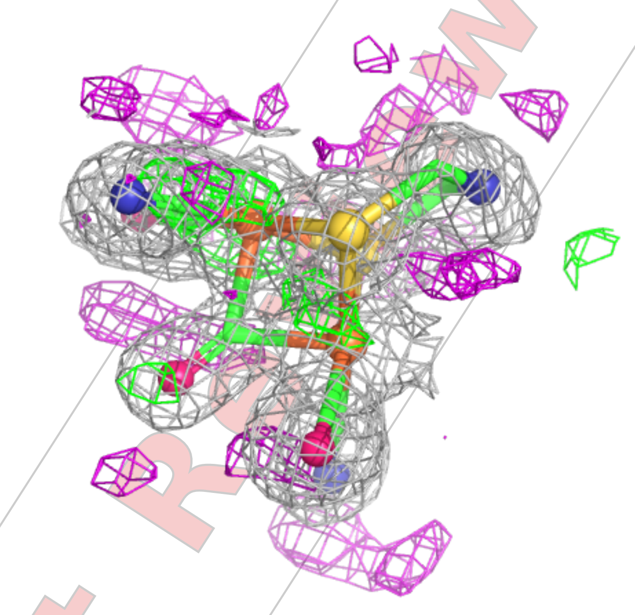

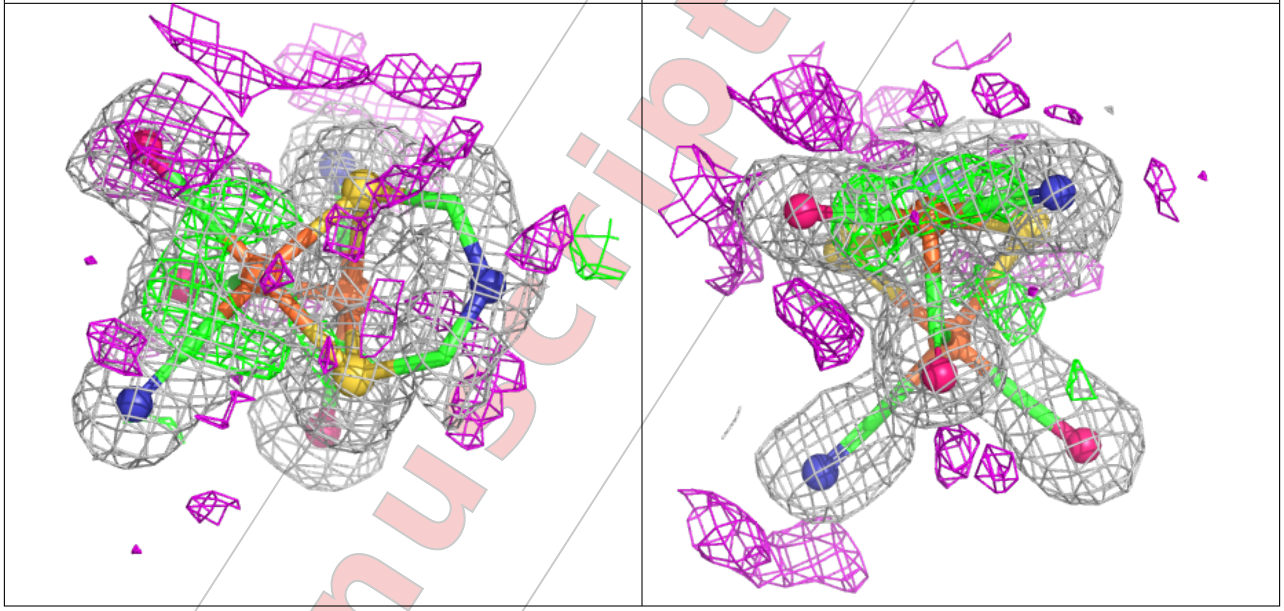

6.5 Other polymers (i)

There are no such residues in this entry.

For Manuscript Review
